# Using Genomics to Shape the Definition of the Agglutinin-Like Sequence (*ALS*) Family in the Saccharomycetales

**DOI:** 10.3389/fcimb.2021.794529

**Published:** 2021-12-14

**Authors:** Soon-Hwan Oh, Klaus Schliep, Allyson Isenhower, Rubi Rodriguez-Bobadilla, Vien M. Vuong, Christopher J. Fields, Alvaro G. Hernandez, Lois L. Hoyer

**Affiliations:** ^1^Department of Pathobiology, College of Veterinary Medicine, University of Illinois at Urbana-Champaign, Urbana, IL, United States; ^2^Institute of Environmental Biotechnology, Graz University of Technology, Graz, Austria; ^3^Department of Biology, Millikin University, Decatur, IL, United States; ^4^Department of Computer Science, University of Illinois at Urbana-Champaign, Urbana, IL, United States; ^5^Roy J. Carver Biotechnology Center, University of Illinois at Urbana-Champaign, Urbana, IL, United States

**Keywords:** ALS genes, adhesion, comparative genomics, protein structure, fungi, repeated sequences

## Abstract

The *Candida albicans* agglutinin-like sequence (*ALS*) family is studied because of its contribution to cell adhesion, fungal colonization, and polymicrobial biofilm formation. The goal of this work was to derive an accurate census and sequence for *ALS* genes in pathogenic yeasts and other closely related species, while probing the boundaries of the *ALS* family within the Order Saccharomycetales. Bioinformatic methods were combined with laboratory experimentation to characterize 47 novel *ALS* loci from 8 fungal species. AlphaFold predictions suggested the presence of a conserved N-terminal adhesive domain (NT-Als) structure in all Als proteins reported to date, as well as in *S. cerevisiae* alpha-agglutinin (Sag1). *Lodderomyces elongisporus*, *Meyerozyma guilliermondii*, and *Scheffersomyces stipitis* were notable because each species had genes with *C. albicans ALS* features, as well as at least one that encoded a Sag1-like protein. Detection of recombination events between the *ALS* family and gene families encoding other cell-surface proteins such as Iff/Hyr and Flo suggest widespread domain swapping with the potential to create cell-surface diversity among yeast species. Results from the analysis also revealed subtelomeric *ALS* genes, *ALS* pseudogenes, and the potential for yeast species to secrete their own soluble adhesion inhibitors. Information presented here supports the inclusion of *SAG1* in the *ALS* family and yields many experimental hypotheses to pursue to further reveal the nature of the *ALS* family.

## Introduction

Most knowledge about agglutinin-like sequence (Als) protein structure and function comes from the study of *Candida albicans* (reviewed in Hoyer and Cota, 2016). *C. albicans* Als proteins are adhesins that promote interactions between fungi and host cells, establishing colonization that provides the opportunity to cause disease. Als proteins also function in *C. albicans* adhesion to abiotic surfaces such as indwelling medical devices, and between fungi and other microbes such as bacteria, seeding polymicrobial biofilm development (Peters et al., 2012).

Using Als protein features known at the time, Hoyer and Cota (2016) suggested the NT/T/TR/CT model for proteins in the Als family with each protein possessing an NT-Als adhesive domain (NT; Lin et al., 2014), followed by a Thr-rich region (T), tandemly repeated sequences (TR), and a C-terminal (CT) domain rich in Ser and Thr. Other key features include a secretory signal peptide and GPI anchor addition sequence that direct the mature protein to β-1,6-glucan crosslinkage in the fungal cell wall (Lu et al., 1994). Display of the NT-Als adhesive domain on the cell surface is key for its ability to interact with other cells and materials (Lin et al., 2014).

The crystallographic structure of the NT-Als adhesive domain revealed protein folding driven by formation of 4 disulfide bonds (8 Cys); an invariant Lys is located at the end of the NT-Als binding pocket (Salgado et al., 2011). The positively charged Lys establishes a salt bridge with the C-terminal carboxylic acid of an incoming peptide ligand. Als binding affinity is at the micromolar level; avidity of Als protein interactions is increased by a peptide sequence with high β-aggregation potential (called the amyloid-forming region; AFR; Lipke et al., 2018).

The first *ALS* gene was named for the similarity of its predicted protein to *Saccharomyces cerevisiae* alpha-agglutinin (Sag1; Hoyer et al., 1995). At that time, considerably less DNA sequence information was available in public databases. Sag1 is an adhesin that promotes cell-cell contact between haploid yeasts during the mating process (Wojciechowicz et al., 1993). Sag1 binds the carboxyl-terminal peptide of **a**-agglutinin with nanomolar affinity (Cappellaro et al., 1994; Zhao et al., 2001). In contrast to Als proteins, the sequence of the N-terminal region of Sag1 has 6 Cys and an Arg residue at the position of the invariant Lys. The structure of Sag1 was not solved experimentally although molecular modeling and biochemical analyses contributed considerable insight into protein function and potential structure (Chen et al., 1995; Lipke et al., 1995; Grigorescu et al., 2000). Despite inherent differences between Als proteins and Sag1, various authors recognized the common features between these proteins and proposed that Sag1 is part of the Als family (Hoyer and Cota, 2016; Lipke, 2018).

The rich foundation of experimental evidence from *C. albicans* was used to define *ALS* genes and their encoded proteins in other fungal species including *Candida dubliniensis* (Jackson et al., 2009), *Candida parapsilosis* (Oh et al., 2019), *Candida orthopsilosis* (Lombardi et al., 2019), *Candida metapsilosis* (Oh et al., 2019) and *Candida tropicalis* (Oh et al., 2021). The burgeoning number of publicly available draft genome sequences provides the opportunity to locate other *ALS* sequences using a simple BLAST search. However, lack of proper gene assembly is a frequent stumbling block due to the tendency for each species to have multiple *ALS* loci and for *ALS* genes to encode long stretches of highly conserved, tandemly repeated sequences. Computer assembly of short-read sequences often breaks down in *ALS* coding regions because the length of repeated sequences is similar to the length of sequence reads (Lombardi et al., 2019). Emergence of long-read DNA sequencing technology provided datasets with more-accurate *ALS* assemblies, however, PCR amplification and Sanger sequencing are still frequently required to complete *ALS* loci (Oh et al., 2019; Oh et al., 2021).

The initial focus of this work was to derive an accurate census and sequence for the *ALS* genes in the human pathogenic Saccharomycetales species. Closely related species, including some with biotechnological importance, were also studied because their genome sequences are incorporated into available analysis tools (Maguire et al., 2013). Information presented here expands the list of known *ALS* loci, adding 47 carefully validated genes from 8 fungal species. Four long-read-based genome assemblies were generated. Predicted proteins were evaluated in the context of the model *C. albicans* Als and *S. cerevisiae* Sag1 characteristics. The availability of new, accurate structural prediction tools (Jumper et al., 2021) provided the opportunity to assess similarities within this group of proteins. Recent release of many new genome sequences for budding yeasts in the subphylum Saccharomycotina (Shen et al., 2018) allowed new insight into whether *C. albicans* Als proteins and *S. cerevisiae* Sag1 arose from a common ancestor. The resulting information redefines the *ALS* family, its diversity, and taxonomic boundaries. Information presented here also provides the basis for new hypotheses regarding Als protein function.

## Materials and Methods

### Fungal Strain and Public Genome Sequence Resources

To ensure use of authenticated materials, *Lodderomyces elongisporus* strain NRRL YB-4239 and *Spathaspora passalidarum* strain NRRL Y-27907 were obtained from the Agricultural Research Service Culture Collection (Peoria, IL; https://nrrl.ncaur.usda.gov). *Meyerozyma guilliermondii* strain ATCC 6260 and *Scheffersomyces stipitis* strain CBS 6054 (ATCC 58785) were purchased from the American Type Culture Collection (https://www.atcc.org). **Table 1** lists genome sequences used for this study.

**Table 1:** Principal genome sequences used for *ALS* gene detection.

Species	Strain	Assembly Name	GenBank Assembly Accession	Reference
*Candida albicans*	SC5314	ASM18296v3	GCA_000182965.3	(Jones et al., 2004)
*Candida dubliniensis*	CD36	ASM2694v1	GCA_000026945.1	(Jackson et al., 2009)
*Candida parapsilosis*	CDC317	ASM18276v2	GCF_000182765.2	(Butler et al., 2009)
*Candida orthopsilosis*	Co 90-125	ASM31587v1	GCA_000315875.1	(Riccombeni et al., 2012)
		ASM433491v1	GCA_004334915.1	(Lombardi et al., 2019)
*Candida metapsilosis*	ATCC 96143	UIUC_Cmeta_2.0	GCA_008904905.1	(Oh et al., 2019)
*Candida tropicalis*	MYA-3404	ASM633v3	GCA_000006335.3	(Butler et al., 2009)
		ASM694213v1	GCA_006942135.1	(Oh et al., 2021)
		ASM1731540v1	GCA_017315405.1	(Guin et al., 2020)
*Meyerozyma guilliermondii*	ATCC 6260	ASM14942v1	GCA_000149425.1	(Butler et al., 2009)
		ASM694215v1	GCA_006942155.1	This work
*Clavispora lusitaniae*	ATCC 42720	ASM383v1	GCA_000003835.1	(Butler et al., 2009)
*Scheffersomyces sitpitis*	CBS 6054	ASM20916v1	GCA_000209165.1	(Jeffries et al., 2007)
		ASM694211v1	GCA_006942115.1	This work
*Debaryomyces hansenii*	CBS 767	ASM644v2	GCA_000006445.2	(Dujon et al., 2004)
*Lodderomyces elongisporus*	NRRL YB-4239	ASM14968v1	GCA_000149685.1	(Butler et al., 2009)
		ASM1362098v1	GCA_013620985.1	This work
*Yamadazyma tenuis*	ATCC 10573	Candida tenuis v1.0	GCA_000223465.1	(Wohlbach et al., 2011)
*Spathaspora passalidarum*	NRRL Y-27907	S passalidarum v2.0	GCA_000223485.1	(Wohlbach et al., 2011)
		ASM1362096v1	GCA_013620965.1	This work
*Pichia kudriavzevii*	ATCC 6258	ASM305444v1	GCA_003054445.1	(Douglass et al., 2018)
*Candida glabrata*	CBS138	ASM254v2	GCA_000002545.2	(Dujon et al., 2004)
	BG2	ASM1421772v1	GCA_010111755.1	(Xu et al., 2020)
*Saccharomyces cerevisiae*	S288C	R64	GCA_000146045.2	(Goffeau et al., 1996)
*Candida auris*	B8441	Cand_auris_B8441_V2	GCA_002759435.2	(Lockhart et al., 2017)

### Genome Sequencing and Assembly

New genome assemblies were constructed for *L. elongisporus* NRRL YB-4239 (ASM1362098v1), *M. guilliermondii* ATCC 6260 (ASM6942125v1), *S. stipitis* CBS 6054 (ASM694211v1), and *S. passalidarum* NRRL Y-27907 (ASM362096v1) using a combination of Illumina MiSeq and Oxford Nanopore MinION data. Details of the method and genome assembly are in **Supplementary Files S1****–****S4**, respectively. Similar methods were described previously (Oh et al., 2019; Oh et al., 2021) and were reproduced here for the reader’s convenience.

Fungal cells were stored at -80°C and streaked to YPD agar plates (per liter: 10 g yeast extract, 20 g Bacto peptone, 20 g dextrose, 20 g Bacto agar). A single colony was inoculated into YPD liquid medium and the culture grown 16 h at 30°C and 200 r/min shaking. Genomic DNA was isolated according to Sherman et al. (1986). Briefly, cells were spheroplasted with zymolyase and lysed with sodium dodecyl sulfate. The lysate was extracted with phenol, DNA precipitated with isopropanol, and the final preparation treated with Proteinase K. DNA shearing was minimized by use of wide-bore pipet tips and gentle mixing. Agarose gel electrophoresis was used to verify the presence of high-molecular-weight DNA prior to subsequent library preparation.

Libraries were constructed and sequenced at the Roy J. Carver Biotechnology Center, University of Illinois at Urbana-Champaign. Individually barcoded shotgun libraries were made using the Hyper Library construction kit (Roche) and pooled in equimolar concentration. The pooled libraries were quantitated by qPCR and sequenced on one MiSeq flowcell for 251 cycles from each end of the fragment using a MiSeq 500-cycle sequencing kit (version 2). PhiX DNA was used as a spike-in control for MiSeq sequencing runs. FASTQ files were generated and demultiplexed with bcl2fastq Conversion Software (Illumina, version 2.17.1.14). MiSeq reads were quality trimmed using Trimmomatic v0.36 (Bolger et al., 2014) to remove adaptors from the 3’ end of the reads prior to assembly, retaining all reads greater than or equal to 50 nt in length.

Libraries for Oxford Nanopore long-read sequencing used 1 μg of genomic DNA that was sheared in a gTube (Covaris, Woburn, MA, United States) for 1 min at 6,000 r/min in a MiniSpin plus microcentrifuge (Eppendorf, Hauppauge, NY, United States). The sheared DNA was converted to a shotgun library with the LSK-108 kit (Oxford Nanopore) with the Expansion barcoding kit (EXP-NBD103). The libraries were pooled in equimolar concentration and the pool was sequenced on two SpotON MK I (R9.5) flowcells for 48 h using a MinION MK 1B sequencer. Basecalling was done with software MinKNOW version 1.7.7 and demultiplexing was performed with the Albacore software version 1.2.4. Sixty bp were trimmed from each end of the reads to remove barcodes using a custom script.

Detailed assembly steps are outlined in **Supplemental Files S1****–****S4** for the four genomes. In brief, trimming was performed using a simple Perl script to remove 60 nt from the ends of the reads, retaining reads longer than or equal to 1000 nt for the final assembly. Canu v1.5 (Koren et al., 2017) was used for assembly with the following parameters: “canu -p asm -d useGrid = false -nanopore-raw genomeSize=<GENOME_SIZE> <TRIMMED_FASTQ>”, using the relevant quality-trimmed reads from Porechop and the following estimated genome sizes as input: *S. stipitis* = 15.4 Mb, *L. elongisporus* = 15.5 Mb, *M. guilliermondii* = 10.6 Mb, *S. passalidarum* = 13.2 Mb.

Trimmed Oxford Nanopore reads were then aligned against their associated assembly using bwa v0.7.5 (Li, 2018) by first indexing the assembly using “bwa index <ASSEMBLY>”, then aligning reads using the parameters “bwa mem -x ont2d <ASSEMBLY_INDEX> <TRIMMED_READS>”. The alignment was then used to polish the assembly using nanopolish v0.7.1 (Senol Cali et al., 2018). Quality-trimmed MiSeq data were used to further polish the assembly using Pilon v1.22 for error correction (Walker et al., 2014). Assembly names and GenBank accession numbers for the genome sequences for *L. elongisporus* NRRL YB-4239, *M. guilliermondii* ATCC 6260, *S. passalidarum* NRRL Y-27907, and *S. stipitis* CBS 6054 are located in **Table 1**.

### Identification of *ALS* Genes and Predicted Als Protein Features

Methods for identifying *ALS* genes and deducing predicted protein features were nearly identical to those reported in previous publications (Oh et al., 2019; Oh et al., 2021). Details were reproduced here for the reader’s convenience. BLAST (https://blast.ncbi.nlm.nih.gov/Blast.cgi) was used to identify potential *ALS* genes and Als proteins in the genome sequences (**Table 1**). BLAST searches were also conducted using the *Candida* Genome Database (www.candidagenome.org; Skrzypek et al., 2017). Query sequences included all *C. albicans ALS* genes as reported by Oh et al. (2019). As new *ALS*/Als sequences were identified, they were also used as BLAST queries until search reports failed to reveal new sequences.

*ALS* gene sequences were verified or corrected by Sanger sequencing of PCR-amplified products. Primers were designed using the Primer Quest Tool (https://www.idtdna.com) and primers purchased from Integrated DNA Technologies (Coralville, IA). PCR used Q5 High Fidelity DNA Polymerase (New England Biolabs) according to the manufacturer’s instructions. Extension time was adjusted to promote amplification of the desired product size. PCR products were purified using the MultiScreen HTS 96-well Filtration System (Millipore), then Sanger sequenced at the Roy J. Carver Biotechnology Center (University of Illinois at Urbana-Champaign). A list of primer sequences is located in **Supplementary Table S1**.

SignalP-5.0 Server (http://www.cbs.dtu.dk/services/SignalP; Nielsen, 2017) was used to locate putative secretory signal peptides. The Big-PI Fungal Predictor (https://mendel.imp.ac.at/gpi/fungi_server.html; Eisenhaber et al., 2004) identified potential GPI anchor addition sites. Dotmatcher (http://emboss.bioinformatics.nl/cgi-bin/emboss/dotmatcher) was used to detect repeated sequences. The ExPASy Server was used to assess amino acid sequence composition (https://web.expasy.org/protparam; Gasteiger et al., 2005). European Bioinofrmatics Institute (EMBL-EBI) tools were used for translating nucleotide sequences, sequence alignment, and other general processes (https://www.ebi.ac.uk/services; Cook et al., 2017). β-aggregation potential in amino acid sequences was assessed using Tango (http://tango.crg.es; Fernandez-Escamilla et al., 2004; Rousseau et al., 2006). All nucleotide sequences were translated with the alternative yeast nuclear code (translation table 12) except those from *C. glabrata* and *S. cerevisiae* which used the standard code (translation table 1). There was disagreement about the translation table for *L. elongisporus*: the NCBI database used translation table 1 even though the species was recognized as part of the CUG clade that translates CUG as Ser instead of Leu (translation table 12; Santos et al., 2011).

### Phylogenetics Analysis

The kingdom Fungi phylogeny of Li et al. (2021), based on 290 genes from 1644 species, was pruned using the R package ape (Paradis and Schliep, 2019). *C. metapsilosis* was not included in the phylogeny. Because of its close relationship to *C. parapsilosis* and *C. orthopsilosis* (Tavanti et al., 2005), *C. metapsilosis* was attached at the root of these sister species.

Sequences for phylogenetic analysis were trimmed to include only the NT-Als domain, which has adhesive function (Lin et al., 2014). Secretory signal peptide sequences were included in the analysis. Tango (http://tango.crg.es; Rousseau et al., 2006; Fernandez-Escamilla et al., 2004) was used to define the amyloid-forming region (AFR) that marks the C-terminal end of NT-Als (Lin et al., 2014). Sequences that did not encode an AFR were trimmed to a length similar to others in the dataset. Amino acid sequences used for phylogenetic analysis were compiled into **Supplementary File S5**. Sequences were aligned using MAFFT v7.453 (Katoh and Standley, 2013). A phylogeny was inferred using the best fit model WAG+G+I and subsequent bootstrap analysis was performed using RAxML-NG (Kozlov et al., 2019).

### Structural Predictions

Protein structural predictions were derived using AlphaFold (Jumper et al., 2021), accessed using AlphaFold Colab (https://colab.research.google.com/github/deepmind/alphafold/blob/main/notebooks/AlphaFold.ipynb). ColabFold (Mirdita et al., 2021; https://colab.research.google.com/github/sokrypton/ColabFold/blob/main/AlphaFold2.ipynb) was used for more-rapid generation of structural prototypes. ColabFold utilized MMSeqs2 (UniRef+Environmental), as well as a custom MSA file constructed using Hhblits Toolkit server (Zimmerman et al., 2018; Gabler et al., 2020).

Clustal Omega (Madeira et al., 2019) was used to align amino acid sequences from **Supplementary File S5** with those from 4LE8, the Protein Data Bank (https://www.rcsb.org) entry for the crystallographic structure of *C. albicans* NT-Als3 (chain A, 299 amino acids). This molecule did not include the secretory signal peptide at the N-terminal end nor the amyloid-forming region at the C-terminal end. Sequences in **Supplementary File S5** were trimmed according to the Clustal Omega alignment and the trimmed sequences were used for AlphaFold analysis. Sequences for other fungal cell-surface proteins were included as controls in the structural analysis: *C. albicans* Hyr1 (NCBI Reference Sequence: XP_722183.2, amino acids 21 to 309) and *S. cerevisiae* Flo1 (NCBI Reference Sequence: NP_009424.1; amino acids 25 to 273). Structural visualization and alignments used the PyMOL Molecular Graphics System, Version 2.5.2 (Schrödinger, LLC).

## Results

### Detection and Assembly of *ALS* Sequences in Eight Fungal Species

**Figure 1:**
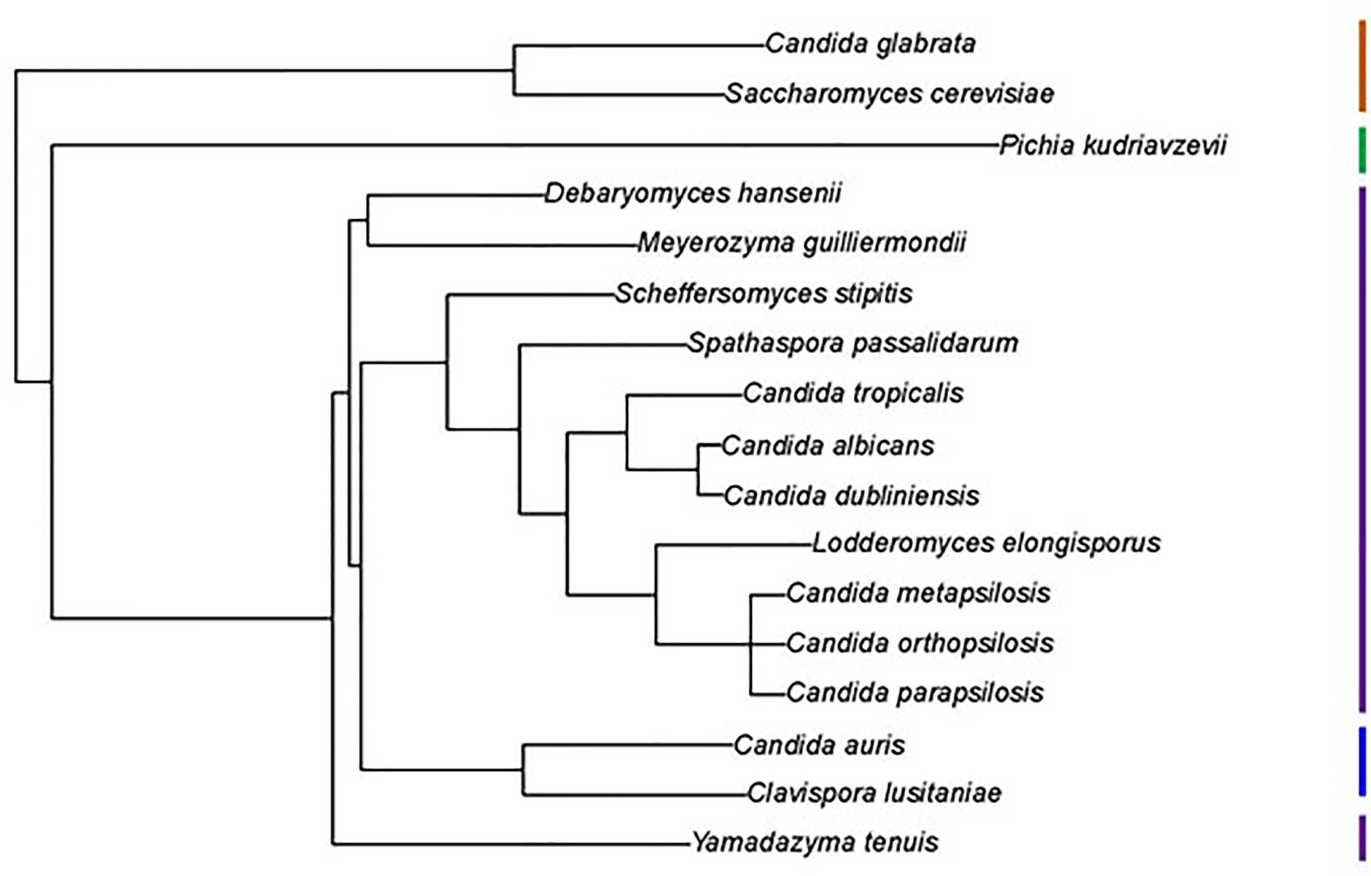
Phylogenetic tree showing relationships between fungal species used in this study. The tree was pruned from the genome-scale phylogeny of the kingdom Fungi developed by Li et al. (2021). The phylogeny was based on 290 concatenated sequences in 1644 species. *C. metapsilosis* was not part of the original analysis and was added to the tree based on its close relationship with *C. parapsilosis* and *C. orthopsilosis* (Tavanti et al., 2005). All species listed were Phylum Ascomycota, Subphylum Saccharomycotina, Class Saccharomycetes, Order Saccharomycetales. Vertical bars on the right of the image indicate Family designations according to the NCBI Taxonomy Database (https://www.ncbi.nlm.nih.gov/taxonomy). Brown = Family *Saccharomycetaceae*, Green = Family *Pichiaceae*, Purple = Family *Debaryomycetaceae*, and Blue = Family *Metschnikowiaceae*.

Examination of new and existing genome sequence data (**Table 1**) provided the information needed to locate, assemble and/or validate the sequence for 47 *ALS* loci (5 from *Lodderomyces elongisporus* NRRL YB-4239; 3 from *Candida auris* B8441; 3 from *Scheffersomyces stipitis* CBS 6054; 4 from *Meyerozyma guilliermondii* ATCC 6260; 29 from *Spathaspora passalidarum* NRRL Y-27907; and 1 each from *Clavispora lusitaniae* ATCC 42720, *Yamadazyma tenuis* ATCC 10573, and *Debaryomyces hansenii* CBS 767). These sequences are presented here in the context of previously described *ALS* loci including 8 from *Candida albicans* SC5314 (Hoyer et al., 2008), 7 from *Candida dubliniensis* CD36 (Jackson et al., 2009), 13 from *Candida tropicalis* MYA-3404 (Oh et al., 2021), 5 from *Candida parapsilosis* CDC 317 (Oh et al., 2019), 3 from *Candida orthopsilosis* Co 90-125 (Lombardi et al., 2019), and 4 from *Candida metapsilosis* ATCC 96143 (Oh et al., 2019). **Supplementary Table S2** details all gene names, GenBank accession numbers, and predicted protein characteristics. Gene names were taken from locus tags associated with the annotated reference genome for each species. For genes that were not annotated in the reference genome, “0.5” was added to the name, indicating that the ORF was located between two previously annotated genes. **Figure 1** displays the phylogenetic relationship between the species described here.

#### L. elongisporus

*L. elongisporus* is an infrequent human pathogen and, as accurately portrayed in **Figure 1**, closely related to *C. parapsilosis* (Lockhart et al., 2008). Two *L. elongisporus* genome sequences were available through the National Center for Biotechnology Information (NCBI; https://www.ncbi.nlm.nih.gov) database; both were for strain NRRL YB-4239 (**Table 1**). Assembly ASM14968v1 was comprised of 145 contigs arranged into 28 scaffolds (i.e. composed of contigs and gaps that are indicated by insertion of “NNN”) while ASM1362098v1 had 53 contigs. *ALS* gene names were derived from the original annotation of the reference genome (ASM14968v1). Although a *L. elongisporus* karyotype was not located in the literature, the number of contigs/scaffolds was far higher than an expected number of chromosomes, indicating that the genome assemblies required refinement.

**Figure 2:**
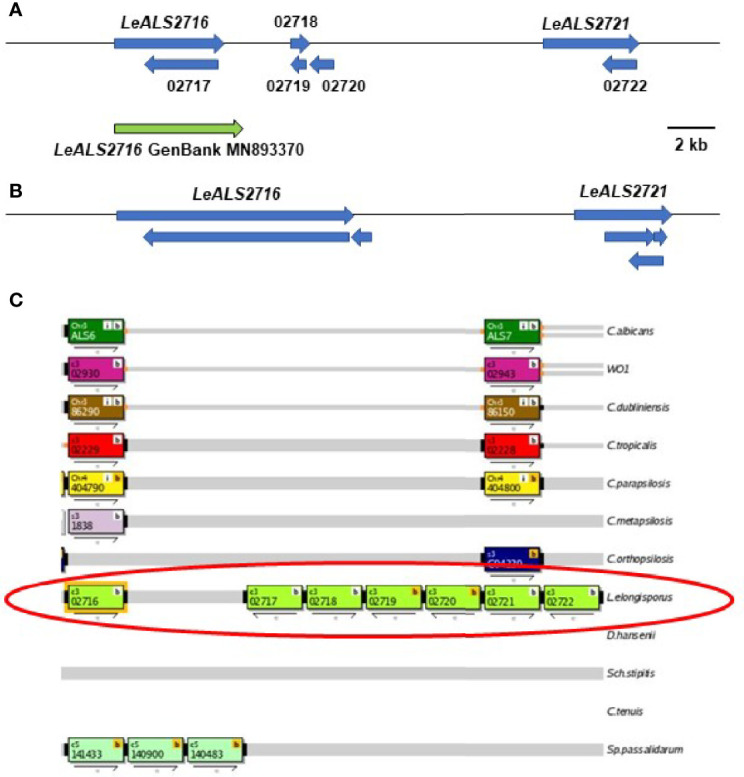
Schematic of the genome region that included *LeALS2716* and *LeALS2721* from **(A)** assembly ASM14968v1 and **(B)** assembly ASM1362098v1. **(C)** shows the region as represented in the *Candida* Genome Order Browser (CGOB; https://cgob.ucd.ie; Maguire et al., 2013) based on ASM14968v1 data. *L. elongisporus* information is circled in red; ORF numbers are shown in each rectangle and the direction of transcription indicated by the arrow below. ORFs 02718, 02719, and 02720 featured the *IFF/HYR* repeated sequences that were also found in *LeALS2716* suggesting that ORF 02716 was longer than initially annotated. The large number of repeated sequences complicated genome sequence assembly in this region. The predicted size of *LeALS2716* in ASM1362098v1 was greater than the final PCR-amplified/Sanger-sequenced fragment that was deposited into GenBank (accession number MN893370; green arrow).

BLAST analysis using *ALS* query sequences consistently revealed 6 *L. elongisporus* loci (**Supplementary File S6**). *LeALS734* was located in the middle of a > 3 Mb supercontig in each genome assembly. *LeALS2536*, *LeALS2716* and *LeALS2721* were located in the center of another supercontig in ASM14968v1 with the latter two genes located near each other and transcribed in the same direction (**Figure 2**). The remaining genes (*LELG_04272*, *LeALS5708*) were each on their own supercontig.

Proteins predicted from five loci (*LeALS734*, *LeALS2536*, *LeALS2716*, *LeALS2721*, and *LeALS5708*) had features expected for an Als protein including a secretory signal peptide, NT-Als domain, Ser/Thr-rich sequences, and GPI anchor addition site (**Supplementary Table S2**). Although the protein translated from the sixth locus (*LELG_04272*) had tandemly repeated sequences like those in Als proteins, the ORF did not predict an NT-Als domain (**Supplementary File S6**). BLAST of sequences N-terminal to the LELG_04272 tandem repeats best matched *M. guilliermondii* PGUG_02520, a 660-amino acid protein of unknown function. The hybrid *LELG_04272* was likely the result of recombination between an *ALS* open reading frame (ORF) and another gene. Such recombination was observed previously between *ALS* and *IFF/HYR* genes in *C. metapsilosis CmALS2265* which encoded an Als N-terminal domain on the stalk of an Iff/Hyr protein (Oh et al., 2019). Because LELG_04272 lacked an NT-Als adhesive domain, it was not given an Als family name.

Similar to *C. metapsilosis*, recombination between *ALS* and *IFF/HYR* genes was evident in *L. elongisporus* (**Supplementary File S6**). *LeALS734* and *LeALS2716* each had the 5’ end of an *ALS* gene and the 3’ end of an *IFF/HYR* gene. Consensus tandem repeat sequences for each locus closely matched that for *CmALS2265* (Oh et al., 2019). The remaining *LeALS* genes did not include a central domain of tandemly repeated sequences.

The predicted amino acid sequence of the NT-Als domain was highly conserved between LeAls2536, LeAls2721, and LeAls5708 (70-80% identity; **Supplementary File S6**). LeAls734 and LeAls2716 were approximately 40% identical to each other and to the other *L. elogisporus* Als proteins. Since the crystallographic structure of *C. albicans* NT-Als3 is known (Lin et al., 2014), the CaAls3 sequence was included for comparison to newly characterized proteins. The LeAls proteins were 33-45% identical to the CaAls3 NT-Als sequence.

The incomplete *L. elongisporus* genome assemblies provided multiple opportunities for repairing ORFs. LELG_04272 lacked a GPI anchor addition site in its original annotation in ASM14968v1 (**Supplementary File S6**). Primer design, PCR amplification, and Sanger DNA sequencing indicated that the downstream *LELG_04271* was actually the 3’ end of the *LELG_04272* coding region. The full sequence was deposited into GenBank (accession number OK172378). The region encoding *LeALS2716* and *LeALS2721* was also assembled poorly in the available genome sequences (**Figure 2**). Insertion of many NNN (designating unknown sequence) in this region of assembly ASM14968v1 made it difficult to locate the gene boundaries. The size of the verified *LeALS2716* was larger than predicted in assembly ASM14968v1, but smaller than suggested in assembly ASM1362098v1. The difference was possibly attributable to variation in tandem repeat copy number between alleles in the diploid *L. elongisporus*. A similar scenario was encountered for completing the sequence of *LeALS734*: several previously designated ORFs were combined into the final *LeALS734*, yet the sequence deposited into GenBank was shorter than anticipated from the genome assemblies that guided PCR primer design (details not shown).

#### M. guilliermondii

In addition to its role as a human pathogen, *M. guilliermondii* has applications in bioprocessing (e.g. production of riboflavin and xylitol) and bioremediation (reviewed in Papon et al., 2013). Eight *M. guilliermondii* genome assemblies were reported in the NCBI database. Two sequences were available for strain ATCC 6260, which was the focus of this work. ASM14942v1 consisted of 71 contigs arranged on 9 scaffolds. ASM694215v1 had 9 contigs.

Two of the 4 *ALS* loci in strain ATCC 6260 (*MgALS2302*, *MgALS3259*) were on the same chromosome, approximately 1.7 Mb away from each other at opposite telomeres, transcribed in opposite directions (**Supplementary File S7**). The 3’ end of the 9681-bp *MgALS2302* was located approximately 10 kb from one telomere while the 3’ end of the 6963-bp *MgAls3259* was about 7 kb from the opposite telomere. The other two *MgALS* loci (*MgALS673* and *MgALS3330*) were each on other large contigs, likely representing *M. guilliermondii* chromosomes.

The coding region for *MgALS3259* predicted two potential start sites, one 60 bp upstream from the other. The second predicted Met start site provided a clear secretory signal peptide and was included in GenBank accession MH753514. *PGUG_03259* (accession number XM_001485480) included the upstream start site instead. Another difference between sequences deduced here and those from the ASM14942v1 assembly included the length of *MgALS2302*. The gene was considerably longer in the current work compared to previous GenBank deposits (9681 bp vs. 3840 bp). The new gene name reflected its inclusion of multiple ORFs (*PGUG_02299*, *PGUG_02300*, *PGUG_02301*, and *PGUG_02302*) and start of the gene in *PGUG_02302*.

Three of the 4 *MgALS* loci had an extensive central region of tandemly repeated sequences that were very similar to consensus repeats from previously characterized *Candida* genes (**Supplementary File S7**). The most-common repeat unit size was 35 amino acids with a range of 34-36 amino acids. The predicted MgAls2302 and MgAls3259 NT-Als protein sequences were the most similar to each other. They shared 57% identity while other NT-Als comparisons were only about 30% identical.

#### C. auris

Availability of genome sequences for over 100 isolates of *C. auris* in the NCBI database reflected its emergence as an important pathogen (Du et al., 2020). Strain B8441 (18 contigs arranged into 15 scaffolds; assembly Cand_auris_B8441_V2) was used for this work (**Table 1**). The three *ALS* loci in strain B8441 were on different scaffolds with *CauALS2582* in a subtelomeric location (**Supplementary File S8**). Only one predicted *C. auris* Als protein (CauAls4112) had a central domain of tandemly repeated (34-39 amino acids) sequences. *C. auris* NT-Als sequences were 38-50% identical to each other and 25-33% identical to *C. albicans* NT-Als3. BLAST searching of other *C. auris* genomes showed that many were missing the *CauALS2582* locus. These observations matched the report of Muñoz et al. (2021) that described loss of subtelomeric adhesins such as *CauALS2582* in clade II *C. auris* isolates.

#### S. stipitis

*S. stipitis* is studied because of its ability to ferment xylose, and thus its potential to contribute to biofuel production from hemicellulose (Jeffries et al., 2007). Because *S. stipitis* is within the CUG clade of the Family *Debaryomycetaceae*, the genome of this beetle-associated commensal yeast is often included in comparisons that feature human-pathogenic species (Wohlbach et al., 2011; Maguire et al., 2013). The NCBI database included three *S. stipitis* genome sequences: two for strain CBS 6054 (ASM20916v1 = 9 contigs arranged on 8 scaffolds; ASM694211v1 = 30 contigs) and one for strain NRRL Y-7124. Efforts here focused on strain CBS 6054 (**Table 1**).

Strain CBS 6054 had three *ALS* loci (**Supplementary Table S2**). Two (*SsALS2386* and *SsALS2786*) were on chromosome 2, approximately 700 kb apart and transcribed in opposite directions (**Supplementary File S9**). The third (*SsALS4579*) was on chromosome 4. NT domains of the predicted chromosome 2 proteins were 58% identical. However, each was only 27-29% identical to SsAls4579. Both SsAls2386 and SsAls2786 had a central domain of tandemly repeated sequences that resembled those from previously studied *Candida* species.

BLAST searches and text searches of the NCBI database revealed other repeat-containing loci that were annotated as *ALS* genes in assembly ASM20916v1. For example, *PICST_4391* (called “*ALS1*”) was a fragment of *SsALS2786* described above. *PICST_4539* (called “*ALS1.2*”) was a fragment of *SsALS2386* and *PICST_31759* (called “*ALS6*”) corresponded to *SsALS4579*. *PICST_31095*, despite being named “*ALS2*”, belonged to the *IFF/HYR* family. Another, called “*ALS4*” (*PICST_30411*), was comprised only of repeated sequences. It was flanked by sequences that contained many stop codons with a low likelihood of encoding a larger ORF. In assembly ASM20916v1, *PICST_30411* was adjacent to an *IFF/HYR* gene in a subteromeric region on chromosome 2.

#### S. passalidarum

The *S. passalidarum* genome was also sequenced to study xylose metabolism in fungal species (Wohlbach et al., 2011). Because initial BLAST searching revealed an unusually high number of *ALS* loci in *S. passalidarum*, additional analysis was pursued. Two genome assemblies for strain NRRL Y-27907 were located in the NCBI database (**Table 1**). One was created by the DOE Joint Genome Institute (Spathaspora passalidarum v2.0; 26 contigs arranged onto 8 scaffolds) and the other was part of the current work (ASM1362096v1; 10 contigs).

BLAST searching revealed 29 physical loci with sequence similarity to *ALS* genes (**Supplementary Table S2**). Seven of the 8 contigs from assembly Spathaspora passalidarum v2.0 had at least 1 *ALS* locus, and as many as 7 *ALS* loci (**Table 2**). Four locations featured *ALS* genes adjacent to each other: *SpALS64434* and *SpALS64435* on scaffold 1, *SpALS50348* and *SpALS50349* on scaffold 3, *SpALS153035* and *SpALS153035.5* on scaffold 4, and *SpALS68952* and *SpALS68952.5* on scaffold 8. Two *ALS* genes were subtelomeric: *SpALS131476* on scaffold 1 and *SpALS137089* on scaffold 3.

**Table 2:** Physical location of *ALS* loci in the *S. passalidarum* NRRL Y-27907 genome.

Scaffold (Size, Mb)	Gene Name	Approx. Location (nt)*	Tsc^†^	Scaffold (Size, Mb)	Gene Name	Approx. Location (nt)	Tsc^†^
1 (2.64)	*SpALS131476*	9400	R^‡^	4 (1.81)	*SpALS61022.5*	69000	R
	***SpALS64434***	**1407000**	**F**		*SpALS152224*	180000	R
	***SpALS64435***	**1411000**	**F**		***SpALS153035***	**876400**	**F**
	*SpALS146555*	1607000	F		***SpALS153035.5***	**879600**	**F**
					*SpALS138016*	1122200	F
2 (2.12)	*SpALS134590*	189000	R				
	*SpALS134590.5*	254000	F	5 (1.65)	*SpALS155003*	1011000	R
	*SpALS134426*	337300	F		*SpALS140483*	1300300	R
	*SpALS134874*	827000	R		*SpALS140900*	1306000	R
	*SpALS135549*	835000	R		*SpALS141433*	1312800	R
	*SpALS59511.5*	940000	R				
				6 (1.18)	*SpALS156463*	710700	F
3 (2.07)	*SpALS137089*	8400	R^‡^				
	*SpALS49824*	486000	R	8 (0.85)	***SpALS68952***	**600000**	**R**
	*SpALS136382*	858000	F		***SpALS68952.5***	**604000**	**R**
	*SpALS55077*	1015000	F				
	*SpALS66147*	1021200	R				
	***SpALS50348***	**1700000**	**F**				
	***SpALS50349***	**1702000**	**F**				

*Approximate location of each ORF in the Spathaspora passalidarum v2.0 assembly (GCA_000223485.1). Pairs of contiguous ORFs are indicated in bold type.

^†^Direction of transcription is noted as F (forward) or R (reverse).

^‡^Subtelomeric localization.

Examination of the encoded protein sequences showed that many did not have repeated sequences in the center (**Supplementary File S10**). When present, repeated sequences were unusually long (**Supplementary Table S2**), highly variable in the length of the repeat unit, and did not necessarily have tandemly arrayed repeat copies. A consensus of the 80-amino-acid and 89-amino-acid versions of the *S. passalidarum* repeated sequence resembled the T (Thr-rich) domain of *C. albicans* Als proteins that is located between the end of NT-Als and the start of the tandem repeat region (reviewed in Hoyer and Cota, 2016). Some manuscripts depict this region separately from “NT-Als” for which the crystal structure is known (e.g. Oh et al., 2019). Other manuscripts, like this one, include the T domain in the “NT-Als +” region for each protein.

*S. passalidarum* had several partial *ALS* genes (**Supplementary File S10**). In previous analyses, partial *ALS* genes indicated poor genome assembly that could be corrected by PCR amplification and Sanger sequencing (Oh et al., 2021). Here, however, PCR amplification and Sanger sequencing validated the accuracy of the partial *ALS* genes including the contiguous loci *SpALS50348* and *SpALS50349* that each only encoded an NT-Als domain with a secretory signal peptide. A clear stop codon between *SpALS68952.5* and *SpALS68952* created pseudogenes from a perhaps previously long, functional gene. Some predicted proteins lacked features such as a secretory signal peptide that would direct the protein toward cell-surface localization. Signal-peptide-encoding sequences were noted upstream of *SpALS66147* and *SpALS134590.5* but were separated from the ORF by at least one clear stop codon. Other predicted proteins (SpAls49824, SpAls61022.5, SpAls140483, and SpAls140900; **Supplementary File S10**) showed only a weak signal for GPI anchor addition, suggesting that the mature proteins may not be localized to the cell surface. Among the proteins predicted to have both secretory signal peptide and strong GPI anchor addition signal, many were short, raising the question of how far the adhesive NT-Als domain could be projected away from the *S. passalidarum* cell surface to contact binding partners.

Comparison between the NT-Als domains for the predicted *S. passalidarum* proteins showed considerable sequence divergence from *C. albicans* NT-Als3 and from each other (**Supplementary File S10**). Many comparisons were in the range of 22-45% identity although greater sequence conservation was observed for some pairs (60-80% range). Contiguous loci (37-62% identity) were not necessarily more conserved than other comparisons. The most-conserved pair was SpAls55077 and SpAls134590 (89%) potentially indicating a gene duplication.

#### Y. tenuis

Interest in the *Y. tenuis* genome also arose from its xylose fermentation capabilities (Wohlbach et al., 2011). A single complete *ALS* locus was detected in the only *Y. tenuis* genome assembly available in the NCBI database (strain ATCC 10573; Candida tenuis v1.0; **Table 1**). The predicted YtAls93631 protein was > 3000 amino acids and included a central domain of tandemly repeated sequences (32-45 amino acids; **Supplementary File S11**). The most common repeat unit was 35 amino acids. The *Y. tenuis* genome sequence was quite fragmented, consisting of 74 contigs arranged onto 25 scaffolds. A second potential *ALS* locus was detected at the end of a scaffold sequence (CANTEscaffold_00010; 12.1 kb total length) suggesting that other *Y. tenuis ALS* loci may be revealed as the genome assembly matures.

#### C. lusitaniae

*C. lusitaniae* is a less-frequent cause of human candidiasis (reviewed in François et al., 2001). Of the species considered here, *C. lusitaniae* was the most closely related to *C. auris* (**Figure 1**). More than 30 C*. lusitaniae* genome assemblies were available in the NCBI database. Observations here focused on strain ATCC 42720 (ASM383v1) which was comprised of 88 contigs arranged onto 9 scaffolds. One long *ALS* gene (> 8 kb) was detected in this sequence (**Supplementary Table S2**). BLAST of other available genomes also suggested a single *ALS* locus, although incomplete genome assemblies with many contigs may obscure the presence of other loci. Two different types of repeated DNA sequences were found in the center of *ClALS3274* (**Supplementary File S11**). BLAST searching of the non-redundant nucleotide database suggested that the repeats predicting a 33 amino acid sequence were unique. The other repeated sequences were primarily 67 amino acids in length, although some repeat units expanded to 102 amino acids. Sequences within these longer repeats were reminiscent of motifs found in the Als consensus repeat from *C. parapilsosis/C.orthopsilosis/C. metapsilosis*, as well as the 80-amino-acid consensus repeat from *S. passalidarum* Als proteins (**Supplementary File S10**).

#### D. hansenii

Rather than being studied as a pathogen, *D. hansenii* is discussed commonly in the context of food production (summarized in Gori et al., 2011). *D. hansenii* growth requires surface adhesion (Mortensen et al., 2005), a property that might involve Als proteins. The NCBI database displayed 12 *D. hansenii* genome sequences, representing 11 different strains. This work focused on CBS 767 (ASM644v2), which was designated as the reference genome and had 15 contigs arranged on 8 scaffolds. A single *ALS* locus was detected, located on the chromosome G contig (**Supplementary Table S2**). The predicted protein, DhAls2178, did not contain tandemly repeated sequences (**Supplementary File S11**).

#### C. glabrata

*C. glabrata* is well-recognized as a human pathogen, particularly for its ability to rapidly evolve resistance to antifungal drugs (Vale-Silva and Sanglard, 2015). Eighteen *C. glabrata* genomes were present in the NCBI database. BLAST of the strain CBS 138 sequence using *ALS* and Als queries revealed CAGL0G04125g, which was annotated to indicate similarity to *S. cerevisiae* Sag1 (**Supplementary File S11**). Like Sag1, the *C. glabrata* predicted protein did not encode a central domain of tandemly repeated sequences.

### NT-Als Structural Predictions

The *C. albicans* NT-Als3 structure was solved crystallographically (Lin et al., 2014; Protein Data Bank accession number 4LE8), providing experimental information that can be used to produce structural predictions for other NT-Als proteins. A crystallographic structure has not been reported for *S. cerevisiae* Sag1. The recent development of the highly accurate protein structural prediction program AlphaFold (Jumper et al., 2021) and widespread access to its use (e.g. Mirdita et al., 2021) provided the opportunity to assess predicted structural relatedness between the diverse set of NT-Als sequences and their similarity to Sag1.

**Figure 3:**
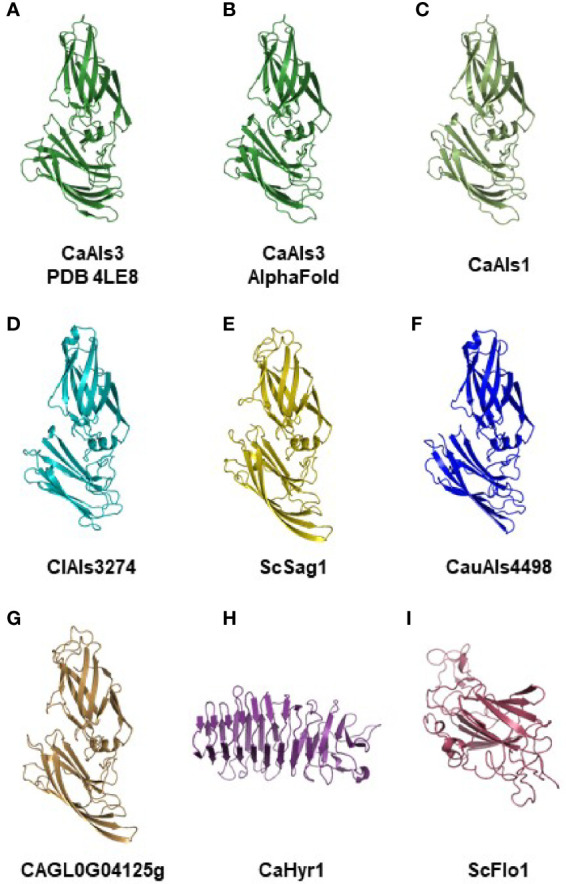
Experimental and AlphaFold-predicted protein structures. **(A)** Crystallographic structure of *C. albicans* NT-Als3 (Protein Data Bank accession 4LE8) visualized using PyMOL. **(B)** *C. albicans* NT-Als3 structure predicted by AlphaFold from the 4LE8 amino acid sequence. AlphaFold structural predictions for the corresponding region of *C. albicans* NT-Als1 (**C**; 83% identical to NT-Als3), ClAls3274 (**D**; 33% identical to NT-Als3), ScSag1 (**E**; 25% identity), CauAls4498 (**F**; 31% identity), and CAGL0G04125g (**G**; 21% identity). An AlphaFold structural prediction was also completed for the N-terminal functional domain of *C. albicans* Hyr1 **(H)** and *S. cerevisiae* Flo1 **(I)** to demonstrate structural diversity among cell-surface proteins that contain a central domain of repeated sequences. The AlphaFold prediction for *C. albicans* NT-Als3 **(B)** recapitulated the known experimental structure (**A**; RMSD = 0.53 as calculated using PyMOL align). Predictions for molecules **(B–G)** produced the same general structure suggesting that all should be included in the Als protein family. **Supplementary File S12** shows the structures of *C. albicans* NT-Als3 **(B)** and ScSag1 **(E)** aligned with the disulfide bonds highlighted.

**Figure 3** shows the crystallographic structure of *C. albicans* NT-Als3 from 4LE8 (**Figure 3A**) and the AlphaFold-predicted structure for the same amino acid sequence (**Figure 3B**). The root-mean-square-deviation (RMSD) value for alignment of the two structures was 0.53, indicating the ability of AlphaFold to reproduce the crystallographic structure of NT-Als3 from its amino acid sequence. AlphaFold was then used to predict the structures of other NT-Als proteins, particularly those with limited sequence identity to *C. albicans* NT-Als3 (**Figures 3C–G**). Strikingly, a similar structure was predicted for each protein, including Sag1. Analysis of each NT-Als sequence in **Supplementary File S5** produced the same general protein shape (data not shown) suggesting that all, including Sag1, were unified as a family. To demonstrate diversity in structural predictions, the amino acid sequences for the N-terminal domain of the cell-surface proteins *C. albicans* Hyr1 (amino acids 21 to 309) and *S. cerevisiae* Flo1 (amino acids 25 to 273) were entered into AlphaFold. The resulting structural predictions were different from the Als family and from each other (**Figures 3H, I****)**.

### Adhesive Activity Predictions From NT-Als Protein Features

**Figure 4:**
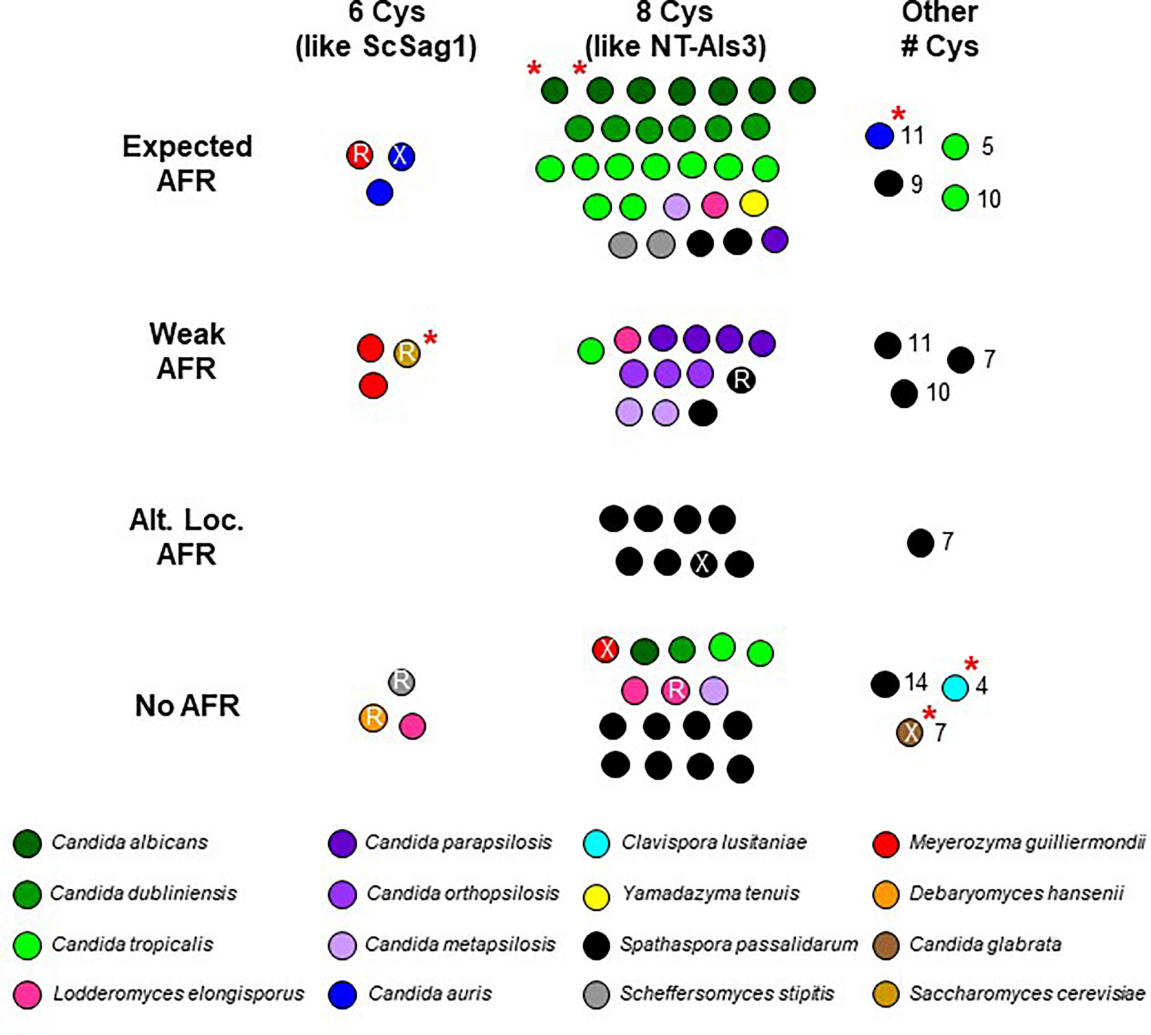
Visualization of NT-Als sequence features from **Supplementary Table S2**. Each spot represents an Als protein; color coding matches **Supplementary Table S2**. *C. albicans* NT-Als3 has 8 Cys that create four disulfide bonds (Lin et al., 2014) while *S. cerevisiae* Sag1 has only 6 and is missing the C57-C133 disulfide bond that is present in NT-Als3 (Salgado et al., 2011). Most NT-Als proteins had 8 Cys like NT-Als3 (center column), some had 6 Cys like ScSag1 (left column), and others had varying numbers of Cys (range of 4 to 14; right column). Presence of the amyloid-forming region (AFR) in *C. albicans* Als proteins promotes protein aggregation (Ho et al., 2019). While many Als proteins had the expected AFR (strength and location; top row), others had a predicted weak AFR (second row) or none (bottom row). Some *S. passalidarum* Als proteins had a strong AFR 20-30 amino acids C-terminal to the expected location known in *C. albicans* (third row). It was unknown whether this alternative location contributed to aggregative potential. An invariant Lys in *C. albicans* NT-Als establishes a salt bridge with the C-terminal carboxylic acid of an incoming peptide ligand (Salgado et al., 2011). Arg (R) in this location may serve a similar function. Some predicted proteins did not have a positively charged amino acid in this position (X). A red asterisk indicates proteins featured in the structural predictions (**Figure 3**).

In order to provide biologically meaningful adhesive activity on the fungal cell surface, Als proteins need a secretory signal peptide and GPI anchor addition site to achieve an effective localization (Lu et al., 1994). The crystallographic structure of *C. albicans* NT-Als3 (Lin et al., 2014) demonstrated the involvement of 8 Cys residues in folding of the mature protein and the function of an invariant Lys at the end of the binding cavity that sinks the negative charge of incoming peptide ligands. Other work demonstrated the importance of the amyloid-forming region (AFR) for increasing the avidity of aggregative interactions (Ho et al., 2019). **Supplementary Table S2** evaluated each of these features for the previously characterized and newly introduced Als proteins. The data were summarized in **Figure 4** from which various hypotheses emerged.

The cluster of dots in the top center of **Figure 4** contained proteins with 8 Cys and an invariant Lys. Each protein represented in the top center cluster of dots also had an AFR with high predicted β-aggregation potential and a location immediately C-terminal to the NT-Als domain as in *C. albicans* Als proteins (Salgado et al., 2011; Lipke et al., 2018). The top center cluster of dots included 7 of the 8 C*. albicans* Als proteins, 6 of 7 from *C. dubliniensis*, and 9 of 13 from *C. tropicalis* suggesting robust potential for functional adhesive interactions by these species. Also present in the top center cluster of dots were 2 of the *S. stipitis* Als proteins, 2 from *S. passalidarum*, and 1 each from *C. parapsilosis*, *C. metapsilosis*, *L. elongisporus*, and *Y. tenuis*.

Viewing potential Als function according to these criteria provided predictions about the adhesive activity in other species. For example, *C. parapsilosis*, *C. orthopsilosis*, and *C. metapsilosis* Als proteins almost universally had a weaker or absent AFR leading to the hypothesis that these species are functionally less adherent than *C. albicans*, *C. dubliniensis*, and *C. tropicalis*. Data in **Figure 4** suggested a similar hypothesis for *S. passalidarum*: despite its overabundance of *ALS*-like loci, *S. passalidarum* encoded only two proteins (SpAls138016 and SpAls156463; **Supplementary File S10**) that possessed the functional features present in NT-Als3 and therefore may not be a very adherent species. However, nine SpAls proteins had AFR sequences 20-30 amino acids C-terminal to the location where they are found in *C. albicans* Als3 (**Supplementary Table S2**). Experimentation is required to evaluate whether sequences with high β-aggregation potential in this alternative location contribute to *S. passalidarum* aggregative interactions. Using the criteria in **Figure 4**, *C. lusitaniae* was an outlier. Its sole Als protein, ClAls3274, had only 4 Cys residues and no AFR. Despite these key differences, the predicted structure for the NT-ClAls3274 domain was similar to those from other species (**Figure 3D**). The contribution of ClAls3274 remains to be tested, as does the overall adhesion profile for the species.

Proteins with features that more closely resemble Sag1 than *C. albicans* Als3 segregated to the left column of the diagram. Substitution of Arg (R printed in the dot) in place of the invariant Lys was a common theme. Three of the four *M. guilliermondii* Als proteins were in the left column. Perhaps these proteins have more-specific, higher-affinity adhesive interactions that more-closely resemble those of Sag1, rather than NT-Als3. The same prediction could be made for two of the *C. auris* proteins, as well as the sole protein from *D. hansenii*.

Information presented in **Figure 4** and **Supplementary Table S2** showed that some species had a complement of Als proteins that possessed features of both *S. cerevisiae* Sag1 and *C. albicans* Als3. For example, *S. stipitis* SsAls4579 had Sag1-like features (lower left, **Figure 4**) while SsAls2386 and SsAls2786 looked more like *C. albicans* Als3 (top center, **Figure 4**). The line diagrams in **Supplementary File S9** reinforced this idea, illustrating the extensive regions of tandemly repeated sequences in the latter two proteins (*C. albicans* Als-like) and lack of a repeated domain in SsAls4579 (Sag1-like). *M. guilliermondii* was a similar mixing bowl of Als proteins: MgAls673 was the most Sag1-like of the group. Each of the other MgAls proteins had a long stalk of tandemly repeated units with NT domains that perhaps were more like Sag1 than Als3 (**Supplementary File S7**). *L. elongisporus* NT-Als proteins also appeared in more than one column in **Figure 4** with one (LeAls734) sharing greater similarity with Sag1. The recombinant nature of some of the LeAls proteins presented a more-complex picture than for *S. stipitis* or *M. guilliermondii* (**Supplementary File S6**).

### Drawing a Tree to Visualize NT-Als Relatedness

**Figure 5:**
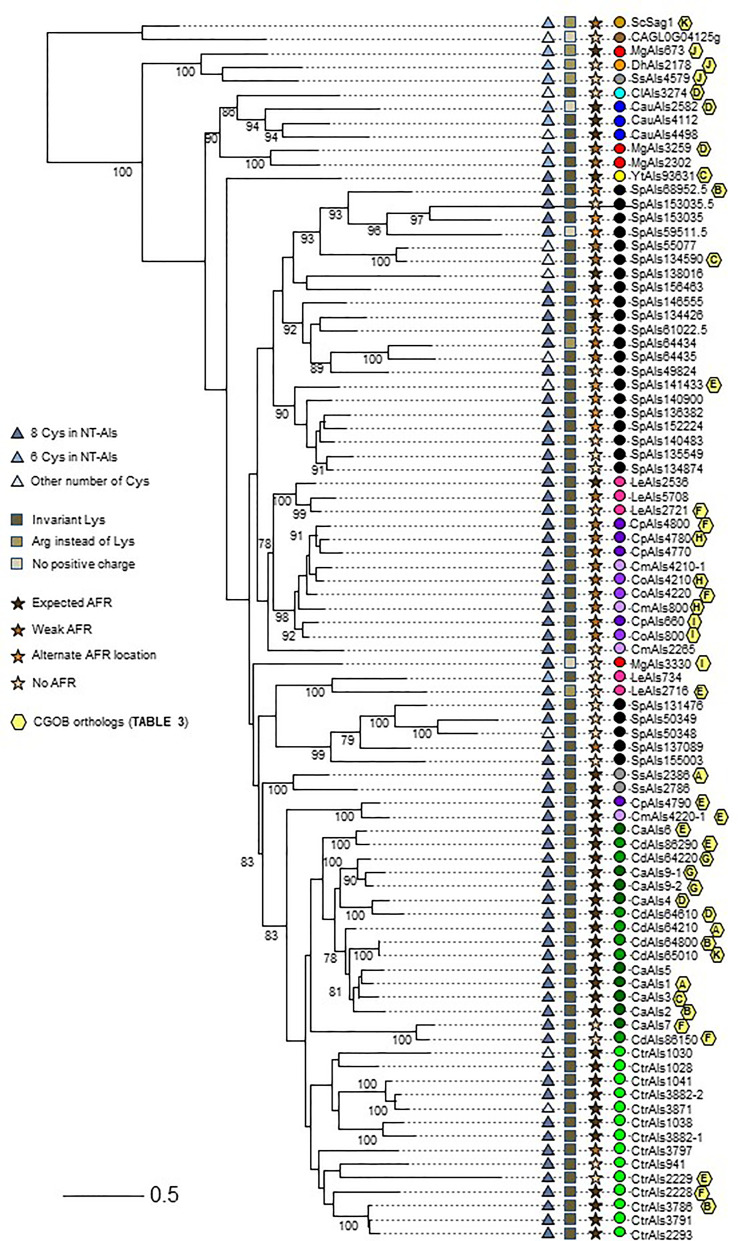
Relationships between NT-Als sequences depicted in a tree format. Information from **Figure 4** (number of Cys in NT-Als, presence of the invariant Lys, nature of the AFR) is included using symbols at the left of the tree. Yellow hexagons on the right of the branches depict ortholog group information from **Table 3**. Colored dots indicating species follow the color scheme from **Supplementary Table S2** and **Figure 4**. The scale bar represents substitutions per site.

In addition to comparing NT-Als proteins based on the features described above, more-global comparisons were made. Clustal Omega alignment of the NT-Als sequences (**Supplementary File S5**) showed identity values ranging from approximately 20% to 90% (data not shown). A maximum likelihood tree was drawn to visualize the results (**Figure 5****)**.

The topology of the tree reflected the overall diversity in NT-Als sequence identity. For example, NT-Als sequences from the closely related species *C. parapsilosis*/*C. orthopsilosis*/*C. metapsilosis* appeared on short branches in tight groups. In constrast, many branches showing the relationship between the highly diverse *S. passalidarum* NT-Als sequences were quite long, most notably for SpAls153035.5, which was the least related to other proteins in that species.

Als sequences from the same species tended to cluster together which made the overall tree organization similar to the species tree in **Figure 1**. The close relationship between *C. albicans* and *C. dubliniensis* (Jackson et al., 2009), as well as that between *C. parapsilosis*/*C. orthopsilosis*/*C. metapsilosis* (Tavanti et al., 2005), were reflected by the proteins from these groups being localized to distinct regions of the tree. *C. tropicalis* sequences were similarly clustered as were most of the *S. passalidarum* sequences. *C. auris* and *C. lusitaniae* sequences were grouped closely, similar to their position on the species tree (**Figure 1**). Within the groupings of closely related species, orthologs were obvious (e.g. CaAls6/CdAls86290, CaAls7/CdAls86150, CpAls660/CoAls800 and CoAls4210/CmAls4210-1). Examples of paralogs, likely the result of gene duplication, included CpAls4770/CpAls4780/CpAls4800, CtrAls3791/CtrAls2293/CtrAls3786, and SpAls64434/SpAls64435.

Similar to predictions made from the **Figure 4** diagram, NT-Als sequences from some species were spread across the tree. The four *M. guilliermondii* Als sequences were found in three different locations: MgAls673 was grouped more closely to sequences like ScSag1; MgAls2302 and MgAls3259 were clustered together near proteins from *C. auris*; and MgAls3330 was on its own branch elsewhere on the tree. Low bootstrap support for the latter location suggested that MgAls3330 could easily be located differently on the tree. The three sequences from *S. stipitis* were also dispersed with one near ScSag1 and the two others closer to the *C. albicans/C. dubliniensis* proteins. The five *L. elongisporus* Als proteins were split into two groups: the first including LeAls2536/LeAls5708/LeAls2721 (likely paralogs) and the second including LeAls734/LeAls2716 (recombinants between Als and Iff/Hyr). The Sag1-like features for SsAls4579 and MgAls673 were reflected on the tree (**Figure 5**), while LeAls734 and LeAls2716 (both Als recombinants with Iff/Hyr) clustered together in a different location.

The tree in **Figure 5** was drawn from a portion of each protein sequence. Another way to evaluate phylogenetic relationships is via conservation of physical location (synteny) as depicted using the *Candida* Gene Order Browser (CGOB; Maguire et al., 2013). CGOB includes all species in **Figure 5** except *C. glabrata* (*CAGL0G04125g*). A screenshot from one region of the CGOB was included in **Figure 2C**. It showed genes from several different fungal species that were orthologs of *C. albicans ALS6* and *ALS7*. Orthologs reported by CGOB are shown in **Table 3**. Yellow hexagons with alphabetical labels were used to display the CGOB relationships on the tree in **Figure 5**.

**Table 3:** Orthologous *ALS* loci reported on *Candida* Gene Order Browser (CGOB).

Reference Gene	Reported Ortholog	Group*	Reference Gene	Reported Ortholog	Group*
*CaALS1*	*CdALS64210*	**A**	*CaALS7*	*CdALS86150*	**F**
	*SsALS2386* ^†^			*CtrALS2228*	
				*CpALS4800*	
*CaALS2*	*CdALS64800*	**B**		*CoALS4220*	
	*CtrALS3786*			*LeALS2721*	
	*SpALS68952*				
			*CaALS9*	*CdALS64220*	**G**
*CaALS3*	*YtALS93631*	**C**		*LELG_04272* ^‡^	
	*SpALS134590*			*ScFLO1* ^‡^	

*CaALS4*	*CdALS64610*	**D**	*CpALS4780*	*CoALS4210*	**H**
	*CauALS2582*			*CmALS800* ^†^	
	*ClALS3274*				
	*PICST_31095* ^‡^		*MgALS3330*	*CpALS660*	**I**
	*MgALS3259*			*CoALS800*	

*CaALS6*	*CdALS86290*	**E**	*DhALS2178* ^§^	*SsALS4579*	**J**
	*CtrALS2229*			*MgALS673*	
	*CpALS4790*				
	*CmALS4220* ^†^		*ScSAG1*	*CdALS65010*	**K**
	*LeALS2716*				
	*SpALS141433*				

*Group designations were noted within yellow hexagons in **Figure 5**.

^†^For clarity, gene names from **Supplementary Table S2** were used here instead of the ORF designations on CGOB. SsALS2386 was called PICST_4539 on CGOB; CmALS4220 was called CMET_1838 on CGOB, and CmALS800 was called CMET_3970.

^‡^PICST_31095 belonged to the IFF/HYR family instead of the ALS family. LELG_04272 lacked an NT-Als domain but had tandem repeats and C-terminal sequence features similar to ALS genes. ScFLO1 belongs to a family of flocculins (Goossens et al., 2011). Although flocculins also have a central domain of repeated sequences, the structure of the mannose-binding N-terminal domain is clearly different from NT-Als (**Figure 3I**).

^§^Although not ALS genes, CaFGR23 (orf19.1616), Cd82280, and Ctr2409 were also in this ortholog pillar.

CGOB ortholog designations were most reliable in closely related species such as *C. albicans* and *C. dubliniensis*: each *C. albicans* locus had a *C. dubliniensis* ortholog except *CaALS3* and *CaALS5* which are missing from the *C. dubliniensis* genome (Jackson et al., 2009). These ortholog groups were frequently augmented with genes from other species such as Group A which included *SsALS2386*. Groups E and F were the largest, suggesting ortholog relationships between genes in 7 and 6 species, respectively.

CGOB also proposed ortholog groups that did not include *C. albicans* or *C. dubliniensis* sequences. One example was Group J that included the *SAG1*-like *MgALS673* and *SsALS4579*, as well as *DhAls2178*, the lone *ALS* sequence from the primarily haploid *D. hansenii*. Examination of the predicted protein sequence showed lack of a tandem repeat domain as well as other Sag1-like features (**Supplementary File S11** and **Figure 4**).

Some CGOB ortholog designations were perhaps problematic. For example, *PICST_31095* (Group D) belonged to the *IFF/HYR* family instead of *ALS* and *ScFLO1*(Group G) encoded a flocculin with a mannose-binding N-terminal domain that was structurally diverse from the Als family (**Figure 3**). Perhaps the most intriguing ortholog designation was Group K which included *ScSAG1* and *CdALS65010*. *CdALS65010* was nearly 100% identical to *CdALS64800*, which was included in ortholog Group B. These loci are near each other on *C. dubliniensis* chromosome 6 and the appear to be paralogs (i.e. recently duplicated; Jackson et al., 2009). As species become more divergent, assessing ortholog designations becomes more challenging. However, the suggestion of orthology between *ScSAG1* and a *C. dubliniensis ALS* gene supported the general idea that the genes were part of the same family and arose from a common ancestor.

### Probing the Taxonomic Boundaries of the *ALS* Family

**Figure 6:**
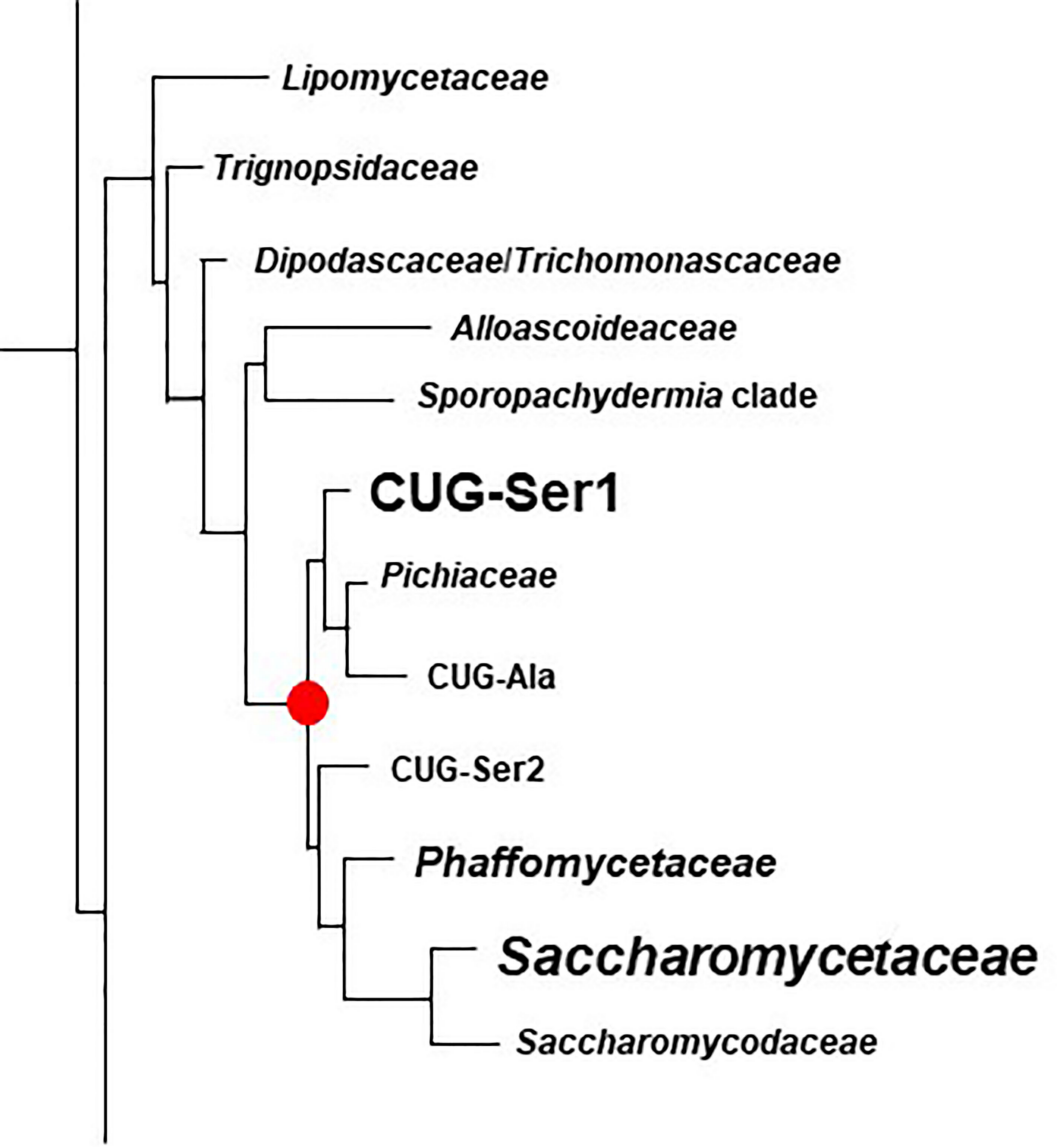
Phylogenetic tree of Families in the Order Saccharomycetales. The tree was traced from the data of Li et al. (2021) to highlight the region of interest for the current study. Family names were those used by Li et al. (2021). **Table 4** placed these names into the context of Family designations used on the NCBI Taxonomy Database (Schoch et al., 2020). The red dot denotes the common ancestor of the CUG-Ser1 clade and *Saccharomycetaceae*, the two groups in which *ALS* genes were most common (indicated by larger font). *ALS* genes were also detected in the *Phaffomycetaceae*.

The rapidly increasing number of genome sequences present in public databases provided an opportunity to further explore the breadth and potential origin of the *ALS* family. The NCBI database can be queried using taxonomic identification (taxid) numbers to detect potential *ALS* loci more broadly across the phylogenetic tree. **Table 4** lists the taxids for designations of the major Families within the Order Saccharomycetales. Inputs from NCBI (lifemap-ncbi-univ-lyon1.fr), Shen et al. (2018), and Li et al. (2021) were included although the taxonomic designations were not identical among the sources. The portion of the kingdom Fungi tree (Li et al., 2021) that represented the Subphylum Saccharomycotina was reproduced in **Figure 6**.

**Table 4:** *ALS* gene representation within the Order Saccharomycetales*.

Family	Genus/species	# *ALS* Loci
*Debaryomycetaceae* (taxid:766764); included in CUG-Ser1 (**Figure 6**)	*Candida albicans* (taxid:5476)	8
	*Candida dubliniensis* (taxid:42373)	7
	*Candida tropicalis* (taxid:5482)	13
	*Candida parapsilosis* (taxid:5480)	5
	*Candida orthopsilosis* (taxid:273371)	3
	*Candida metapsilosis* (taxid:273372)	4
	*Lodderomyces elongisporus* (taxid:36914)	5
	*Debaryomyces hansenii* (taxid:4959)	1
	*Meyerozyma guilliermondii* (taxid:4929)	4
	*Scheffersomyces stipitis* (taxid:4924)	3
	*Spathaspora passalidarum* (taxid:340170)	29
	*Yamadazyma tenuis* (taxid:2315449)	1
*Metschnikowiaceae* (taxid:27319); included in CUG-Ser1 (**Figure 6**)	*Clavispora lusitaniae* (taxid:36911)	1
	*Candida auris* (taxid:498019)	3
*Saccharomycetaceae* (taxid:4893)	*Saccharomyces cerevisiae* (taxid:4932)	1
	*Candida glabrata* (taxid:5478)	1
*Pichiaceae* (taxid:1156497)	*Pichia kudriavzevii* (taxid:4909)	0
		
	**Number of Species with an** **Available Genome Sequence** **in the NCBI Database** **(Accessed September 2021)**	**Potential *ALS* Loci**
*Lipomycetaceae* (taxid:29827)	9	0
*Trignopsidaceae* (taxid:1540145)	6	0
*Dipodascaceae* (taxid:34353)	32	0
*Trichomonascaceae* (taxid:410830)	26	0
*Alloascoideaceae* (taxid:1540229)	1	0
*Sporopachydermia* clade (taxid:45793)	2	0
*Cephaloascaceae* (taxid:28984); included in CUG-Ser1 (**Figure 6**)	2	0
*Pichiaceae* (taxid:1156497)	60	0
*Saccharomycopsidaceae* (taxid:34366); overlap with CUG-Ser2 (**Figure 6**)	9	1
*Phaffomycetaceae* (taxid:115784)	39	14
*Saccharomycodaceae* (taxid:34365)	21	0
*Saccharomycetales incertae sedis* (taxid:241407)	37	4

*Family designations were from lifeman-ncbi-univ-lyon1.fr, as well as the work of Shen et al. (2018) and Li et al. (2021). Taxid numbers were from the NCBI Taxonomy Database (https://www.ncbi.nlm.nih/gov/taxonomy). Numbers of ALS loci (right column; top half of table) were taken from the genome sequences examined in this study (**Table 1**).

**Figure 6** indicated the position of a common ancestor (red dot) for the *ALS* family that would include both *S. cerevisiae SAG1* (in the *Saccharomycetaceae*) and genes more closely resembling *C. albicans ALS* loci (in CUG-Ser1). BLAST searching using taxids did not reveal *ALS* loci in available genome sequences from the *Lipomycetaceae*, *Trignopsidaceae*, *Dipodascaceae*, *Trichomonascaceae*, *Alloascoidaceae*, or the *Sporopachydermia* clade (**Table 4**). CUG-Ser1 was comprised of the *Debaryomycetaceae*, *Metschnikowiaceae*, and *Cephaloascaceae* (Shen et al., 2018). BLAST searching using taxids suggested that there were numerous additional *ALS* genes among the 88 *Debaryomycetaceae* species genomes in NCBI (accessed September 2021). Among the 62 *Metschnikowiaceae* species, *ALS* sequences were found primarily in species closely related to *C. auris* and *C. lusitaniae* including *Candida haemulonii*, *Candida duobushaemulonis*, and *Candida pseudohaemulonii*, all of which have been observed as pathogens (Jurardo-Martin et al., 2020). *C. intermedia*, noted for its ability to ferment xylose, as well as a rare cause of catheter-related fungemia (Ruan et al., 2010; Geijer et al., 2020) also had *ALS* sequences. No *ALS* sequences were detected in the *Cephaloascaceae*, although only 2 genomes were available (**Table 4**).

*Pichia kudriavzevii* (formerly *Candida krusei*; Douglass et al., 2018) was included in **Table 4** and in **Figure 1** since the species is among those associated with clinical disease (Gomez-Gaviria and Mora-Montes, 2020). However, *ALS* genes were not detected in *Pichia kudriavzevii*, nor in the genomes of 60 other species from the Family *Pichiaceae* that were available in the NCBI database (**Table 4**). No *ALS* sequences were detected in the species included in the CUG-Ala clade (**Figure 6**). The lone BLAST hit in the *Saccharomycopsidaceae* (**Table 4**; overlap with CUG-Ser2 in **Figure 6**) was based more on the presence of Ser/Thr-rich repeated sequences than on an NT-Als adhesive domain. Additional work would be required to validate the sequence and determine if an NT-Als domain was present.

Potential *ALS* genes in the *Phaffomycetaceae* (**Table 4**) were located in two species each in the genera *Wickerhamomyces* and *Cyberlindnera*; each locus encoded a potential NT-Als domain. Many additional *ALS* sequences were found among the 104 *Saccharomycetaceae* genomes available in NCBI. This family included *S. cerevisiae*, for which *SAG1* was the only BLAST hit. For other species such as *Torulospora delbrueckii*, a species of interest for wine making and baking (Fernandes et al., 2021), more than one *ALS* sequence was detected in the genome. One (*TDEL_0E04030*) predicted a protein that more closely resembled *S. cerevisiae* Sag1, while another (*TDEL_0G04810*) encoded an NT-Als domain (validated using AlphaFold; data not shown) attached to a complex C-terminal Ser/Thr-rich sequence. This predicted C-terminal domain was notable because it included 5 copies of the 45-amino acid tandem repeat found in *S. cerevisiae* Flo1 (Soares, 2010), suggesting recombination between the *ALS* and *FLO* families. A predicted protein in *Lachancea thermotolerans* (KLTH0C00440p) suggested an even-more-convincing recombinant that had an NT-Als domain attached to > 30 copies of the Flo1 tandem repeat. These sequences require verification to ensure the recombinants were part of the genome, rather than an artifact of computational genome assembly. *ALS* sequences were also detected in *Kazachstania* species extending the representation of *ALS* sequences throughout the *Saccharomycetaceae* as defined by Shen et al. (2018). Many of these sequences appeared partial, suggesting the need for PCR amplification and repair to reveal the true nature of their encoded proteins. No *ALS* sequences were detected among the 21 *Saccharomycodaceae* species queried.

The taxonomic category *Saccharomycetales incerta sedis* had four *ALS* sequences, all in the species *Diutina rugosa* (formerly *Candida rugosa*), which is noted as an infrequent human pathogen (Ming et al., 2019). Evaluation of the best hit (*DIURU_002000*) showed a predicted secretory signal peptide and GPI anchor addition site. Within the NT-Als region, DIURU_002000 was 37% identical to *C. albicans* NT-Als3, had 8 Cys in the expected locations, Arg instead of the invariant Lys, and a strong predicted AFR. AlphaFold produced the same general structure for the DIURU_002000 NT-Als protein as for those shown in **Figure 3** (data not shown).

Collectively, these observations pinpointed the location of the *ALS* family among currently available genome sequences and suggested potential taxonomic boundaries that will continue to be tested as more genome sequences are added to this region of the phylogenetic tree.

## Discussion

Availability of genome resources for pathogenic yeasts, as well as other closely related species, provided a comparative view of the *ALS* family across a broader expanse of the phylogenetic tree than previously investigated. Despite advances in long-read DNA sequence technologies, many *ALS* loci still required PCR amplification and Sanger sequencing to derive an accurate gene count and sequence. The availability of the unprecedented protein structural prediction accuracy of AlphaFold (Jumper et al., 2021) provided the opportunity to demonstrate conservation of NT-Als domain shape for the proteins listed in **Supplementary Table S2**. Recent addition of more than 200 new budding yeast genome sequences into the NCBI database (Shen et al., 2018) further bridged the gap between *S. cerevisiae* and *C. albicans* and provided a clearer picture of the *ALS* family breadth. In total, the information leads to the conclusions that *S. cerevisiae* Sag1 is an Als protein and the Als family is far more diverse than previously described.

Presence of the NT-Als domain is universal among the proteins in **Supplementary Table S2**, and clearly is a defining characteristic for inclusion in the Als family. The AFR is recognized as part of the NT-Als domain (Lin et al., 2014); this sequence was absent, weaker, or perhaps in another location in many of the newly characterized Als proteins described here. Immediately following the AFR in *C. albicans* Als proteins is a short Thr-rich region that some publications refer to as the T domain (reviewed in Hoyer and Cota, 2016). The T domain in *C. albicans* Als proteins ends at the start of the tandem repeat region. Designating a T domain in many of the proteins described here would require arbitrary judgement calls so the region was not included in any of the line diagrams (**Supplementary Files S6****-****S11**). NT-Als domains were drawn to include all amino acids up to the start of the repeated sequences or Ser/Thr-rich regions.

In some proteins (e.g. SpAls55077, SpAls134590, ClAls3274), sequences similar to the *C. albicans* T domain are expanded into repeated sequences of variable length in the center of the protein. While all *C. albicans* Als proteins have a region of highly conserved, 36-amino-acid tandemly repeated sequences, this part of the Als protein is quite variable among those from other species. Variations include tandem copies of novel lengths and sequences, repeated sequences that are not necessarily tandem copies, and complete lack of repeated sequences. The latter presentation is characteristic of *S. cerevisiae* Sag1 and other proteins more closely related to ScSag1 on the **Figure 5** tree. Sequences most C-terminal in the Als family are also diverse, many featuring an enriched Ser/Thr composition. Ser/Thr richness promotes O-glycosylation that can form an extended structure on which the NT-Als domain is displayed on the cell surface (Jentoft, 1990). Some of the Als proteins have other amino acids (e.g. Glu, Gly, Pro) that are also enriched in the C-terminal portion of the protein or in novel, short repeated sequences.

Inclusion in the Als family is focused on the presence of an NT-Als domain that has the same general shape as the proteins described here (**Figure 3**). This Als family definition also recognizes instances of recombination between *ALS* genes and others encoding cell-surface proteins. A previous report (Oh et al., 2019) described *CmALS2265*, the product of recombination between *ALS* genes and the *IFF/HYR* family (Bailey et al., 1996; Bates et al., 2007; Chaffin, 2008; Boisramé et al., 2011; Riccombeni et al., 2012). In the present study, additional recombinants between *ALS* and *IFF/HYR* genes were noted in *L. epongisporus* (*LeALS734* and *LeALS2716*). The tantalizing possibility of *ALS/FLO* recombinants in *T. delbrueckii* and *L. thermotolerans* suggests even-greater interfamily recombination as a mechanism for generating diversity on the fungal cell surface. Recombination, as the mechanism to generate novel adhesins, has been discussed previously (Verstrepen et al., 2004; Verstrepen et al., 2005) although these descriptions involved recombination between genes in the same family. In contrast, interfamily recombination places the functional NT domain from one family onto the glycosylated stalk from another. Predicted structures for the NT domains of Als, Iff/Hyr, and Flo proteins were included in **Figure 3** to highlight their obvious differences. Potential for hybrid function expands if the glycosylated stalk is more than just a pedestal for displaying the NT domain. These observations provide ample experimental hypotheses to pursue to better understand cell-surface diversity among the yeast species.

Another novel observation from the current work is the subtelomeric localization of *ALS* genes in multiple species including *M. guilliermondii* (*MgALS2302*, *MgALS3259*), *C. auris* (*CauALS2582*), and *S. passalidarum* (*SpALS131476*, *SpALS137089*). *C. albicans* subtelomeric regions are described as hotspots for genomic rearrangement, although adhesin-encoding genes are not located there (Dunn and Anderson, 2019). Subtelomeric location of adhesin genes is well-documented in other species such as *C. glabrata* (Xu et al., 2020). However, analysis of long-read-based genome sequences from three *C. glabrata* isolates suggested that changes in subtelomeric adhesin-encoding genes is due to break-induced replication, a relatively rare event that affects only the gene closest to the telomere (Xu et al., 2021). Muñoz et al. (2021) noted *C. auris* subtelomeric regions rich in genes encoding Iff/Hyr proteins, candidate cell wall proteins unique to that species, and CauAls2582. Analysis of genome sequences from 12 C*. auris* isolates representing diverse clades showed that Clade II isolates lose large subtelomeric regions and the cell wall genes associated with them (Muñoz et al., 2021). The consequences of *ALS* gene location in the subtelomeric region remain to be further explored in species such as *M. guilliermondii* and *S. passalidarum*.

By including Sag1 in the Als family, the definition of Als function also becomes more diverse. Sag1 binds a very specific ligand (the C-terminal peptide of **a**-agglutinin; Cappellaro et al., 1994) with high affinity. In comparison, *C. albicans* Als proteins can bind many ligands with lower affinity and rely on AFR-mediated protein aggregation to increase avidity (reviewed in Hoyer and Cota, 2016; Ho et al., 2019). Similar to those in *C. albicans*, several of the more-recently described Als proteins in other species function in adhesion to host cells or protein-coated surfaces (Bertini et al., 2016; Neale et al., 2018; Zoppo et al., 2018; Lombardi et al., 2019; Zoppo et al., 2020a; Zoppo et al., 2020b). However, considerable effort is still required to understand the specifics of these interactions. Information in **Figures 3**, **5** can be used to select proteins from along the newly defined Als family spectrum to better understand how structure contributes to functional variation. Experiments involving purified proteins and biophysical techniques could be pursued, or perhaps individual genes or chimeric constructs could be expressed in model systems such as the recently described *ALS*-negative *C. orthopsilosis* strain (Zoppo et al., 2019). Experimentally demonstrated structural solutions could also be pursued, and in place of the difficult-to-crystallize ScSag1, proteins close to ScSag1 on the **Figure 5** tree may serve as informative surrogates (e.g. Group J and CAGL0G04125G, which may be mating glycoproteins from the haploid species *M. guilliermondii*, *D. hansenii*, and *S. stipitis*).

The *ALS* alleles listed in **Supplementary Table S2** are quite long: many are at least 5 kb and one is over 15 kb. In *C. albicans*, where multiple strains and *ALS* alleles have been examined, these long ORFs are maintained without interruption. One notable example is *C. albicans ALS7* that experiences frequent expansions and contractions of multiple different repeated sequences and is transcribed at an extremely low level that may not produce sufficient protein for biologically meaningful adhesive interactions (Zhang et al., 2003; Green et al., 2005). In contrast, the current study revealed the first example of *ALS* pseudogenes. They are found in *S. passalidarum* that has amplified the *ALS* family to 29 loci, well beyond the census in other characterized species (**Supplementary File S10**). In addition to the *ALS* pseudogenes, many of the other *S. passalidarum ALS* loci encode proteins that are predicted to mislocalize compared to the *C. albicans* model: lack of a secretory signal peptide suggests protein that accumulates inside the cell rather than on the surface. The presence of loci that encode proteins with secretory potential, but lacking the ability to cross-link into the cell wall (i.e. no GPI anchor addition site), suggests the potential for *S. passalidarum* to export its own adhesion inhibitors. For example, a secreted, high-affinity binding protein could inhibit *S. passalidarum* adhesion to host cells, or perhaps release individual cells from host interactions or from polymicrobial structures such as biofilms. Wohlbach et al. (2011) pointed out that the wood-boring-beetle-associated *S. passalidarum* is also a commensal, similar to the typical relationship between humans and many of the fungi described here. However, only two of the SpAls proteins include all NT-Als features (8 Cys, invariant Lys, AFR) that are important for function of *C. albicans* Als3 (**Supplementary Table S2**; **Supplementary File S10**; **Figure 4**). The long branches associated with the *S. passalidarum* Als sequences in **Figure 5** could result from sequence substitutions due to lack of selective pressure or could indicate functional diversification that serves *S. passalidarum* in its ecological niche. The role of the Als proteins in *S. passalidarum* is another topic with many hypotheses for future exploration.

Additional hypotheses to test involve allelic variability and gene expression. Information reported here is limited to one allele from each species. Previous work in *C. albicans* revealed tremendous allelic variation, often involving copy numbers of repeated sequences, but sometimes affecting the NT-Als coding region (reviewed in Hoyer et al., 2008). Considerable variation is frequently observed between alleles within the same strain of a diploid species (Zhao et al., 2003; Zhao et al., 2007). It is reasonable to hypothesize that allelic variation exists among the genes and species described here. It is also reasonable to expect examples of differential gene expression with growth stage and morphology, gene expression variations between strains of the same species, and gene expression patterns that differ between *in vitro* and *in vivo* growth conditions (reviewed in Hoyer et al., 2008; Coleman et al., 2012; Galán-Ladero et al., 2019; Lombardi et al., 2019). There are many exciting avenues for investigation as the nature of the *ALS* family continues to be discovered.

## Data Availability Statement

The genome assembly for Lodderomyces elongisporus strain NRRL YB-4239, generated using Illumina MiSeq and Oxford Nanopore MinION data, is available in GenBank under BioProject accession number PRJNA432415, BioSample accession number SAMN08448784, and Genome Assembly accession number GCA_013620985.1 (ASM1362098v1). The genome assembly for Meyerozyma guilliermondii strain ATCC 6260, generated using Illumina MiSeq and Oxford Nanopore MinION data, is available in GenBank under BioProject accession number PRJNA432394, BioSample accession number SAMN08448312, and Genome Assembly accession number GCA_006942155.1 (ASM694215v1). The genome assembly for Scheffersomyces stipitis strain CBS 6054, generated using Illumina MiSeq and Oxford Nanopore MinION data, is available in GenBank under BioProject accession number PRJNA432364, BioSample accession number SAMN08446935, and Genome Assembly accession number GCA_006942115.1 (ASM694211v1). The genome assembly for Spathaspora passalidarum strain NRRL Y-27907, generated using Illumina MiSeq and Oxford Nanopore MinION data, is available in GenBank under BioProject accession number PRJNA432249, BioSample accession number SAMN08439027, and Genome Assembly accession number GCA_013620965.1 (ASM1362096v1).

## Author Contributions

LH conceptualized the study, acquired funding, and administered the project. KS, VV, CF, and AH conducted formal analysis. S-HO, AI, RR-B, CF, AH, and LH performed the investigation. CF, AH, and LH developed the study methodology. S-HO, KS, CF, AH, and LH wrote the original draft. All authors contributed to the article and approved the submitted version.

## Funding

This work was funded by R15 DE026401 from the National Institute of Dental and Craniofacial Research, National Institutes of Health.

## Conflict of Interest

The authors declare that the research was conducted in the absence of any commercial or financial relationships that could be construed as a potential conflict of interest.

## Publisher’s Note

All claims expressed in this article are solely those of the authors and do not necessarily represent those of their affiliated organizations, or those of the publisher, the editors and the reviewers. Any product that may be evaluated in this article, or claim that may be made by its manufacturer, is not guaranteed or endorsed by the publisher.
