# Supplementary material for: Using Genomics to Shape the Definition of the Agglutinin-Like Sequence (ALS) Family in the Saccharomycetales
Source: Front Cell Infect Microbiol. 2021 Dec 14;11:794529. doi: 10.3389/fcimb.2021.794529 (PMC8712946; doi:10.3389/fcimb.2021.794529)
Supplement: Supplementary file 1 [file DataSheet_1.docx]

**SUPPLEMENTARY FILE S1 |** Details for assembly of *Lodderomyces elongisporus* NRRL YB-4239 genome sequence data.

**Assembly of *L. elongisporus***


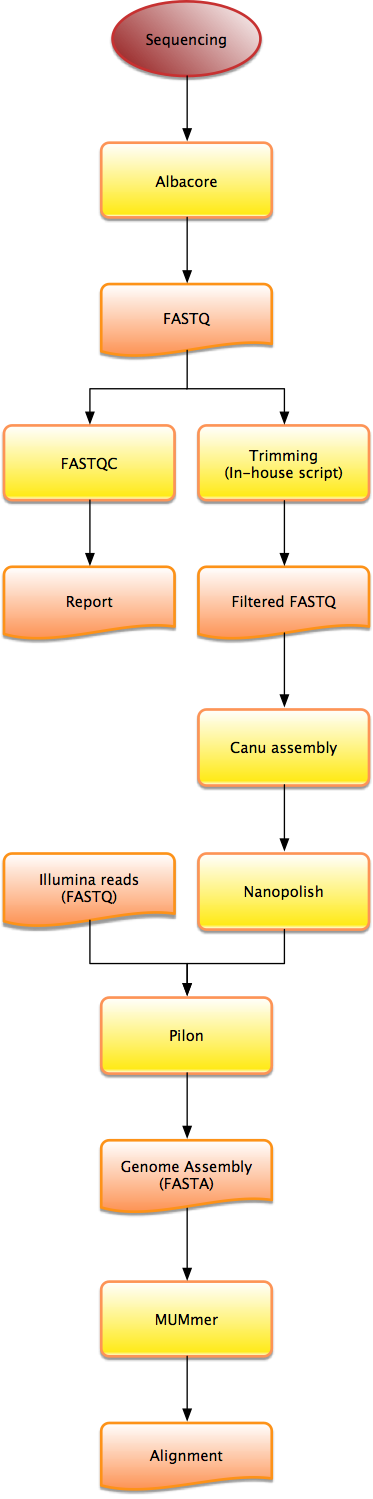


## Description of Library prep and sequencing.

1 ug of genomic DNA were sheared in a gTube (Covaris, Woburn, MA) for 1 min at 6,000 rpm in an Eppendorf MiniSpin plus microcentrifuge (Eppendorf, Hauppauge, NY).

The sheared DNA was converted into a Nanopore library with the Nanopore Sequencing kit SQK-LSK108 and the Native 1D Barcoding kit EXP-NBD103 (Oxford Nanopore)

The 5 libraries were pooled in equimolar concentration and the pool was sequenced on two SpotON MK I R9.5 flowcells for 24 h, using a MinION MK 1B sequencer. Basecalling was done in real time with software MinKNOW version 1.7.7.

## Data processing steps

### Albacore

ONT Albacore Sequencing Pipeline Software version 1.2.4 was used to demultiplex fast5 files from MinKNOW 1.7.7 basecalling program. The albacore command is:

"read_fast5_basecaller.py -i pass -t 14 -s newAlbacoreResults -k SQK-LSK108 -f FLO-MIN107 --barcoding --recursive -o fast5,fastq"

The following table shows the sequence counts and lengths for *L. elongisporus nanopore reads.*

| Total bases: | 641,131,396 |
| --- | --- |
| Number of reads: | 64,450 |
| Mean read length (bp): | 9,947 |
| Median read length (bp): | 9,551 |
| Min read length (bp): | 177 |
| Max read length (bp): | 196,238 |

### FastQC

FastQC v-0.11.4 software was also used to further access quality scores and other attributes of the data sets. The following figures were selected from the FastQC reports.

#### FastQC results from L_elongisporus_raw.fastq data


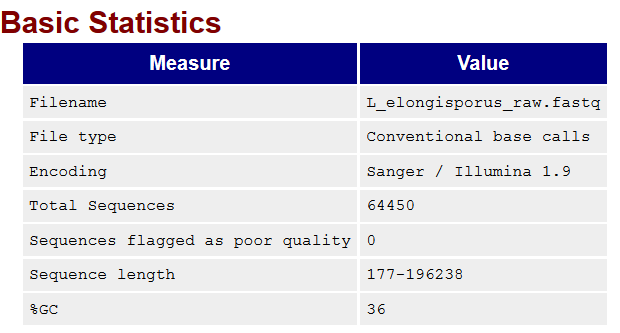


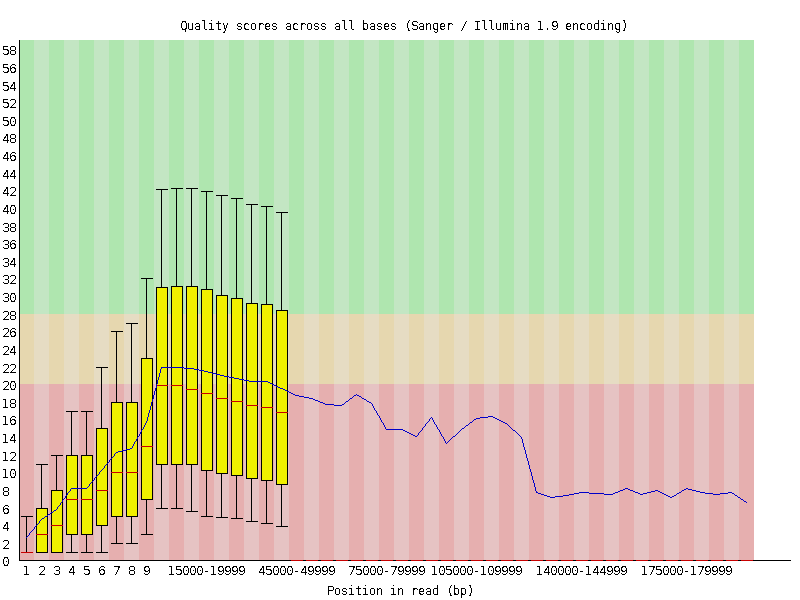


#### FastQC results from L_elongisporus_trimmed.fastq data


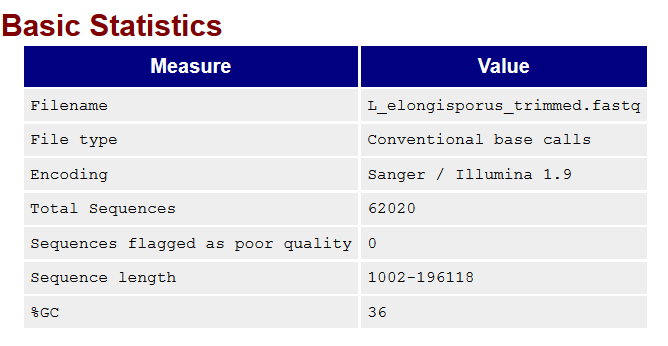


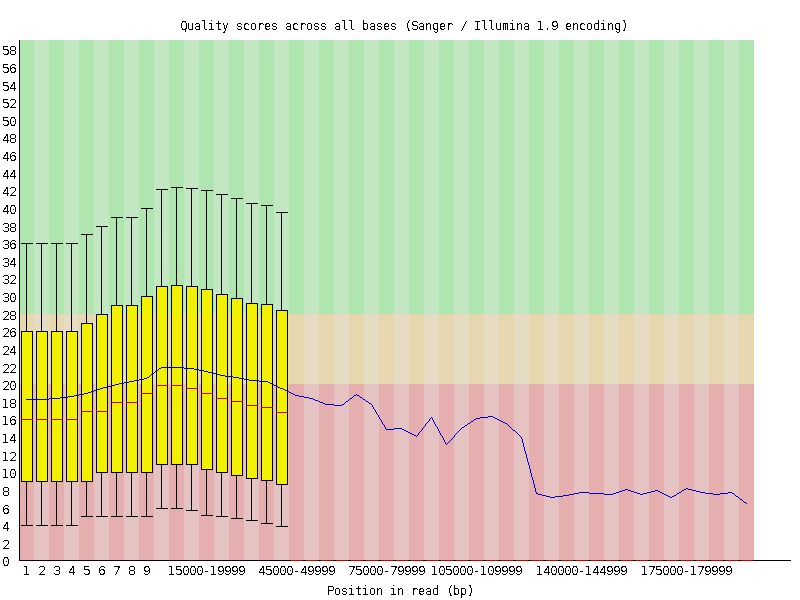


### Barcode trimming and low quality base removal

An in-house Perl script was used to trim 60 bases from the both ends of raw nanopore reads. Reads that are longer than 1,000 bps were selected for assembling purposes. For the Illumina MiSeq reads, Trimmomatic-0.36 was used to remove quality scores less than 30 as well as reads < 50 bp in length

### Canu-v1.5 Assembly

A total of 62,020 *L. elongisporus* reads were used in Canu-v1.5. The assembly contains 53 contigs. The following table shows the contigs and their lengths.

| **L_elongiCanuAsm.fasta**  **(53 Contigs)** | | |
| --- | --- | --- |
| Contig Name | Length | Reads in  the contig |
| >tig00000136 | 3,175,787 | 8,179 |
| >tig00000060 | 1,405,343 | 3,474 |
| >tig00000140 | 888,906 | 2,565 |
| >tig00000073 | 764,188 | 2,030 |
| >tig00000057 | 749,381 | 2,050 |
| >tig00000155 | 742,491 | 1,990 |
| >tig00000161 | 732,435 | 1,971 |
| >tig00000039 | 704,668 | 2,135 |
| >tig00000160 | 557,433 | 1,457 |
| >tig00000017 | 528,042 | 1,377 |
| >tig00000143 | 499,365 | 1,367 |
| >tig00000158 | 495,027 | 1,201 |
| >tig00000166 | 400,939 | 1,049 |
| >tig00000157 | 393,306 | 1,010 |
| >tig00000099 | 384,589 | 1,028 |
| >tig00000138 | 382,622 | 927 |
| >tig00000043 | 367,914 | 1,236 |
| >tig00000025 | 367,157 | 900 |
| >tig00000102 | 349,339 | 1,218 |
| >tig00000151 | 312,427 | 897 |
| >tig00000142 | 300,243 | 858 |
| >tig00000105 | 263,803 | 539 |
| >tig00000165 | 143,524 | 323 |
| >tig00000168 | 127,850 | 299 |
| >tig00000050 | 108,882 | 332 |
| >tig00000145 | 96,308 | 236 |
| >tig00000124 | 94,273 | 3,249 |
| >tig00000152 | 87,936 | 184 |
| >tig00000163 | 52,562 | 87 |
| >tig00000149 | 40,413 | 87 |
| >tig00000086 | 39,159 | 58 |
| >tig00000148 | 39,025 | 37 |
| >tig00000076 | 38,465 | 45 |
| >tig00000139 | 37,561 | 195 |
| >tig00000120 | 37,297 | 86 |
| >tig00000047 | 36,922 | 22 |
| >tig00000129 | 35,190 | 31 |
| >tig00000083 | 35,007 | 32 |
| >tig00000081 | 34,500 | 33 |
| >tig00000066 | 32,065 | 82 |
| >tig00000065 | 30,861 | 14 |
| >tig00000004 | 30,411 | 22 |
| >tig00000171 | 28,740 | 56 |
| >tig00000122 | 26,817 | 20 |
| >tig00000164 | 26,131 | 31 |
| >tig00000137 | 25,255 | 24 |
| >tig00000141 | 24,268 | 65 |
| >tig00000048 | 23,250 | 22 |
| >tig00000014 | 21,216 | 15 |
| >tig00000175 | 21,146 | 17 |
| >tig00000153 | 17,203 | 18 |
| >tig00000054 | 15,182 | 6 |
| >tig00000150 | 14,331 | 7 |
| Total bases | 16,187,155 |  |

#### Canu-v1.5 Commands:

module load Canu/1.5-IGB-gcc-4.9.4-Perl-5.24.1

module load Java/1.8.0_121

module load gnuplot/5.0.6-IGB-gcc-4.9.4

canu -p asm -d defaultAsm genomeSize=15.5m useGrid=false \

-nanopore-raw L_elongisporus_trimmed.fastq

### Nanopolish-v0.7.1

The consensus sequences from the assembly were added to Nanopolish v0.7.1 for error correction. Nanopolish uses the signal-level data from the nanopore with a novel hidden Markov model to achieve a more accurate assembly.

**Nanopolish commands:**

module load nanopolish/0.7.1-IGB-gcc-4.9.4-159d92b

module load parallel/20170622-IGB-gcc-4.9.4

module load BWA/0.7.15-IGB-gcc-4.9.4

module load SAMtools/1.5-IGB-gcc-4.9.4

nanopolish extract fast5 > reads.fasta

bwa index L_elongisporusCanuAsm.fasta

bwa mem -x ont2d -t 12 L_elongisporusCanuAsm.fasta reads.fasta | \

samtools sort -o reads.sorted.bam -T reads.tmp -

samtools index reads.sorted.bam

python nanopolish_makerange.py L_elongisporusCanuAsm.fasta | parallel \

--results nanopolish.results -P 12 \

nanopolish variants --consensus polished.{1}.fa -w {1} \

-r reads.fasta \

-b reads.sorted.bam -g L_elongisporusCanuAsm.fasta -t 4 \

--min-candidate-frequency 0.1

python nanopolish_merge.py polished.*.fa > \

L_elongisporusCanuAsmPolished.fasta

### PILON-v1.22

#### Reads from the Illumina paired end MiSeq run were trimmed with Trimmomatic v-0.36. The nucleotide bases with quality scores less than 30 were removed. Reads > 50 bp in length were selected. As a result, a total of 6,126,514 reads were used to align to the error corrected genome from Nanopolish with BWA v-0.7.15. . The bam file from BWA and the corrected genome were used as input data for PILON software.

#### PILON-v1.22 Commands:

module load pilon/1.22-Java-1.8.0_121

module load BWA/0.7.15-IGB-gcc-4.9.4

module load SAMtools/1.5-IGB-gcc-4.9.4

# Index the draft genome

draftAsm=L_elongisporusCanuAsmPolished.fasta

bwa index ${draftAsm}

R1= trimmed_Lodderomyces_elongisporus_CCGTCCCG_L001_R1_001.fastq

R2= trimmed_Lodderomyces_elongisporus_CCGTCCCG_L001_R2_001.fastq

# Align paired end reads to the draft assembly

bwa mem -x ont2d -t 12 $draftAsm $R1 $R2 | samtools sort -o pe_aln_sorted.bam -T reads.tmp -

samtools index pe_aln_sorted.bam

#run pilon

java -Xmx20G -jar pilon-1.22.jar --genome $draftAsm --bam pe_aln_sorted.bam --outdir out

1. **Mummer Plots**

**STATS OF FINAL ASSEMBLY**

| **Total bases:** | 16,382,369 |
| --- | --- |
| **Number of contigs:** | 53 |
| **N50 contig length (bp):** | 742,134 |
| **Mean contig length:** | 309,101 |
| **Median contig length:** | 95,638 |
| **Min contig length (bp):** | 14,428 |
| **Max contig length (bp):** | 3,212,662 |

### Comparison to NCBI reference genome

Nucleotide level comparisons between *L. elongisporus* reference genome from NCBI (GCA_000149685.1_ASM14968v1_genomic.fna) and the newly constructed assembly were done with the dnadiff program from MUMmer v-3.23.

#### dnadiff Results:

|  | GCA_000149685.1_ASM14968v1_genomic.fna | L_elongisporusCanuAsmPolishedPilon.fasta |
| --- | --- | --- |
| TotalSeqs | 28 | 53 |
| AlignedSeqs | 28(100.00%) | 53(100.00%) |
| AlignedSeqs | 28(100.00%) | 53(100.00%) |
| TotalBases | 15,547,272 | 16,382,369 |
| AlignedBases | 15,388,008(98.98%) | 16,267,513(99.30%) |
| UnalignedBases | 159,264(1.02%) | 114,856(0.70%) |
| TotalLength | 15,571,357 | 15,517,206 |
| AvgLength | 30,712.74 | 30,605.93 |
| AvgIdentity | 98.84 | 98.84 |
| Insertions | 523 | 809 |
| InsertionSum | 360,640 | 1,302,188 |
| InsertionAvg | 689.56 | 1,609.63 |
| TotalSNPs | 63,422 | 63,422 |
| TotalGSNPs | 25,189 | 25,189 |
| TotalIndels | 108,865 | 108,865 |
| TotalGIndels | 7,791 | 7,791 |

Mummerplot from MUMmer was used to create a graphical representation of the alignments for the newly constructed assembly and GCA_000149685.1_ASM14968v1 genome from NCBI.

#### MUMmer-3.23 commands:

module load MUMmer

# create report

dnadiff GCA_000149685.1_ASM14968v1_genomic.fna\

L_elongisporusCanuAsmPolishedPilon.fasta

# create plot

nucmer -maxmatch GCA_000149685.1_ASM14968v1_genomic.fna\

L_elongisporusCanuAsmPolishedPilon.fasta

delta-filter -m out.delta > out.delta.m

mummerplot –large –layout out.delta.m

#### **Mummer Plot:** **GCA_000149685.1_ASM14968v1_genomic.fna vs L_elongisporusCanuAsmPolishedPilon.fasta**


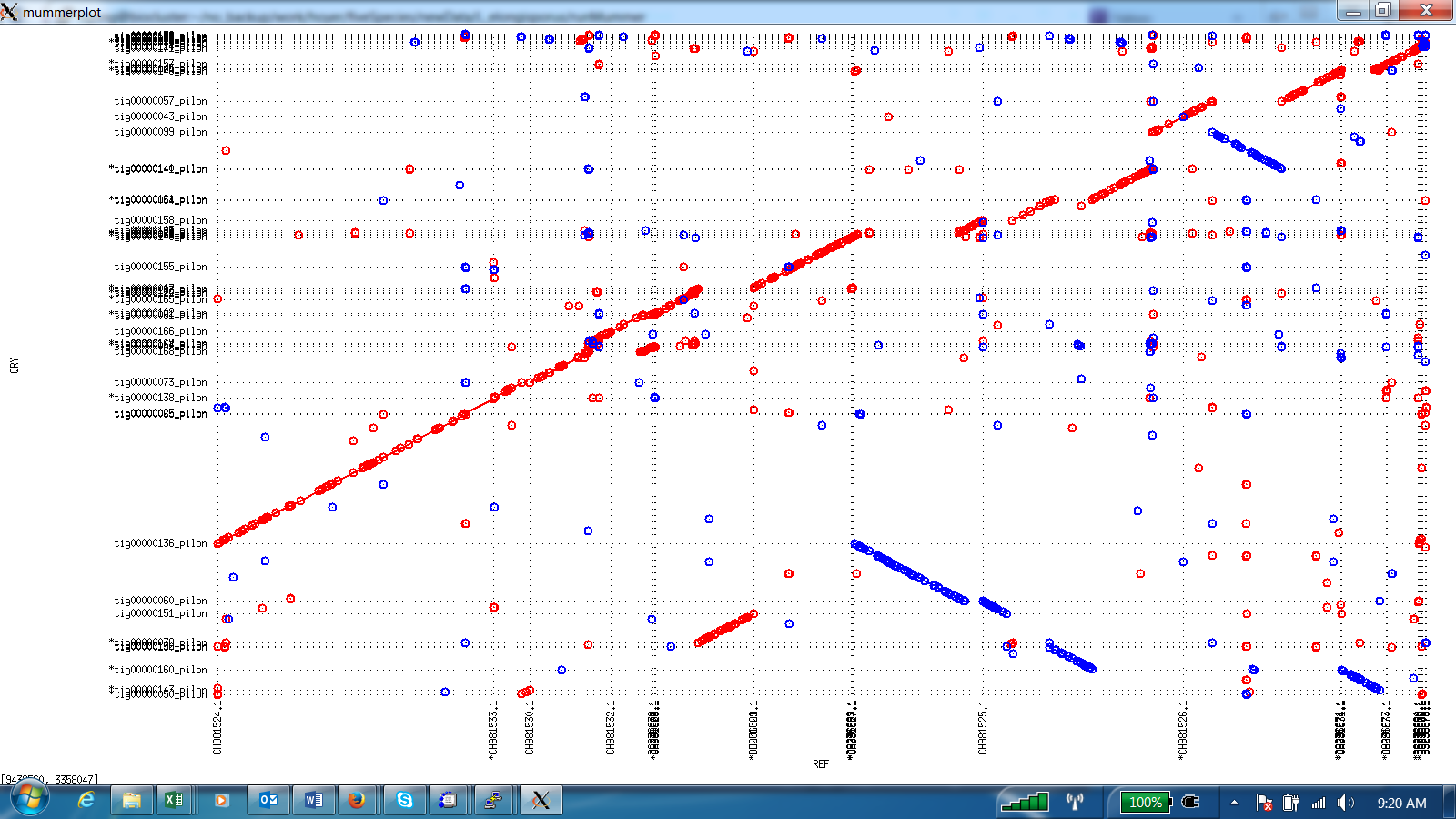


### Files Created:

**Raw ONT Data:**

L_elongisporus_raw.fasta 64,450 reads

L_elongisporus_raw.fastq 64,450 reads

**Clean ONT Data:**

L_elongisporus _trimmed.fastq 62,020 reads

**Clean Illumina Data:**

trimmed_Lodderomyces_elongisporus_CCGTCCCG_L001_R1_001.fastq 3,063,257 reads

trimmed_Lodderomyces_elongisporus_CCGTCCCG_L001_R2_001.fastq 3,063,257 reads

**Final Assembly:**

L_elongisporusCanuAsmPolishedPilon.fasta 53 contigs

**Compressed file with all the above files:**

lhoyer / L_elongisporus / L_elongisporus.tgz
