# Supplementary material for: Using Genomics to Shape the Definition of the Agglutinin-Like Sequence (ALS) Family in the Saccharomycetales
Source: Front Cell Infect Microbiol. 2021 Dec 14;11:794529. doi: 10.3389/fcimb.2021.794529 (PMC8712946; doi:10.3389/fcimb.2021.794529)
Supplement: Supplementary file 2 [file DataSheet_2.docx]

**SUPPLEMENTARY FILE S2 |** Details for assembly of *Meyerozyma guilliermondii* ATCC 6260 genome sequence data.

**Assembly of *M. guilliermondii***


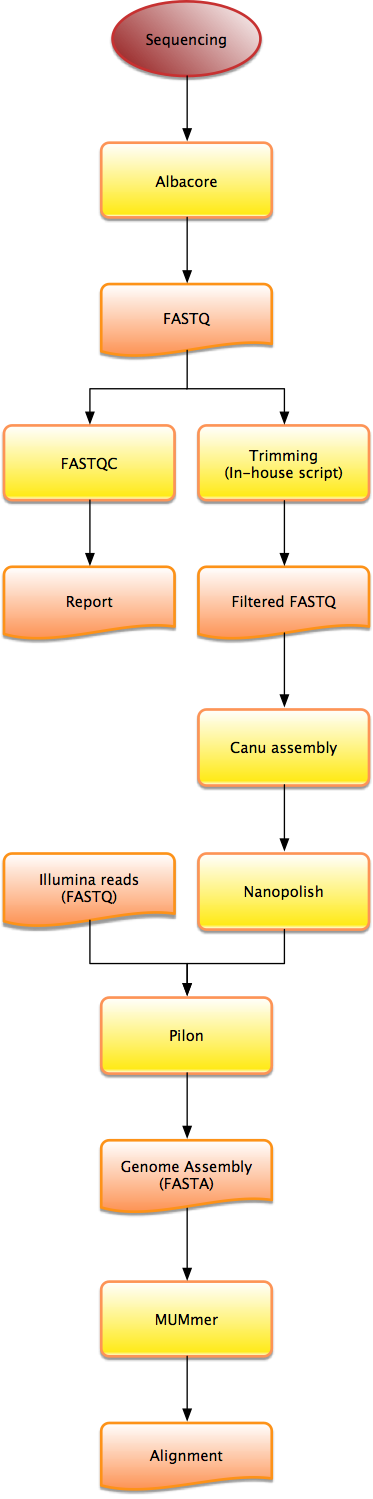


## Description of Library prep and sequencing.

1 ug of genomic DNA were sheared in a gTube (Covaris, Woburn, MA) for 1 min at 6,000 rpm in an Eppendorf MiniSpin plus microcentrifuge (Eppendorf, Hauppauge, NY).

The sheared DNA was converted into a Nanopore library with the Nanopore Sequencing kit SQK-LSK108 and the Native 1D Barcoding kit EXP-NBD103 (Oxford Nanopore)

The 5 libraries were pooled in equimolar concentration and the pool was sequenced on two SpotON MK I R9.5 flowcells for 24 h, using a MinION MK 1B sequencer. Basecalling was done in real time with software MinKNOW version 1.7.7.

## Data processing steps

### Albacore

ONT Albacore Sequencing Pipeline Software version 1.2.4 was used to demultiplex fast5 files from MinKNOW 1.7.7 basecalling program. The albacore command is:

"read_fast5_basecaller.py -i pass -t 14 -s newAlbacoreResults -k SQK-LSK108 -f FLO-MIN107 --barcoding --recursive -o fast5,fastq"

The following table shows the sequence counts and lengths for *M_guilliermondii* nanopore reads*.*

| Total bases: | 426,097,889 |
| --- | --- |
| Number of reads: | 43,266 |
| Mean read length (bp): | 9,848 |
| Median read length (bp): | 9,506 |
| Min read length (bp): | 160 |
| Max read length (bp): | 70,351 |

### FastQC

FastQC v-0.11.4 software was also used to further access quality scores and other attributes of the data sets. The following figures were selected from the FastQC reports.

#### FastQC results from M_guilliermondii_raw.fastq data


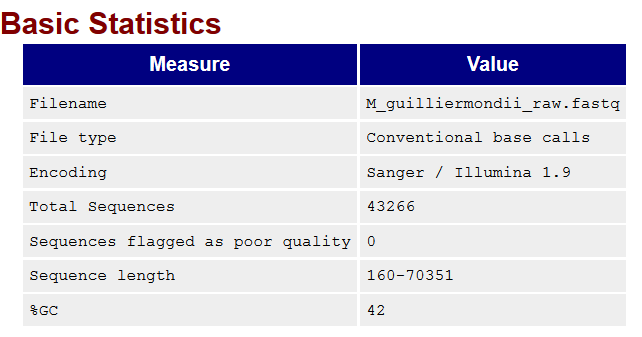


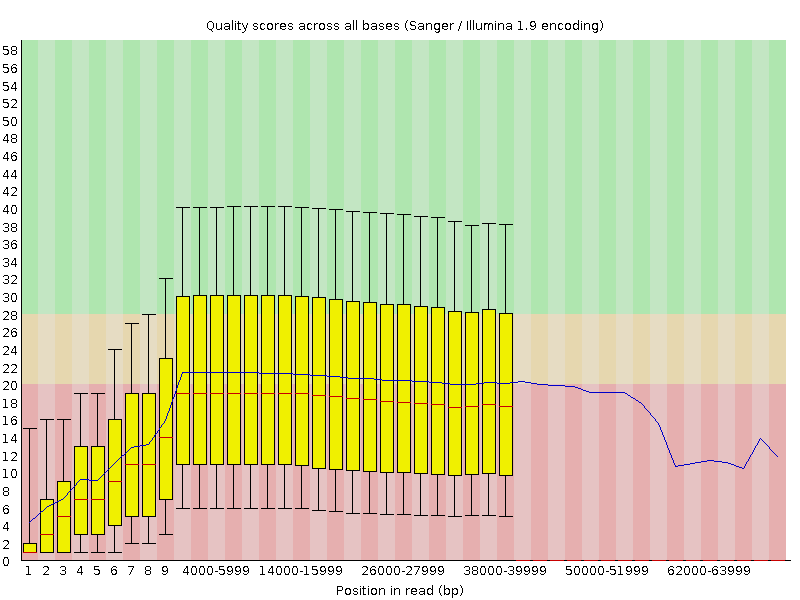


#### FastQC results from M_guilliermondii_trimmed.fastq data


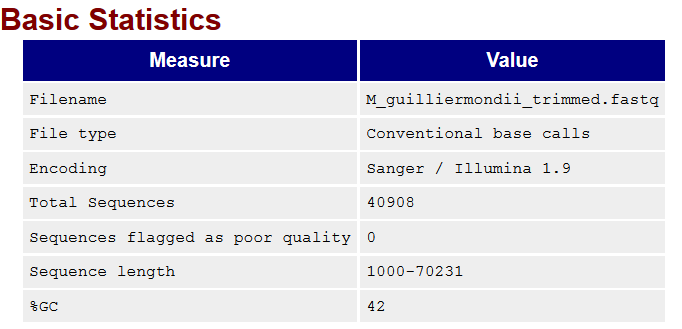


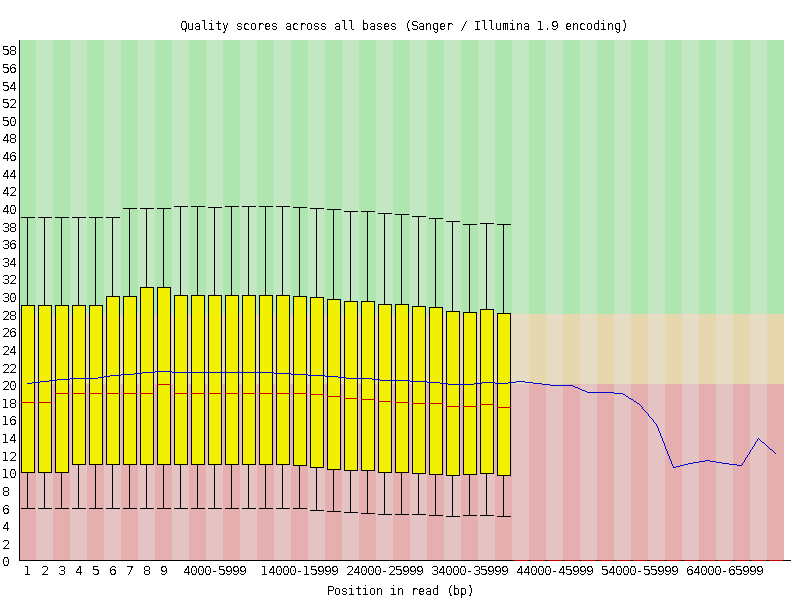


### Barcode trimming and low quality base removal

An in-house Perl script was used to trim 60 bases from the both ends of raw nanopore reads. Reads that are longer than 1,000 bps were selected for assembling purposes. For the Illumina MiSeq reads, Trimmomatic-0.36 was used to remove quality scores less than 30 as well as reads < 50 bp in length

### Canu-v1.5 Assembly

A total of 40,908 *M_guilliermondii* reads were used in Canu-v1.5. The assembly contains 9 contigs. The following table shows the contigs and their lengths

| **M_guilliermondiiCanuAsm(9 Contigs)** | | |
| --- | --- | --- |
| Contig Name | Length | Reads in the contig |
| >tig00000004 | 2,084,973 | 5,791 |
| >tig00000001 | 2,017,825 | 5,510 |
| >tig00000007 | 1,712,924 | 4,721 |
| >tig00000014 | 1,249,136 | 3,559 |
| >tig00000017 | 1,015,533 | 2,698 |
| >tig00000019 | 887,496 | 2,504 |
| >tig00000011 | 746,158 | 2,474 |
| >tig00000006 | 484,065 | 1,358 |
| >tig00000023 | 435,472 | 1,204 |
| total | 10,633,582 |  |

#### Canu-v1.5 Commands:

module load Canu/1.5-IGB-gcc-4.9.4-Perl-5.24.1

module load Java/1.8.0_121

module load gnuplot/5.0.6-IGB-gcc-4.9.4

canu -p asm -d defaultAsm genomeSize=10.6m useGrid=false \

-nanopore-raw M_guilliermondii_trimmed.fastq

### Nanopolish-v0.7.1

The consensus sequences from the assembly were added to Nanopolish v0.7.1 for error correction. Nanopolish uses the signal-level data from the nanopore with a novel hidden Markov model to achieve a more accurate assembly.

**Nanopolish commands:**

module load nanopolish/0.7.1-IGB-gcc-4.9.4-159d92b

module load parallel/20170622-IGB-gcc-4.9.4

module load BWA/0.7.15-IGB-gcc-4.9.4

module load SAMtools/1.5-IGB-gcc-4.9.4

nanopolish extract fast5 > reads.fasta

bwa index M_guilliermondiiCanuAsm.fasta

bwa mem -x ont2d -t 12 M_guilliermondiiCanuAsm.fasta reads.fasta | \

samtools sort -o reads.sorted.bam -T reads.tmp -

samtools index reads.sorted.bam

python nanopolish_makerange.py M_guilliermondiiCanuAsm.fasta | parallel \

--results nanopolish.results -P 12 \

nanopolish variants --consensus polished.{1}.fa -w {1} -r reads.fasta \

-b reads.sorted.bam -g M_guilliermondiiCanuAsm.fasta -t 4 \

--min-candidate-frequency 0.1

python nanopolish_merge.py polished.*.fa >M_guilliermondiiCanuAsmPolished.fasta

### PILON-v1.22

#### Reads from the Illumina paired end MiSeq run were trimmed with Trimmomatic v-0.36. The nucleotide bases with quality scores less than 30 were removed. Reads > 50 bp in length were selected. As a result, total 6,823,960 reads were used to align to the error corrected genome from Nanopolish with BWA v-0.7.15. The bam file from BWA and the corrected genome were used as input data for PILON software.

#### PILON-v1.22 Commands:

module load pilon/1.22-Java-1.8.0_121

module load BWA/0.7.15-IGB-gcc-4.9.4

module load SAMtools/1.5-IGB-gcc-4.9.4

# Index the draft genome

draftAsm= M_guilliermondiiCanuAsmPolished.fasta

bwa index ${draftAsm}

R1= trimmed_Meyerozyma_guilliermondii_ACTTGAAT_L001_R1_001.fastq

R2= trimmed_Meyerozyma_guilliermondii_ACTTGAAT_L001_R1_001.fastq.

# Align paired end reads to the draft assembly

bwa mem -x ont2d -t 12 $draftAsm $R1 $R2 | samtools sort -o pe_aln_sorted.bam -T reads.tmp -

samtools index pe_aln_sorted.bam

#run pilon

java -Xmx20G -jar pilon-1.22.jar --genome $draftAsm --bam pe_aln_sorted.bam --outdir out

1. **Mummer Plots**

**STATS OF FINAL ASSEMBLY**

| **Total bases:** | 10,700,359 |
| --- | --- |
| **Number of contigs:** | 9 |
| **N50 contig length (bp):** | 1,721,375 |
| **Mean contig length:** | 1,188,928 |
| **Median contig length:** | 1,023,586 |
| **Min contig length (bp):** | 439,088 |
| **Max contig length (bp):** | 2,095,026 |

### Comparison to NCBI reference genome

Nucleotide level comparisons between *M. guilliermondii* reference genome from NCBI (GCA_000149425.1_ASM14942v1_genomic.fna) and the newly constructed assembly were done with the dnadiff program from MUMmer v-3.23.

#### dnadiff Results:

|  | GCA_000149425.1_ASM14942v1 | M_guilliermondiiCanuAsmPolishedPilon |
| --- | --- | --- |
| TotalSeqs | 9 | 9 |
| AlignedSeqs | 9(100.00%) | 9(100.00%) |
| AlignedSeqs | 9(100.00%) | 9(100.00%) |
| TotalBases | 10,609,954 | 10,700,359 |
| AlignedBases | 10,548,139(99.42%) | 10,642,183(99.46%) |
| UnalignedBases | 61,815(0.58%) | 58,176(0.54%) |
| TotalLength | 10,716,969 | 10,714,766 |
| AvgLength | 106,108.60 | 106,086.79 |
| AvgIdentity | 99.95 | 99.95 |
| Insertions | 88 | 141 |
| InsertionSum | 64,221 | 132,225 |
| InsertionAvg | 729.78 | 937.77 |
| TotalSNPs | 912 | 912 |
| TotalGSNPs | 192 | 192 |
| TotalIndels | 3,62 | 3,624 |
| TotalGIndels | 1,123 | 1,123 |

Mummerplot from MUMmer was used to create a graphical representation of the alignments for the newly constructed assembly and GCA_000149425.1_ASM14942v1genome from NCBI.

#### MUMmer-3.23 commands:

module load MUMmer

# create report

dnadiff GCA_000149425.1_ASM14942v1_genomic.fna\

M_guilliermondiiCanuAsmPolishedPilon.fasta

# create plot

nucmer -maxmatch GCA_000149425.1_ASM14942v1_genomic.fna\

M_guilliermondiiCanuAsmPolishedPilon.fasta

delta-filter -m out.delta > out.delta.m

mummerplot –large –layout out.delta.m

#### **Mummer Plot:** **GCA_000149425.1_ASM14942v1_genomic.fna vs M_guilliermondiiCanuAsmPolishedPilon.fasta**


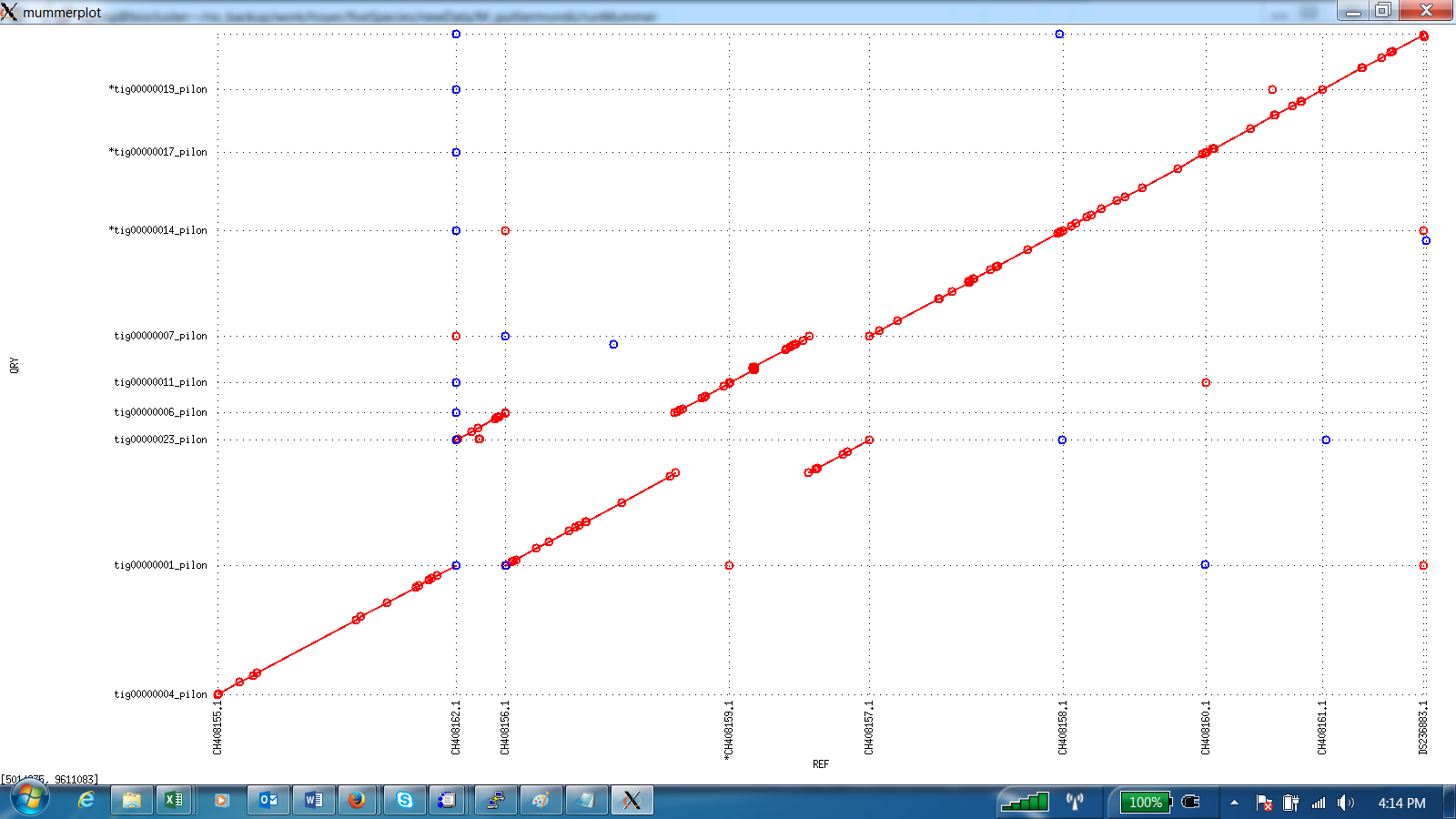


### Files Created:

**Raw ONT Data:**

M_guilliermondii_raw.fasta 43,266 reads

M_guilliermondii_raw.fastq 43,266 reads

**Clean ONT Data:**

M_guilliermondii _trimmed.fastq 40,908 reads

**Clean Illumina Data:**

trimmed_Meyerozyma_guilliermondii_ACTTGAAT_L001_R1_001.fastq 3,411,980 reads

trimmed_Meyerozyma_guilliermondii_ACTTGAAT_L001_R2_001.fastq 3,411,980 reads

**Final Assembly:**

M_guilliermondiiCanuAsmPolishedPilon.fasta 9 contigs

**Compressed file with all the above files:**

lhoyer / M_guilliermondii / M_guilliermondii.tgz
