# Supplementary material for: Using Genomics to Shape the Definition of the Agglutinin-Like Sequence (ALS) Family in the Saccharomycetales
Source: Front Cell Infect Microbiol. 2021 Dec 14;11:794529. doi: 10.3389/fcimb.2021.794529 (PMC8712946; doi:10.3389/fcimb.2021.794529)
Supplement: Supplementary file 5 [file DataSheet_5.docx]

**SUPPLEMENTARY FILE S5 |** Amino acid sequences used to draw the phylogenetic tree in **Figure 5**. Sequences were trimmed, based on Clustal Omega alignments with *C. albicans* NT-Als3 4LE8, to produce inputs for AlphaFold structural predictions that lacked the secretory signal peptide and amyloid-forming region.

**>CaAls1**

MLQQFTLLFLYLSIASAKTITGVFDSFNSLTWSNAANYAFKGPGYPTWNAVLGWSLDGTSANPGDTFTLNMPCVFKYTTSQTSVDLTADGVKYATCQFYSGEEFTTFSTLTCTVNDALKSSIKAFGTVTLPIAFNVGGTGSSTDLEDSKCFTAGTNTVTFNDGDKDISIDVEFEKSTVDPSAYLYASRVMPSLNKVTTLFVAPQCENGYTSGTMGFSSSNGDVAIDCSNIHIGITKGLNDWNYPVSSESFSYTKTCTSNGIQIKYQNVPAGYRPFIDAYISATDVNQYTLAYTNDYTCAGSRSQSKPFTLRWTGYKNSDAGSNGIVIVAT

**>CaAls2**

MLLQFLLLSLCVSVATAKVITGVFNSFDSLTWTRAGNYAYKGPNRPTWNAVLGWSLDGTSANPGDTFTLNMPCVFKFITDQTSVDLTAEGVKYATCQFYSGEEFTTFSSLKCTVSNTLTSSIKALGTVTLPISFNVGGTGSSVDLESSQCFKAGTNTVTFNDGDKKISIDVDFEKTNEDASGYFIASRLIPSINKVSITYVAPQCANGYTSGAMGFIVLTGDTTIDCSNVHVGITKGLNDWNFPVSSDSLSYNKTCSSTGISITYENVPAGYRPFFDVYTSVSGQNRQLRYTNDYACVGSSLQSKPFNLRLRGYNNSEANSNGFVIVAT

**>CaAls3**

MLQQYTLLLIYLSVATAKTITGVFNSFNSLTWSNAATYNYKGPGTPTWNAVLGWSLDGTSASPGDTFTLNMPCVFKFTTSQTSVDLTAHGVKYATCQFQAGEEFMTFSTLTCTVSNTLTPSIKALGTVTLPLAFNVGGTGSSVDLEDSKCFTAGTNTVTFNDGGKKISINVDFERSNVDPKGYLTDSRVIPSLNKVSTLFVAPQCANGYTSGTMGFANTYGDVQIDCSNIHVGITKGLNDWNYPVSSESFSYTKTCSSNGIFITYKNVPAGYRPFVDAYISATDVNSYTLSYANEYTCAGGYWQRAPFTLRWTGYRNSDAGSNGIVIVAT

**>CaAls4**

MLLQFLLLSLCVSVATAKVITGVFNSFNSLTWANAASYPYRGPATPTWTAVIGWSLDGATASAGDTFTLDMPCVFKFITDQTSIDLVADGRTYATCNLNSAEEFTTFSSVSCTVTTTMTADTKAIGTVTLPFSFSVGGSGSDVDLANSQCFTAGINTVTFNDGDTSISTTVDFEKSTVASSDRILLSRILPSLSQAVNLFLPQECANGYTSGTMGFSTAGTGATIDCSTVHVGISNGLNDWNYPISSESFSYTKTCTSTSVLVTFQNVPAGYRPFVDAYISATRVSSYTMQYTNIYACVGAASVDDSFTHTWRGYSNSQAGSNGITIVVT

**>CaAls5**

MIQQFTLLFLYLSFATAKAITGIFNSIDSLTWSNAGNYAFKGPGYPTWNAVLGWSLDGTSANPGDTFILNMPCVFKFTASQKSVDLTADGVKYATCQFYSGEEFTTFSSLKCTVNNNLRSSIKALGTVTLPIAFNVGGTGSSVDLEDSKCFTAGTNTVTFNDGSKKLSIAVNFEKSTVDQSGYLTTSRFMPSLNKIATLYVAPQCENGYTSGTMGFSTSYGDVAIDCSNVHIGISKGVNDWNHPVTSESFSYTKSCSSFGISITYQNVPAGYRPFIDAYISPSDNNQYQLSYKNDYTCVDDYWQHAPFTLKWTGYKNSDAGSNGIVIVAT

**>CaAls6**

MKTVILLHLFFYCTIAMAKTISGVFTSFNSLTYTNTGNYPYGGPGYPTWTAVLGWSLDGTLASPGDTFTLVMPCVFKFITTQTSVDLTANGVKYATCTFHAGEDFTTFSSMSCVVNNGLSSNIRAFGTVRLPISFNVGGTGSSVNIQDSKCFTAGTNTVTFTDGDHKISTTVNFPKTPQSSSSLVYFARVIPSLDKLSSLVVASQCTAGYASGVLGFSATKDDVTIDCSTIHVGITNGLNSWNMPVSSESFSYTKTCTPNSFIITYENVPAGYRPFIDSYVKKSATATNGFNLNYTNIYNCMDGKKGNDPLIYFWTSYTNSDAGSNGAAVVVT

**>CaAls7**

MKKLYLLYLLASFTTVISKEVTGVFNQFNSLIWSYTYRARYEEISTLTANAQLEWALDGTIASPGDTFTLVMPCVYKFMTYETSVQLTANSIAYATCDFDAGEDTKSFSSLKCTVTDELTEDTSVFGSVILPIAFNVGGSGSKSTITDSKCFSSGYNTVTFFDGNNQLSTTANFLPRRELAFGLVVSQRLSMSLDTMTNFVMSTPCFMGYQSGKLGFTSNDDDFEIDCSSIHVGITNEINDWSMPVSSVPFDHTIRCTSRALYIEFKTIPAGYRPFVDAIVQIPTTEPFFVKYTNEFACVNGIYTSIPFTSFFSQPILYDEALAIGADLVRT

**>CaAls9-1**

MLPQFLLLLLYLTVSTAKTITGVFNSFNSLTWANAANYGYQTPETPTWTAVLGWSLNSTTADAGDTFTLIMPCVFKFITSQTSVDLTADGVSYATCDFNAGEEFTTFSSLSCTVNSVSVSYDKASGTVKLPFSFNVGGTGSSVDLTDSKCFTAGKNTVTFTDGDTEISTSVDFQASPISSSGYIASARVVPSLNKASSLFVSPQCENGYTSGIMGFVTSQGATIDCSNINIGISKGLNDWNFPVSSESFTYTKTCSSSGIIVEYENVPAGYRPFVDAYISSENVEQYTLTYANEYTCKNGNTVVDPFTLTWTGYKNSEADSNGDIIVVTT

**>CaAls9-2**

MLPQFILLFISLTVSTAKTITGVFNSFDSLTWTRSVEYAYKGPETPTWNAVLGWSLNSTTADPGDTFTLILPCVFKFITTQTSVDLTADGVSYATCDFNAGEEFTTFSSLSCTVNSVSVSYARVSGTVKLPITFNVGGTGSSVDLADSKCFTAGKNTVTFMDGDTKISTTVDFDASPVSPSGYITSSRIIPSLNKLSSLFVVPQCENGYTSGIMGFVASNGATIDCSNVNIGISKGLNDWNFPVSSESFSYTKTCTSTSITVEFQNVPAGYRPFVDAYISAENIDKYTLTYANEYTCENGNTVVDPFTLTWWGYKNSEADSDGDVIVVTT

**>CdAls64210**

MLQQFILLFVYLSVATAKTITGVFNSFDSLTWSNAANYAFKGPGYPTWNAVLGWSLDGTTADPGDTFTLIMPCVFKFITTQTSVDLTANGVKYATCSFYSGEEFTTFSSLTCTVNSALTSSVKAFGTVTLPISFNVGGTGSSVDLEDSKCFTAGTNTVTFTDGDNKVSTTVDFEKSTVDSTGYLTSSRLMPSLNKVTSLFVAPQCARGYTSGTIGFSSSNGGVSFDCSNVHVGITNGVNDWNFPVSSESFSYTKTCSSTGITITYRNVPAGYRPFIDAYITASDVNSYTLSYTNDYTCVGGSVQHKPFTLRWSGYKNGEAGSNGIVIVAT

**>CdAls64220**

MLQQFVLLFIYSTIATAKTITGVFNSFDSLTWTNSGNYGFKGPETPTWNAVLGWSLNSTIADPGDTFTLIMPCVFKFITTQTSVDLTVDGVNYATCSFYSGEEFTTFSSLTCTVNNVLTPYAMASGTVTLPISFNVGGSGSSVDLEDSKCFTAGTNTVTFNDGDTKISTTVDFDASPVASSGYITSSRIIPSLNKVSSLYVVPECESGYAFGVMGFVASNGATIDCSNVHIGISKGLNDWNFPVSSESFSYTKTCTSASITVEFQNVPAGYRPFVDTYISAENVGRYTLTYANDYTCNNGYSVVDPFTLTWWGYKNSEADSDGAIIVVTT

**>CdAls64610**

MFLQFLLLCICLSVATAKVVTGIFHSFDSLTWTNAASYPYRGPSTPTWNAVIGWTLDGAVASPGDTFTLNMPCVFKFITDQTSVDLVADGRTYATCDLYSGEEFTTFSSLKCTVSNALNSQTKALGTVTLPLSFNIGGSGSDVDITSSQCFKEGTNTVTFNDGDTTFSTTANFQRSDVNANDRILLSRILPSLAKSVTIFIPPRCASGYSSGTMGFSTAGTDAIIDCSTVHVGISNGLNDWNYPIESKSFSYTTSCSSKGVSVTYQNVPAGYRPFVDAYISALTSYTMQYTNQYTCVGARPVDASFSYNWLGYDNAEAGSRGITIVVTTSTVT

**>CdAls64800**

MFLRFLLLCICLSVAGAKVITGIFNSFDSLTWTNAARYPFKGPGYPTWNAVLGWSLDGTVASPGDTFTLIMPCVFKFITNQTSVDLIADGADYATCQFHAGEEFTTFSSLTCTVSSALNPSIKALGTVTLPISFNVGGTGSSVDLENSKCFTAGTNTVTFTDGNNKISTTVEFNKTTVDPSGYLTSARRIPSLNQVTSLYMAPQCVNGYTSGIMGISSNSGIDIDCSTVHVGISKGVNDWNFPVSYDSFSYKKTCSPTGISITYENIPAGYRPFIDAYISASGVKSYALSYANNYTCVDSSFQNKPFTIRWTGYNSSEAGSDGVVIVVTT

**>CdAls65010**

MFLRFLLLCICLSVAGAKVITGIFNSFDSLTWTNAARYPFKGPGYPTWNAVLGWSLDGTVASPGDTFTLIMPCVFKFITNQTSVDLIADGADYATCQFHAGEEFTTFSSLTCTVSSALNPSIKALGTVTLPISFNVGGTGSSVDLENSKCFTAGTNTVTFTDGNNKISTTVEFNKTTVDPSGYLTSARRIPSLNQVTSLYMAPQCVNGYTSGIMGISSNSGIDIDCSTVHVGISKGVNDWNFPVSYDSFSYKKTCSPTGISITYENIPAGYRPFIDAYISASGVKSYALSYANNYTCVDSSFQNKPFTIRWTGYNSSEAGSDGVVIVVTT

**>CdAls86150**

MNKLYLPYLLALITTVISKEITGVFTKFDSLIWSFTYRARYEEIPTLTANAQMEWVLDGTMASPDDTFTLVMPCVYKFMTYQSSVQLTSNSIPYATCNFDAGEDSKTFSSLKCTVTNELTENTNVFGDIILPIVFNVGGSGSESTLKDSKCFSGGYNSVTFFDGDNQLSTTAYFAPRRDLAYGLVVGQRLFASLDKMTNLVMSTPCLTGYQSGKLGFSFADDDFEIDCSSIHVGITNEINDWSMPVSSVPFDHTRSCTSRALYIEFQEIPAGYRPFVDAIVQIPTTKPFYVQYTNEFTCVNGLFTSLPFTSFFSQSILYDKALVNGADLV

**>CdAls86290**

MKGVVLLHLFFYCAIATAKTISGVFTSFNSLTYANTGNYPYGGPGYPTWTAVLGWSLDGTVASPSDTFTLIMPCVFKFITTQTSVDLTANGVKYATCTFHAGEDFTAFSSMSCVVNNGLTSNIKAFGTVRIPISFNVGGTGSSVNLQDSKCFTAGTNSVTFTDGDHKISIPVDFPKTPESSSGLIKYSRVIPTLDKLSSLAVASQCTAGYKSGVLGFSATKNDVTIECSNVHVGITNGLNSWNMPVSSDSFSYTKTCTSSSFIITYENVPAGYRPFIDTYVKKTSTTSTGFNLNYTNSYVCTDGKKGNDPLIYFWTSYTNSDAGSDGAVVIVTT

**>CtrAls941**

MLLLQLVITLLTSVKAVLADEISGVFTSFDSLTFSHPRLTYTPPNFPTWTAVFGWDLEGSTARPGDVFTLVMPCVFKFLTSTPYVELTADGVTYATCRLNSGEEFVSFSSMECTVSENLTPSSIVYGQVSVPLTFNAGGSGSETDIEASTCFVVGENTVTFTDGDNSLSIQVNFEANPADPSGLLSSQRVIQSLAKSLALVIIPDCPNGYASGTLGISSTADGYQLDCNSIEAGLTSGLNAWNNPIDNIDFPHTSQCTTKGFSISFSNIPAGYRPFINALATVPSTEQYRVAYEVKYTCVGGSYRDDSMTRLWNPYQRSEADSYGQPIEIIT

**>CtrAls1028**

MKFLGLVLLFLSLINQVTPKEVSGIFTSFNSLTWSDAGNYGYRGPANPAWQAKLSWSLEGKKVNPGDTFTLTMPCVFKFVTTQPSIDLAANGITYATCTFHSGEEFTTFSTVSCIVSDALTSSTQAFGTVSIPFSFNIGGSGSDVDLTDSTCFTTGSNTVTFKDGDNELSIQTNFEQTKDSQSGLITNARVIPSLGQLSHLVVAPDCPNGYASGELGIYARDNSVTINCENIHIGITDKLNAWNNPTNSNGFTYTKKCDSNGFSISFKNVPTGYRPFLDSLINAATDYTFTISYISKYTCATGDYHDKSITKNWAPYKNGLADSDGAVVFVTT

**>CtrAls1030**

MFVFRLYLLLLVFLSEVTPKELSDVFTSFNSLTFTDAGYGYRGPSNPTWHAKLSWNLNGAYARPGDTFGLVLPHVFKFVTAQSYFTLSAGGVTYAICDFQPGELFTTFSSIKCTVSEKLNPNIEAFGTITFPFAFGVGGSGSDTDLVNSNSFTTGENRVTFKHGSKDLCIDVDFQGSPAKTTDLLSYGRIIPSLRKISHLLTSADSPNGYKSGKLGLASSDAGLGIDCDSVHVGITNMLNPWNQPMNAESFSYTTQCSEEEIMITFNEVPEGYRPFFDVLFSHTASDIFTMLYTNEYVGADGVTYDASMKKAWKSYQDSLPSGDGAIIIVT

**>CtrAls1038**

MKLIGFGLLLSSLATLVAPKEISGVFTSFDSLKWNEDSNDFRGPASPTWKATLGWSLDGTKVNPGDTFTLIMPCVFKFITEQTTIDLTANGVNYATCTFHAGEEFTAFSSVGCVVKDALKSNIQAFGTVTIPFTFNVGGTGTSVSLQDSTCFTTGDNIVTFQDGDNKLSITANFEPTGASRSDLIANARSIPSLEKMTHLVIAPDCPSGFKSGTISLNTNNGADIDCAQVHVGMTNFINPWNYPTNSEQNFSKQPTCNEGSFTLSFESVPAGFRPFFDVMVTPKGKMGFDYKYSVVCADGESLEHPTHYDWGTYNTQTADSNGAILVIT

**>CtrAls1041**

MSYFGFAILLLALFTRVTPKEITGIFTSFDSLTYSDAGNYGYQGPGNPTWTATLGWSLDGSVASPGDTFTLIMPCVFKFTSSSTSVDLTADGVTYATCKLNNGEEFTTFSSMSCVVNSALTSDTQAFGTVTVPFSFNVGGTGSSVDLEDSTCFTAGTNTVTFKDGDNELSINAVFEKTTASVSDEIIFVRSVPSIGKLQQISIAKDCPSGYESGYMSIIIRDNSAVMDCSSTHIGITNDLNDWNQPTNSETFSYTESCSATNFTISFTDIEAGYRPFMDSFLTATANARFNVDYIYKYTCKNGDTVDKTNSRVYAPYINSNTDSNGAILVIT

**>CtrAls2228**

MVLIQAIVLLIWIQLVSSKEISGIFIGFDSLTWNAASDLPSAYQGPQIPTWTAELTWFLNGESAEPGDTFTLIMPCVFKFITNQNTVDLIADGTTYATCNLNSGEEFTTFSSLSCTVSSTLTTYTQARGTLHVPLTFNVGSSGTSVSLTDSTCFRPGVNTVTFRDGDNEISTQATFQGSPDDPSPDRYYQRVIPSLNKVSNLVYPPNCPNGYSSGVISFSSSDSNFQLDCSSLDVGMTNQLNAWNFPTSRDSLSYTRSCSDKSITVNFQNVPAGSRPYISVLSAFLGTKTYSINYNLRYTCSGSRSSSVTKTISWAPYRNSLADSNGAVVVYTTSTVT

**>CtrAls2229**

MLRLSITYSIFTLINVINAKVLSGIFTRFVSLTQSSYNSYSFDGPMSTTWIAALGWEINGTKAKPGDTFTLEMPCVYKIFINEESIDLIGNDISLATCEVHSGEHISTNSYLNCVMNDSLDERTNIDGILRLPIMFNVGGSGQDTDLDAASSCFNTDSNIIAFNNGDTSISIQRSFSVRAIFDTVPINFVRVGKTISELEVLIVAPNCPQGYTNGRLAIAASDRDVIIKCSTITSGFASKLNKWNLPENLVQLYHGSLCTSRQFSISYTNIPEGYRPFLSVTLSNAVSSSFNLRYTIQNTCEKDTFNDQSRSVSWRKLNYGSIDSYGGIMIPVT

**>CtrAls2293**

MFLLQAVILYCSFIVTAVAKEISGVFTGFESLTWDKAANYGFQGPQYPTWNAVLDWSLDGTTTSPGDTFTLIMPCVFKFTTSATSIDLRANGITYATCDLHAGEEFTTYSSLTCTVTDSLSSVHEASGTVTIPLAFNVGGSGSSVDIADSTCFTAGTNTVTFQDGDTSISTQAYFAAATESSSGLLYFQRSVPSLNKLNALAILPDCPNGYTSGTLGFSSSNSRFSIDCSSAEAYITNLLNSWNYPTSADSFSYTQTCTSKSFQITFNNIPAGYRPYIAALVQAPSSDYAIQYTAKYRCEGSVQRDDSQKISWAGYTNSDPDSNGAVVVLTTIT

**>CtrAls3786**

MIFSEFLILSLTFIATSVAKEISGVFIGFESLTWDKAGDYAYQGPQYPTWNAVLDWSLDGTTTSPGDTFTLIMPCVFKFTTSATSVDLTANGITYATCDLHAGEEFTTYSSLTCTVTDSLSSVHEAMGTVTIPLAFNVGGSGSSVDIADSTCFTAGTNTVTFQDGDTSISTQAYFAAATGSSSDLLYFQRVVPSLNKLNALVILPDCPNGYTSGTLGFSSSNSRFSIDCSSAEAYITNLLNSWNKPTTADSFTYTQTCTSKSFQITFNNIPAGYRPYIAALVQAPSSDYAIQYTAKYQCTGSTQKDITKSVTWSGYTNSDTDSNGAVVVLTTIT

**>CtrAls3791**

MFLLQTTLLCCALIATSVAKEISGVFIGFESLTWDKAGNYAYQGPQYPTWNAVLDWSLDGTTTSPGDTFTLIMPCVFKFTTSATSVDLTANGITYATCDLHAGEEFTTYSSLTCTVTDSLSSVHEAMGTVTIPLAFNVGGSGSSVDIADSTCFTAGTNTVTFQDGDTSISTQAYFAAATGSSSGLLYFQRSVPSLNKLNALAILPDCPNGYTSGTLGFSSSNSRFSIDCSSAEAYITNLLNSWNYPTSADSFSYTQTCTSKSFQITFNNIPAGYRPYIAALVQAPSSDYKIDYTAKYQCAGSSQKDASKSVTWSGYTNSDTDSNGAVVVLTTIT

**>CtrAls3797**

MNFVSLLFTLLLLVTRATSKTLTGVFQSFNSLTWEKAASYKYRGPQFPTWNAAVNWALDSNANAGDTFTLIMPCVFKFTTSETSIDLTVGSKSYATCNFNAGEHFTTFSSLSCTVTQSVPDNTNAYGTITVPLAFNVGGSGRDVDTTDAKCFTTGDNTVTFSDGDKSFSTTANFEGAGTLNDDYESSRLIPSLGKTDALLVAPLCSNGYKSGTIGFSSKASGYSIDCNNIQAGITSQLNAWGFPTDSQSFSYTTQCTTTSYSITFSTIPKGLRPFIDAYIKTPTSTYAVTYTFKYVCADGKSYNSNRSLNWSGYVNGDADSEGMEIVVAT

**>CtrAls3871**

MNLITFILLLSSLITIVTPKEITGVFTSFNSLTYNDAANYGAQCPGYPTWIATLGWSLDGSVASPGDSFTLIMPCVFKFTSSETSVDLTVDGVSYATCNLNNGEEFTTFSSMSCVVSSALTSTTQALGAVSIPFSFNVGGSGSSVDLEDATCFTAGTNTVTFKDGDNELSINAVFDKTTASVSDEIISVRSVPSIGKLQQISIAKDCPSGYGSGYMSIIIKDNTAVMDCSSVHIGITNELNDWNQPMNSESFSYTKSCSATEFIVSFTDIAAGYRPFMDSFLTTTGNAKLTVDYHYEYTCKNGDTVAETDRRIFSPYTNSNTGCSGVVLVIT

**>CtrAls3882-1**

MKFITFGLLLSSLLTLVTPKEVTGIFTSFDSLTWNEQTTPFSSPASPTWRATLGWSLDGTKLNQGDTFTLTMPCVFKFITDQTTIDLMANGVSYATCTFHAGEEFTTFSTVACVVNDALKSNMQVTGSVTIPFTFNVGGTGTSVSLEDSTCYTAGKNTVIFKDGDNELSTIANFEPTDASRTELITNARSIPSIKRTSHVIIAPDCPSGYKSGTITFDTNNGADIDCGQTHVGMTNFINPWNYPTNSEQNFSKQPTCTKGKYTLSFQDVPAGYRPFFDVLVKPTGKMTFYYNSDLVCADGTTYKKGLAWDWGSYQNDVADSSGDVIVITT

**>CtrAls3882-2**

MKFITIGLLVSALFTKVTPKEITGVFTSFNSLTYFDAGNYGYQGPGNPTWTSTLGWSLDGSVASPGDTFTLIMPCVFKFTSSSTSVDLTVDGVSYATCNLNNGEEFTTFSSMSCVVSSALTSTTQALGTVTVPFAFNIGGSGSSVDLEDATCFTSGTNTVTFKDGDNELSINAVFDKTTASVSDEIISVRSVPSIGKLQQLSIAKDCPSGYGSGYMSIIIKDNTAVMDCSSVHIGITNELNDWNQPMNSESFSYTKSCSATEFIVSFTDIAAGYRPFMDSFLTTTANAGFTVDYHYEYTCKNGDTITKTNSRVYSPYINGNTDSNGAILVIT

**>CpAls4770**

MIKQLSFASAFIAFVLTTFVQATDISNVFQSFDSLTWQNGANYRYRTPAAPSWIAQLSWHILGSNVKPGDTFTLNMPCVFKFTTSQKTVDLKVGGTVYATCQFAPGDLVVAYSQLKCTASDNVKDSTDATGTVRFPFTFNVGGSAGAVDLQNSKCFTPGTNEVTFTDGDKKLTVIANFQGGSNTNTGSTEDIVYSNRLVPTLNKQQLYLLGGTCPNGYRRGTLGITTVGGTFDCSSIHSAITNNLNEWFLPEVVETILATSRCNGQSYTINYDNIPAGYRPFIDILISRPVGQVLRTTYTNRFQCAGSLTTTDRSLSVTWAEYRNNEAGANGNEVVVTTSTWL

**>CpAls4780**

MVKHLQFVTILVAFTLTALTQAAEISNVFQSFDSLTWENGASYRYRTPAAPSWIAQLSWKILGSNVKPGDTFTLNMPCVFKFTTTQQSVDLDVGDTVYATCRFEPGDLVVAYSQLKCTASDNVKDSTDATGTVRFPFTFNVGGSAGVVDLQNSKCFTPGTNEVTFTDGDKKLTTTANFQGGSNTNPGTSTNNIVFNNRVVPSLNKQQLYLLGGTCPNGYRSGTLGITVSGGTLDCSSIHSAITNKLNGWFLPEVAEAISASTNCNGQSYTINYNNIPGGYRPFLDVLVQRPTGQVLRTNYINRYQCAGEIFVRDNSQSIKWGEYRNNEAGANGNEVVVTTSTWL

**>CpAls4790**

MTRMHIWAQLLILLYSLTLTTAAQVSGIFTSFNSLTWKSASNYRNAAPNYPTWTAVLGWSLDGAKVNPGDTFTLTMPCVFKFITTQTSVDLSADGVSYATCQLNPGEILVTYSTLTCTVNSALRSNMEATGTLSLPLSFNVGGSGGNADVADASCFKVGQNTVTFTDGSNSISTTANFKQGDYQLGTYDKFINYRLIPSLNEAQHYMVSGPCAKGYVSGTIGLATSDSGSIDCSNWHVGYSNDFNEWAFPKSFSSDYTVTSSCSSSQLLVSFKNVPAGYRPFIDALFRVPNGAGVKVTYINTASCVADTKQQNWGEEGYGWGSYQNGEAGANGIIVVLTTSTIL

**>CpAls4800**

MVKHLQFVAILVAFTLTALTQAAEISNVFQSFDSLTWENGASYRYRTPLTPSWIAQLSWKILGSNVKPGDTFTLNMPCVFKFTTTQESIDLDVGDTVYATCRFEPGDLVVAYSQLKCTASDNVKDSTDATGTVRFPFTFNVGGSAGVVDLQNSKCFTPGTNEVTFTDGDKKLTTTANFQGGSNTNTDNTPTDDIVYSNRVVPSLNKQQLYLLGGTCSNGYRSGTLGITVSGGTLDCSALHSAITNKLNGWFFPEVADAISASSSCNGQSYTINYDNIPAGYRPFIDILVSVPNGQKLRTTYTNSYKCVGEQRARDKSKVVTWGAYNNNVAGANGNEVILTTSTWL

**>CpAls660**

MKNWNSFVAIFTAFTFTTLTLASEVTDVFQSFDSLVWQNAANYQYSTPAAPSWIATLSWKILGSNVHAGDTFTLNMPCVFKFTTTQDSVDLTVGDTVYATCQFAPGDLVVAYSQLKCTASNNVKASTNAAGSVHFPIAFNVGGSANSVDLQNSQCFTAGSNQVTFTDGDKELTTTANFQGGTNTNGNSPTDTIVYNNRVVPSLNKQQLYLLGGTCPNGYRSGTLGVTIVGGTLDCSTFHASITNQLNDWFLPEEAEAISGSTSCSGNSFTINYNNIPAGYRPFLDILVSVPTGQGLQTAYTNTYLCAGSSSVKDGSKSTTWGPYQNNDAGANGNEVVVTTSTWL

**>CoAls4210**

MVKHLSFAAIFVAFALTTLTQAAEISNVFQSFDSLTWENGANYQYRIPAAPSWIATLSWKILGSNVHPGDTFTLNMPCVFKFTTTQESVDLDVGGTVYATCQFKPGDLVVPYSQLKCTASNNVKGSTDATGTVRFPLTFNVGGSANSVDLQDSKCFTPGTNDVTFTDGDKELTTTANFQGGTNTNRGIPYDDIVFNNRVVPSLNKQQLYLLGGTCPNGYRSGTLGITVSGGTLDCSSLHAAITNQLNGWYNPEVAEAISASSSCNGRSYTINYNNIPAGYRPFMDILVARPAGQQLRTSYTNTYQCAGQLFPRDNSRSIRWGAYNNNEAGANGNEVVVTTSTWL

**>CoAls4220**

MVKHLSFAAIFVAFALTTLTQAAEISNVFQSFDSLTWENGANYQYRTPAAPSWIATLSWKILGSNVHPGDTFTLNMPCVFKFTTTQESVDLDVGGTVYATCQFEPGDVVVAYSQLKCTASNNVKDSTDATGSVKFPFTFNVGGSAGDVDLQDSKCFTAGTNQVTFTDGDKELTTTANFQGGSNTNSGSTDDIVFNNRVVPSLNKQQMYLLGGTCPNGYRSGTLGITVSGGTLDCSSLHAAITNQLNAWYFPEVADSISASSSCNGQSYTINYNNIPAGYRPFMDILVAVPNGQRLKASYTNRYQCADEVGSHDNSKSITWSPYSNNVAGANGNEVVVTTSTY

**>CoAls800**

MKNWSSLVAIFTAFTLTTLTFAAEVTDVFQSFDSLTWENGATYQYSTPAAPSWIATLSWKVLGSNVHAGDTFTLNMPCVFKFTTTQDSVDLNVGDTVYATCQFAPGDLVVAYSQLKCTASNNVKDSTNAAGSVHFPIAFNVGGSAGEVDLQDSKCFTAGTNQVTFTDGDKKLTTTANFQGGSNTNGGISTDTIVYNNRVVPSLNKQQLYLLGGTCDNGYRSGTLGITVSGGTLDCSTLHASITDQLNAWFLPEEAEAISASTSCNEGSYTINYSNIPAGYRPFLDILVSVPTGQSLQTSYTNTYLCAGDSFPNDGSRSVTWSPYNNNDAGANGNEVVVTT

**>CmAls4210-1**

MAKHLSIVAIYFVLALTAITQAAEITNVFQSFDSLIWENGANYRYRTPSAPSWIATLSWKILGSNVHAGDTFTLNMPCVFKFTTTQQSVDLDVGNTVYATCQFLPGDLVVPYSQLKCTVSNNVKANTDATGTVRFPITFNVGGSGGSVDLENSQCFTPGTNDVTFTDGDKNLTTTANFQGGTNTNSGISYDDIVFNDRVVPSLNKEQLYLLGGTCPNGYRSGTLGITVNSGGTLDCSTIHSAITNQLNAWYFPEAAEAISASSSCNGRSYTINYGAIPAGFRPFIDILVSRPTGQQLRTTYTNRYQCAGQLFPRDNSRLVSWGAYNNNEAGANGNEVIVTTSTWL

**>CmAls4220-1**

MMRMHIWTQLSIFLHIFTLTTAAQVSGIFNSFNSLTWKAASTGYINASPNNPTWTAVLGWSLDGSKQAPGDTFTLMMPCVFKFITSQTSVDLTADGVSYATCQMNPGEILVTYSTLTCTVKSALQSNVQATGSLSIPLSFNIGGSGGNADVIDALCYKVGQNTVTFTDGPNALSINVNFGKPDYKFTSDKYTAFRSIPSLNEAQHYLVSGNCAKGYTSGTIGLAAAGGAQIDCSNWHVGYSNDFNDWAFPKSYSSDYTVTGLCLSSQVLVNFKNVPAGYRPFIDALFRVPDGQQVKVTYINEVQCVGSSSTDDYGEQAYGWGSYQNSEAGANGVIVVVTTSTIL

**>CmAls800**

MKNWHSLAAILTAISFSSFSLAAQVSNVFQSFDSLTWDNAAGYQYATPASPSWIATLSWTILGSNVHAGDTFTLNMPCVFKFTTTQQSVDLTVGDTVYATCQFAPGDLVVAYSQLKCTASDNVKDSTDATGSVHFPIAFNVGGSGGDVDLQDSKCFTPGTNDVTFTDGDKKLTTTADFEGGTNTNRGTSYDDIVFNDRVVPSLNKQQIYLLGGVCPNGYSSGTLGVSVSEGTLDCSVLHSAITNQLNAWYLPETAEAISASTSCTEQAFMVNYNNIPAGYRPFLDILVSLLPGESLQTYFTNRYTCAGDLFAQDNSKYTYWAPYDNDEAGANGNEVIVTTS

**>CmAls2265**

MAGLSTNVAWFATFMFVALTNAAQVSNIFQSFDNLVFAPAAAYNLEVPYAPSWNATLSWEIAGSKVKAGDTFTLNMPCVFKFTTSNPSVDLKVGNTVFATCKYAPGDLVVPYSQLQCTAADAVTDGSTASGKVTFPITFNVGGSGNAVDLQDSKCFTGGENTVTFNDGDKKLSTQVDFDSGSYWNDATKQDPAVQVAATRTVPSLNKQQFYLLGPQCQYGIVSGKLGIKSNAGWHDCSTMHAALTNQLNAWYFPESADKFSFTTTCNSRGLTVTYSNVPQDYRPFIDILVELQQGASNDVYYTNQYQCRYESEKRADLGYTWGTYNNSEAGSNGNEIKVITST

**>MgAls2302**

MIFWCFAALLTFALQAQAVALNGVFTSVNSIVATGSAVTYPWPQSTSWQATVSWMLDGGAVNAGDTFNLNMPHVFKFTSDTNTISLTANGVTYATCNLFSGEFIVDYSELQCTVASGLTTSNVAIGSINFPITFDPGFSSATPAIEGANFWVAGTNTVSWTTGSKTITGTVTFVAGALSATPAVNNYGAKLAPSTNQIQWFLMGTSCASTTQSGTLGISFASGGPTLQCGTLTAAITNMVNDWYFPKNAYSFSYTVSCSSSSVLITYSGVPAGYRPYINIYSPYLPAGQNVATYTQTYKCGTGATTPLTYTYNWIAYTNGNTGGNGQNFVNTIV

**>MgAls3259**

MFWLFAAFLLVVHVAADINGIFTNINSIVASSSAASTLYPASTYWTATVQWSINGANVNAGDTFALHMPHVFKFTSSSSSISLSAGGITYATCTFFSGEFMVNYSELQCKISPGLNFNDIAIGSVMFPITFDSGFSSSTNALEAANTWTTGTNTVTWTLGSYSISTTVDFEGGTSSSSPNLRVAAAKLAPSSSQVQFYSLGTSCPFETQSGTLGITFKNGGPPLQCSTLTASFSNQFNDWYFPQTASSLSYSVSCSSNSVMVTFAGVPAGSRPFFNIFASSLPVGRNSAVYTLKYRCGNGPSNDYSYDQGWSSYDNGNSGSSGGNFINTIV

**>MgAls3300**

MTPLFLFTLWAAVASAAQVSGVFTGFPSLVWQAGGNYYKPLPEFSSWVATLDWAIQGSKFKAGDTFTLHMPCVFQFTANTPTVQLKVNNDVYATCNFYSGEIITTYSELQCTMSDLVNDQTDVSGTLTVPMFFNTGLTANAVDLTNAKCFKAGDNTVTFTDGNNEISTNVNFNGGQMLGPLDPNNIIVGSRFVPSLNTLQHFLAAGKCDNGYISGTLGITVDNTNLDCSTLQAQITSQLNDWFFPESSQNFNFQTTCTGNTFTATYNNVPAGFRPFIDIMGSAPSGNNGFIVHYTNDYFCNGDPTVHSNGQGVNWGQPQNTDSGSDGNAAVV

**>MgAls673**

MRHCFSMMLLLFYVCFATVASAAKLSNVWTSLSITSPQGNRPQDIHSASLGWKIATADASSGDTFDIIMPGVFRTKFGEQQLRLVVDDTTYALCDAWDGSYITDYSSLSCTVTSAVNTDRDITATGVATFDFVFGAGGSSRSSDLYEASLYHAGTNTINWSGLSTTVDFVGGPFFNENDNDGLVYYSRSTPQLNEQLYVLSASCPAGITSGYTIATFENDYDCSEFSLKMTKDLNDFYLPEGYEDVPSSQVLCQSGRIVGSFSNVPAGYRVFLQGLYRYPLTSDTVYHNFGGNVQCSSGQTKTNNYGREIVVITGDSDSTGNSKEIIVTTTTW

**>ClAls3274**

MLITLFPVLLWFIALASAAQVSGIFTGINSITPGDKAWPKYPSWTASIAWKMDASKVKAGDTFKLEMPYVFKFTTSSSSISLTAGGKTFATCSLTSGENLLAYSEIDCTASSAITSVQSASGTFSVTFTFSSGATDEKLDLEAAAHWKAGANTVSFNDGTNTISTTVNMAAGATYFGSGHGSGAHGGRTLPSLNKLEMYFLDASSSRTRSGSLSLSVASNTPLDCSSSKAFLTTKVNAWEFPENSFDSVQGATFNFKCSSSRIDVTYTNLPAQYTPYIEAYSVIPVSNIFTVAYRSSDQYNPNRITVDNYQFAVYSNQQPSGDGSYIDIELTTIT

**>CauAls2582**

MKIISLLWCLALLYGNALAAPQTGVFTSIDSLTPFDVAWPMMPGWDATVSWHINSSMEMKDGDTFFLRIPFVIEFNTDESSIQMSDGTNTFANCVLTPGENLVPYSEVKCTATTQVEDVQSSSGTITFPIVFNAGFSAQESDLKAANHWRTGSNTLEWTDGSNTLTHPITFVGGTMSAFNGRPKRGILDQRSFVSTNTIRQFLMGPSCHSSDMSGELSIENLSEEAPFDCDSITTAMSNQINAWYFPQTADEAEATIVSCSAAGVNVAFSNLPAGFRPYINIDATKKIAVSEIDNIYHYNFTCNGAELSDSIFAAWDQFFSDDTEEDDTLTQVVVTT

**>CauAls4112**

MKLASLAAVSLAVAGAVAKLQSGVFNGIKSITPSDNRRPEQPSWHATVSWEIKPAMGVQEGDTFTLHMPYVYKFTSSSNTLQLTAGGQVVANCNLYSGENIVGYSEVQCTATAAAANAGTFTGDVTFPFTFNAGSTSDEVNLEAAGVWKSGQNTVTWSDGDKSFSTTVDFNPGASSIIQGSPENGVYGLRKMVSLNINQHYLMGPSCPYDGQYGRLEISNPSPGVGFDCSSLAGAITDQVNDWYFPKTAEKIGVNIDSCSSYQATVSFSNLPAGFRPYININAAIPNVASFRSSNTYSYNFVCGGRRQSGQSSIAWVMYNNGNTGSGGDFKPVVVTTVT

**>CauAls4498**

MVLASFLLGLAACSASVNASVKEGIFDKILSITPPEDSLPETPSWSATVEWSFDEKNGVKAGDTFVLHMPYVYKFTSGTRAVDLVADSVTFANCDLFSGDNVVAYSELKCTATSACEKVNSAKGTVEFPFTFNAGSSSDKANLEASTVWHAGSNVVKWTDGSKELHSEVYFDEGNPYLFSGSVEHGVYWLRKAVMRNTNQHLVLGPSCDCDGMSGYIEIQNPDYGVELDCSSVVGTITKQINDFYFPESAESCSVHVDECCATRVRVSFDNIPSGFRPYINVDSAIPHYDFNSRNQYSYGFSCGGIELCDSLWTNWYMYKDGETGGDGEFNSIVVTTVT

**>SsAls2386**

MLIVLAVAFLFSCVRAAVVSGVFTSFDSLVFQNGGNYPFDGPANPSWIATLKWQLDGTKVAPGDTFTLDMPCTFKFTQTPADAPVLLQAGGITYATCQTLGGEIIVPYSQLQCTVENAVTTSTLASGSVYFPVVFNIGGSATPVDLTDSKCFASGDNTVTFNDGDTKLSITANFETGYPASGVNPTNIIYRNRFLPQLGESQHLLVAGQCPRGYTSGTLGFSFSGGKLDCSSVHAAITNQLNDWYFPTDAETDFSFTYTCSASGYQITYKNIPAGYRPFIDGLTSATANLLTVSYTNKFVCVGSSINNDKSTKVTWSSYQNSDSGGDGHVIVLTT

**>SsAls2786**

MLRTIIFLFFYSIVRAAEITGVFTSFDSLVFQNGGTYPYEAPANPSWIATLSWAMDGNVVAPGDTFTLHMPCTFKLTTDNTIILGAEGTTYATCSHFSGEIIVPYSELYCTITDSLLPGMTVEGSTYFPVVFNTGGSALASDLEDSTCFTNGENTVSFYDGDTQLSTSVVFEAGPPSANINPDIVIFRARVLPQLNSSQHYAAIGWCPNGYTSGTLGFTFTGGTLDCDSVHASITNQLNDFHYPTNADPNFSYTFTCSPTSYQINFENIPAGYRPFIDGLTRPSGPALSVFYTNRYLCADETTFRQQNLNVNWGEYADGPTSGNGNVIVVTT

**>SsAls4579**

MCILVFVLITSVLGAQLTDVFQSLEIINNSGSNRAQDIRTAKLTWKIEAGDAVEGDEFSLEMPNVFRTKFPGDQLYLVADYSIYASCVAVDGSYLAQNSYLNCTTTSSVVESDFKATGTLSFDFVFNAGGSGSEIDTTAASILVPGENKINWSGLQTTVNIDAGPFFAPVSNDKELVYFSRSTPQMYEQIFMLAGECNGGIVSGSIGMTTNDSLDCTQFALKATNNLNSFLLPETAINVQNTITCKEQSITFKFNSVANNYRVFLQGLEKFPTNSDAIRHIFAYSIQCGDGTKITKSSGQDFVVIDGYEDSSGSVEYSTVYTTTTWTETYLTTVT

**>DhAls2178**

MKTSLITVLLSLVLLETTAAITVTNIWQSLKITNPSTSSRPQDIRTATVGWKIVTADVLHNDIFFFSMPYVFRTKFSGNEILLKADGTIYARCQVNDGSFNRDASYLKCSMTSSVDDKKDTTAVGEITFDFVFNAGGSSSDSDISSAKRFTSGDNQVSFNNIQSDVNFQSGPFFTDKKTDELLYYSRSTPQDLEQVFVLSGTCNEGITSGSMVFTTNNSVDCSQFKLKITNDLNDFYLPKSYKSIDVGSIRCSSDNKKLTATFNNIPSGYRVFLEGFEKYPDNSIYVKHLYAENIKCSDGSSKVDGITKIIRVVDGVSSSNGDTEVSSSI

**>LeAls734**

MLQQQLILLLHFVTLFTIVNAAQVSNIFTSFDSIVFNNARNHPYRAPHASFYRATLSWAISGADVKPGDTFVLNMPNVYKFVTNQAGVDLAVGGISYAKCTLVSGEWLVSYSQMQCVVTSNVKENTFVEGQIDLPISFNIGSTSEPADLQRAKVFKAGDNELVWSDGGRPLIQTVNFEAGNNYIPIGASNDKILIYGQYSAQLQEFDVRVVAGVCPNGYASGTLGVSNVNTPIDCNKIRTALAEQFNDWYIPTSVEPFDFKISCSSSSLVISYTNVPSKYRPMFDLRVHLESDETIIGSWRNSYQCLGGGTYENSYRVGWTPGTTENIDINNGYVVEVIT

**>LeAls2536**

MLHYLVQALAVLSIVSAAQVSNVFQSFNSLVWQNGASYALSTPAAPNWIATLSWQILGSNVKAGDSFTLHMPCVFKFTTTQTGVSLAVGNTVYATCNFEPGDLVVAFSELQCVATSNVQASTNAYGTVSFPFTFNVGGSANAVDLQSSTCFNSGTNTVTFTDGGNPLSISVNFQGGSNTNAATGVSTNNIVYSNRIVPTLNRQQVYLLAGSCPNGYTSGTLGVSVTSGLIDCLSVHAAISNAFNAWYYPESASTFLYTNTCSTSGFSISYSNVPAGYRPFLDMLVAVATGASLTTSFTNKYKCVGSSTTVDNSKSVTWGAYNNNQAGANGQEVVVTTVTWTGLTTLVT

**>LeAls2716**

MITLILRFLTLLALIQAAQVSNVFKSFDKIQYVQAGLYDYKVPAFAYWKSTLSWNIDGQNVKPGDTFTLHMPCTYRFYSSQSTIQLSFAGKAYADCTLFSGEDLVTFSELQCTATSLVNEDTNVAGLIDLPLIFNQGGGGSKNDLECSTFYKGGTNTITFSDGGEPLSTTVNFELRNYYVTQSPENYLLMNSRYSSNLQRLYIRLTGADCPNGISSGVYTIDNLYTTIDCSHVATYIATSFNGWKNPTEAEPFDMRVTCTRNSVSISYNNIPAGYRPLFEVTFNLGLGQQARANVYNDFQCVGQSAKSNSEPFGWQPASVGNVNVNEGYEIDYITLT

**>LeAls2721**

MLFQIIQVLVVLSIAKAAQVSNVFQSFDSLTWQNGASYPYRTPAAPNWIAQLSWKILGSNVQAGDSFTLHMPCVFKFTTTSTGVSLAVGDTVYATCNFAPGDLVVAYSELQCVATSNVKASTNAYGSVSFPLTFNTGGSANAVDLQDSKCFTEGTNTVTFTDGGNPLSIQTNFQGGSNTNAQLGIGTDSIIYSNRVVPSLNKQQLYLVAGNCPSGYTSGTLGVSVTSGLIDCLTIHAAISNSFNAWFLPTSASSFQYTTSCNSNGYSIRYSNVPAGYRPYIDLLALVATGSRLRTVFTNQYRCSNSLFTTDNSRTVTWSPYNNDQVGSNGEEVVVTT

**>LeAls5708**

MLFQIIQALVLLSIVKAAKVSNVFQSFDSLTWQKAGNYVYKTPAAPSWIAQLSWKILGSNVQAGDSFTLHMPCVFKFTTNSAGVSLAVGDTVYATCNFAPGDLVVAYSELQCVATSNVKASTNAYGTVSFPLTFNTGSSANKVDLEASKCFTAGNNRVTFTDGGNPLSIQVDFEGGSGINYQMGIGTDSIVVSNRVVPTLNKQQLYLLGGNCSNGYTSGTLGISVTSGLIDCLTVHAAITNFVNAWYYPTLASPFEYTSDCNTERFTINYSNIPAGYRPYIDLLVLVATGSPLGTVFTNQYRCSNRPNTIDSSMALTWSPYNNNQIGSNGEEVVVTT

**>YtAls93631A**

MIRLFWVLLWFCASPIAATQVTGIFTSFNDLTFQSSGYDQIPLSPSWYATLGWTINGANVAAGDTFTLTLDCVLKFTDSASTFNLAVGSTTYATCTYSNGELIVPYSTINCVVSSSITTSTSASGTVSFPFTFNPGGGGSSVDLTDSTCFSAGTNTASFHDGSNTLTHTFTVISENTSSNAKSYQLVRSMPSMKRTQIYHLGATCNGGIVSGSFGMTVSGFDCNNMHVGITNNLNDFLMVKPTYSSFSMTGTCSTSGYVVTFSNIPAGYRMFMDTLVPFPSNGYLGVTLTNTVVCGDGTTINANQFRDWNTYSSGSSGSSGNAIIVTT

**>SpAls49824**

MKMILSVILLPVLITFASAQQISGVFTSFNSLEWQNGGGYPFAGPYFPSWKAKLGWKIDGSSMHGGDTFTLTMPCVFKFTTNEESVELSSGLTNYATCIFAPGSILSSTSELKCTILDTIQDSTLTTGTLTLPVTFNVGGSALGSDVYCANFFEGGSNTVSFSDGDNILSTEATFEPDTQTDPNGTMYGARIIPNKNEAQIYVLSGECAQGLSSGTIGMTITDGSSIIDCSSVHAAMSNSLNDWYFPESADLLSTSTSYCNDSSFMVTYSNIPNGNRVFIDGLMSIVNGESPTVKYTNQYICDQSEGLIDGTTEVKWQPSYVNINDFDVTV

**>SpAls50348**

MIPVFLSIIFLVSFVNGCELFTSLDEVVFITPSMHDPAIPYKPRWDATLSWAINGKEIKPSKRFTMKMPCVYKFCTDKSYIPLIANHVEYGQCYFKSGEIFDKYSKLECVISNNVIKTTNVKGQIRVPLVFNAGGSALCTDRECAKLFKGGSNVIEFSGGKKFRYTVNFTPGLPYNTDKGVFHIRGVPSKNKVQPYLVAGNCPQGYSAGYFGFEIRDSMTKIDCCSYHAKKSKYNEFNSWMFAKTEHSISKKASCGNNRLEVTYKDICKDYRPYLDALLVPTVGHPVKIYYYNRWMCKGDPTEHRNDGYYEWTPESCDPTNVVTTTLPT

**>SpAls50349**

MLSVLLSVISLVSLATSCELFTSLDQVYYVKQDPNDLPKPYKPHWFAALSWAIKGTEIFPNNHFYLTLPCVYKFNTDKDYVPLIGTDNVEYGQCFYNPGELSVSYSKLDCVISNNVVKTTNVKGQIHIPLTFNAGGSHHTLDKDCAEKYDAGSNVIAFSGGKAFKYTVNFAPGLPYNPDNGVFHIRYHPKANRLQPYLVAGNCPKGYSKGYMGFEMKTTTARLDCGHWEAKRAESKKFNSWMFAESDQHLSKSVTCSNNILSVYYKDVDKGDLPYLDAIIVPTDGFPVKMVYYNRYTCKGESIEHIHDGYYEWDKDSEYPTAVTT

**>SpAls55077**

MISLIFFFSILLTSVLTKEISGVFTSFESLVWEPGAYFAFSSPATPSWMVTMGWEIDGSTMSAGDTFTLTMPCVFKFTSDAKHVDLNATHSITGATTTFATCYFNPGELLVTYSELLCVLSDEVDSETKAYGAITIPVTFNNGRGASDTDLECAAMFHSGTTTVSFRDGDNILSIDVEFEKGCEYLNPLLRHNRISLLFDNSQLVIFAAECPDGYDSGTLGIQLVYEEHTINCNYARAAITNELNDWCCAKSIAPFTFTTICNQSTFIVEYENIPAGYRPFLDILVRAPPPEKMNMTYIETHYCSGSSELIDNTYLEYWGSYINKNPWSDGREIVLTT

**>SpAls59511.5**

MISVLLLLTIWLTTVFSREISGVFTSFDSLVWDPGELVPGDGREKIPPPARPTWLATLNWEIDGSTMKAGDTFTLTMPCVFNFVISEPSLDLLITDPTTGLNTTYATCILNPGEDIVPYSELQCTLSDKVEGSTQVTGSLEVPMVFNVGMSKADTDLICASMFHTGINTISFTDGHNVLSADADFKDPIQDPSYGRVRISTIVDKLQIYMLWPAEGYWGCGWIELTLTLPYSDKAKFICDTADAGMTNLLNDWQYPIDHDNISYNKNCSEWEYTAHFPFREGYDPFISIYAKMPNGFPTFVGVETLYQCGDFMKTWSGFGFGEYNNTNPLPAENVEVVTSTYTGSVTTVTTVSYETSVDHTITIIV

**>SpAls61022.5**

MILSVVLLPLLLTFVSSQEITGVFTSFNSLVWNNGGNYAYASPASPSWMATFNWNIDGSTMSPGDTFTLTMPCVYKFTTTQSSIDLVVGSTSYATCVFSPGDVLLTTSAMECTLLNTVQDSTSAHGTVTVPLTFNIGGSALDTDVYCASVFKNGLNTISFSDGANILSTQATFEEGTGTDPNEVVYRARIMPTVNKWQHFLLGGNCPSGYTSGTLGMKVNDNTGTIDCSAVHAAISNKLNDWYYPESISTSFSFTTTCSSTGYTITYNNVPAGYRVFIDTFISPVPGVMTYLSYTNQYKCSDNPSQQIDNSANVQWSLYSNNVAGANGQEVHLVTETWTGSTTKVITKPYNTNDPTITIVV

**>SpAls64434**

MYLLLILLQLLTFAATQEISGVFTSFNSLTWLNSNPNTFQTPASPAWIAELSWKIDGSVVSAGDTFSLTMPCVFRFYTDQDSVNLRVGSTNYATCDFITGGISSTDSELNCVLSDEVEESILAVGKLTFPIVFNIGRSTEDVDLYCSTFYQGGLNTISFMDGDNTLSIDVEFQDGTPSGDPNGPMYGARMLPSSNRWQNYVLGGLCPGGLSTATIGITINDDVSTIDCETANIRLTNAVNDWFYPESFKTFTTASKLCRSNSFMITYWGVPDGYRVFIEAVINTDGNPPRVQYTNKYLCPTTIDNGVWVNWLPYDSVEADGPGKEIITVTETWTGSDTETITKSFDEDEDLTITIAV

**>SpAls64435**

MYLLLVLLQLFSFVATQEVTGVFTSFDSLTWLDSGNYHIKYPAAPAWIAELGWKIDGSLVSAGDTFTLTMPCVFKFTTTQSSVNLHVGDINYATCDFAPGGLFSANSELHCILLDAVQDSTLVVGKLTLPIVFNTGRSSADVDMFCSTFYKSGLNTVSFTDGDKTLSIVVEFKGGSHSGDINRAIYSARILPAGDQGQHYLLGSYCPGVLKMATIGMTINDGASTFDCSTAHVYLTNSVNDWYYPQSFESIDPVFITCTPKEFLISYKEVPDGYRVFIEAILNIVDGVNPIVYYNDMYMYTTPIDKSIEVTWYPYTHVEADGPGKEIITVT

**>SpAls68952.5**

MISPLLLFIFVFTSVLARQLSGVFTSFDSLTWDNAGNYQVSAPSYPTWNAKVSWEIDGSTMKTGDTFILSMPCVFKFITPETHIYLNTTNPDKDSTTTFATCVLSPGDVIVPFSELQCTLSDEVKDSTLAYGSITIPMSFNVGWAGSDTAMKCANMFKDGQNTVSFTDGNRILSTEVNFQGGYGPDDPNNIVRQGRISPLLDVSFQFFMGKNCPNGYESGTLGIELQTPNGKFDCSMQESGMSNKLNPWYYPTTIEPVAISSTCDEHLFKFDYGSVPAGYRPFISIPARLPLGASLVVHYIDYYQCKGEEKQTKRKLSANWFNYENSNPLADGREIIVKTQTYTGSATRLTTLPYNPDAGRTMTIVV

**>SpAls131476**

MILLLVFILLGSLATSFELFTSLDEVVYVKHAINDQAKPYQPHWDATLSWAIKGTEVHPSDHFYLKMPCVFKFITDQTYVPLKAGSIEYGQCHFKPAEVFSTYSELDCVISSTVSASTNAKGTIRFPFTFNVGGSALDADLKCAKTFTSGSNVIAFSEGKKFTYTVEFDAGSSYNPDGTFFARTVPSVKKFQTYLVAGDCSKGYLSGTLGLQITNSNAKIDCSSFHANMTNQFNSWMMAGTADSITRTISCVDNKLEVNYKNIPAGYKPYLDAFLIPTDGHSIKMVYFNRYKCIGDDNQTNHDANYEWTAYDNSDPNSEGEDGFPAIGTELSS

**>SpAls134426**

MILSLVLLQLLATFVSTQAISGVFTEFNSLTWENGGGYPYASPSSPSWMAKLGWKIDGSTMNSGDTFTLDMPCVFKFPTTQTSVDLKVGSTSYATCTFSIADILLSSSQLNCVLLSAVQDSTSATGTLTFPVAFNVGGSASSNDVSCASRFQDGVNTVTFKDGSNTLSTQATFQKGVDSDPNKIIYHARTLPMLNKMQHSLAAGNCASGYSTGTLGIKIINNSGQVDCANIHAAISNSLNDWYLPKSVSSSFSFTTTCSSSGYSISYQNVPAGYRVFIDALVSIVVGGDNTVQYLNKYVCSGSTVNNDNSLNVEWKNYQNNVAGSNGQEVVVITETWTGSTTHLTTKTHNSDDPTITVIV

**>SpAls134590**

MISLIFFFSFLLTSVLTKEISGVFTSFKSLVWESGSKYTYVSPSHPSWMVTMGWDIDGSTMSAGDTFTLTMPCVFKFTSEYKHVDLIATHPITGATTTFATCHFNPGDILVTYSELLCVLSDEVDSETKAYGAITIPVTFNAGRGASDTDLACAAMFHSGTTTVSFRDGDNILSIDVEFEKGCDHLKPLLRHSRISLLFDNSQPVIFAAECPDGYDSGTLGIQLVYEEHTINCNYARAAITNELNDWCFAKSIAPFTFTTSCNQSTFIVEYENIPAGYRPFLEILVRAPPPAKMNMTYIETHYCSGSPELVNNTYMEYWGEYKNVSPWSDGREIVLTTRTYTGKYTTVTTLSF

**>SpAls134874**

MFKFLIFNLLALTLVASEQISGIFTSFDSLVWQKAASYPFAGPAYPSWISTLSWKIDGSTMSPGDTFTLNLPCVFKFTTSQTTVNLNVGSVNYATCTFNPGDIVVAFSKLECVLLDTVTPSLDAHGTVNFPVAFNVGGSALSTDLQDSTCFHNGQNTVSYYDGDNKLSTQATFAGGSTDDPSKIVYVNRVVPSLNKQQHYLLGGNCPSGYRSGSIGMKIQNSGSQIDCSSVQMAITNSLNDWYFPENADENFSYTYSCSSTSFVVNYQNIPAGYRPFIDALVQVNNGVSISMNYVNTYTCTGSSRSTNNGKTISWGAYENNVAGGNGEAVEVVT

**>SpAls135549**

MFRFLIFTILTFAIVSAEQISGIFTSFDSLVWQQSGTYPYTNGPGFASWIATLSWKIDGSTVSPGDTFTLNLPCVFKFTSTTPTVNLTVGSVNYATCTFNPGDVVVSFSQLQCVLLDTVTPSLDANGSINFPIVFNVGGSALNTDLQDSTCFHSGQNTVSFYDGNNQLSTQATFNNGSFTDPNSIVFNNRAVPSLNKQQHYLLAGNCPAGYRSGSIGMNIQNSGSQFDCSSIQMAITNALNDWYFPETAGNFSYTYSCSSTSFVVNYKNIPAGYRPFIDALVQVNNGVSVVMNYVNTYTCTGSRSSINNGGTTTWGAYENNDAGGNGEAVEVVT

**>SpAls136382**

MLSFIIFIILTIATVSSEQISGVFTSFDSLVWEKAADYPFAGPSYPSWIVKLSWRINGNDMNAGDTFTLDLPCVFKFTTNQASVNLNVGKTNYATCAFNPGDIVVAFSKLECVLLDTVTSSTNAHGSINFPVAFNVGGSALSTDLESSTCFVDGRNTVSFYDGDNQLSTQVSFSGGATDDPNKIVYRNRVVPSLNKQQHYLLAGNCRARYKSGSIGMKIQNQGSKINCDSIHMAITNSLNDWYLPKNANNDFKFTYTCTSTSFMVEYKNIPAGYRPFIDSLFDVANGVSVSVNYVNNYICADSTKTTDNSKSINWSAYENNVVGGNGEEVEVVTSTYTGSTTQISTMPFQTSKDKTMTIVV

**>SpAls137089**

MFSKILAFIFLATYVATTQVTGVFTSLDKVEFKPYSNKDPARPYQVHWEATMSWEIQGSKIQPLDTFTLDLPCVFKFITDEPYIALTVGSTEYAHCYFQAGEVFLTFSQLNCVALSSVKDSTDAHGTVRFPFTFNAGGSSLDVDLQCSKKFTSGSNIISFYEGDKEFRYSVNFVTQLLTHTDDITFHARSIPTLNRLQTYVVAGKCTKGYTSGTLGFEMLDSSSKIDCNSYHASMSSQFNSWMMAGTAENMEMTVSCTDSVLIVEYQNIPVGYRPYLDANLAVQEGAATPMIYHNSYTCVGSRYETDNDKDHEWSKYSNSNADSEGMEVVFAT

**>SpAls138016**

MLYFILLFLVSSIFAAEVSPVFTDFISCEWENGGDFPFALPINPKWMVKVGWTIDQSTMQVGDTFTLHMPCVYKFPTSDKTFKLSHNNIDYASCQLKPGDVVLGYSEVSCVINEVPSAGSVSGEATFPVTFNAGLSGAATDLQCSSRFHEGTNTIIFNDGNTEISTTVNFQKDTDNDFDKIAYVHKYIPNLNRFQDYLLAGNCPNGYKSGKLGIQLALTSEVIDCSNAKAHISNMFNDWMYPTAAQNLIASIECGPDSFTVEYGEIPPGYRPFITAFLLDPQFSGTYVNYINTYTCKGEDKEHENDEVVLWKPSNDGMTGATAQRVIVVT

**>SpAls140483**

MLRFFLFTIFLVAASAEQVSGIFTSFDSLVWEKAANYAFASPAYPSWIATLSWKFKGTEVSSGDTFSLTLPCVFKFTTSQTTVSLNVGSTNYATCTFNPGDIVVSFSQLECVFLDSVTSSTDAHGSINFPVAFNVGGSALSTDLQDSTCFSDGENSVSFYDGDNKLTTQASFSGGSTDDPNKITYINRVVPSLNKQQQYLLGGNCPSGYKSGSIGMQIRNSGSSIDCDSIHMAITNSLNDWYLPQNADVDFSFTYTCSSSSFIVNYQNIPAGYRPFIDALFEVSNGVSVSVNYVNNYICTSSSTTTNNGKTITWSAYENNVAGGNGEAVEVVT

**>SpAls140900**

MLRFFIFSFLVLATATADQVSGIFTSFDSLVWQKAANYPFAGPAYPSWISTLSWAIKGTDVSPGDTFTLTLPCVFKFTTSQTTVSLSVGSTNYATCTFNPGDVVVAFSQLQCVVSDSVTPSTDAHGTINFPVAFNVGGSALSTDLQDSTCFADGTNTVSYYDGDNKLSTSVQFTGGYNGSPSQIIYTNRVVPSLNKQQHMVIAGDCPAGYRSGSLGIQIANSGPKIDCSSIHIAITNALNAWYLPENADTDFSYTYSCSTTSFTINYQNIPAGYRPFIDTLVSVATGSSITTTYVNNYVCANSILTTNNGKTVSWGNYDNNVVGGNGQAVEVVT

**>SpAls141433**

MLNFFIYFVLVFSFTIAKQISGVFTSFDSLVWKKAANYPFPAPQFPSWIATLSWKIKGSNMSAGDTFTLTMPCVFKFTTSQRSINLSLGTTNYATCTFNPGDIVVAFSKLECVLSNSVNPRTDSFGTINFPITFNTGGSALDTDIQDSTCFVNGKNVVTFSDGNNKISTSVLFSGGSNEDPEQVVFKSRVIPTLNKQQHFLLAGNCPSGYTSGTLGMQIVSLGATIDCRSIHMAITNSLNDWFMPKNTKLKFSHTFVCDPVTFVLTYQNIPKGYRPFIDALVSVILGSKVTVIYL

**>SpAls146555**

MFLSLIFLQILLTFAAAKEISGVFTSFNSLKWRKGANYRYASPAFPSWIAELGWKIDGSKMSAGDTFTLTMPCVFKFTTTQVSVNLKVGNTIYATCRFAPGDILLPTSELKCSLSDTVKQSTSAHGTMSFPLTFNVGGSALDTDVKCGETFTSGVNTVSFSDGDNVVSTTVNFEGGVDTNPDRIAYNARVVPSVNKLQHQVLGGKCGAGYSSGVLGITMLYGTGSIDCSSIHAAISNSLNDWYFPKSIETDFSYTTKCSATSFIINYKNIPAGYRVFIDTLVSSAVGVKHKIRYTNRYKCANSPYIIDNSETVQWQPYCNDVAGANGKEVELVTETWTGSTTLVYTKPFKTHDPTITIVV

**>SpAls152224**

MLKFAVFTILALATATAEQVSGIFTSFDSLEWEKAANYGYASPAYPSWIATLSWAIKGSKVSAGDTFTLTLPCVFKFTTTQTSVNLNVGSTNYATCTFNPGDIVVAFSKLDCVMLDTVTSSLDAHGSINFPVAFNVGGSALSTDLEDSTCFADGENTVSFFDGDNKLSTQAIFSGGSTDDPEKIVYGNRVVPSLNKQQHFLLGGNCPKGYSSGSIGLEIKNSGPKFDCDSIHMKITNSLNAWFLPENADDDFDFTYTCTSTSFVVDYKNIPAGYRPFIDTLVDIANGQSITLNYINNYICDGSKTTTNNGKIIAWAAYENNVAGGNGEEVKVVTSTYTGSTTEVSTMPFETSKDKTITIVV

**>SpAls153035**

MISYLILVTFSLSITLSKQITGVFTSFDSLEWEGKGEPMPAYPTWWASLSWEIDGSIMNSGDTFTLHMPCVFKFATSEDQLDLVTVNPDTGNPITYAACNLISGDVVVSFSELQCILSEEVQASTHVFGSLNVPIIFNIGFAGSNVDLVCASMFQKGTNMISFSDGDTIISTEAEFGQGRTQMFRESSVLNKAELILVHDNFDLCGEGLSLLGLVMEFETPGGRFDCNSMGVGITNELNAWGAPTTFEKLLEPIPICDNTLFAYDFRRGVPPGFPFIYISVKLPNRQPYFITLSTIYNCDGGGDAYRTTSSISGTYANDDPGPDVDAVDLVTQTYTGSSTQVSTVTFDPNLDRTMTIIV

**>SpAls153035.5**

MEVKQATMISSLVFLAFSLVTVLSQTTHLFKSLESLEWEGESLFWKNTPFTPTWWASVSWKIDGSIMSPGDIFTLEIPCVYKFDTGVGKLCLTSTNSDTGESIIYGTCDLLLESIYFGRSLVHCTLSDEIEQDTIVEDTLRVPIIFNAGFTEDPKDISCAGSIREGINDFTIYEDNELLVTKVEFRSTDDRGHNQQFRVSDSSDKVDIILSDNHCAHGYSMISLGMYVTADSGGLDCSTAREGITDLLNAWGYPVYYEGTSGERTCNGEQYIFEYNSLSYGKYPFILISANLPPSDSTEVRFVIDYI

**>SpAls155003**

MLFLLLLLALQTALGATKYVNAFTSFDDLKFMTPKRPSQPFQVYWEATLSWSLKSSEIQPYDTFGLILPCVFKFATNENTLTLKVNEVELAFCSLISGELKETFSKLDCIALPGVTLGNSKGGIKLPFTFNVGGSALSTDLETSKCFTAGKNIISFYDGDKELSYPAFFETMFESGGKHDADEVVYGARSLENMKWLQNYLLAGNCPQGYTSGKLGFTILQGAKIDCNTIHAYISNKFNDWFFPTTFEEMKMTINCSENEVLIEYQNISPGYRPYLDGLISTRLGHDTKMKYVNQYTCNNETVKSNDVIVNW

**>SpAls156463**

MILSLLLLIFFSTIVSSKDNTGFFTSFDSLVWEKGGNYPIISPATPNWIGTFSWTINGTTMEPGDTFTLHMPCVFKFTTHETMIEVGTDDKIFAICTFHPGDIVVAFSELKCTLLDTVTPLTEAYGTLRFPFTFNPGTSAINTDLKCATKFFDGLNTIIFTEGDKELSTQATFEGGYTYTDPDDLVYSNRIIPTLNKMQPYLLGGNCPDGYESGKIGMEILRGTGLIDCNSIHARISNSFNPWYHPRSASNSFDYTTECTSTSFVVEYQNIPAGYRPFIDMLVKVQKNVQLSLKYINTFTCANSSSEVNKGTELTWTPYRNDNADAEGKEVVAITS

**>CAGL0G04125g**

MLFIGIIQILLLIQYVCTTEIRNIRFSNLKLDPLTDQAHPHQGWKAAFDFQIPDSFKVYKDDYFELELPRVYIIKFAKDSTELLIPLKDRNSEEIFHCSVPQQAAYKYKSTILRCVTLTDLAPHPEISGHIEFSLSFSNGDSTYQYELENANYFESGLNNIEFTQELNAEVYFDAAKFNDHFYTTIRSTTYKEVEIYFLSMRCPNGYLLGGSQKINFDSKDANCQLNCNTPQIFVSKHFNDWWFPKSYQEIVDADILCFGNNLWITIGEQEKGHLLWVNAMQDVADGQNTLYHDVFIEYTCSDTIAKTTYQTEHTAHLEYRIY

**>ScSag1**

MFTFLKIILWLFSLALASAININDITFSNLEITPLTANKQPDQGWTATFDFSIADASSIREGDEFTLSMPHVYRIKLLNSSQTATISLADGTEAFKCYVSQQAAYLYENTTFTCTAQNDLSSYNTIDGSITFSLNFSDGGSSYEYELENAKFFKSGPMLVKLGNQMSDVVNFDPAAFTENVFHSGRSTGYGSFESYHLGMYCPNGYFLGGTEKIDYDSSNNNVDLDCSSVQVYSSNDFNDWWFPQSYNDTNADVTCFGSNLWITLDEKLYDGEMLWVNALQSLPANVNTIDHALEFQYTCLDTIANTTYATQFSTTREFIVYQ
