# Supplementary material for: Using Genomics to Shape the Definition of the Agglutinin-Like Sequence (ALS) Family in the Saccharomycetales
Source: Front Cell Infect Microbiol. 2021 Dec 14;11:794529. doi: 10.3389/fcimb.2021.794529 (PMC8712946; doi:10.3389/fcimb.2021.794529)
Supplement: Supplementary file 6 [file Presentation_1.pptx]

## Slide 1
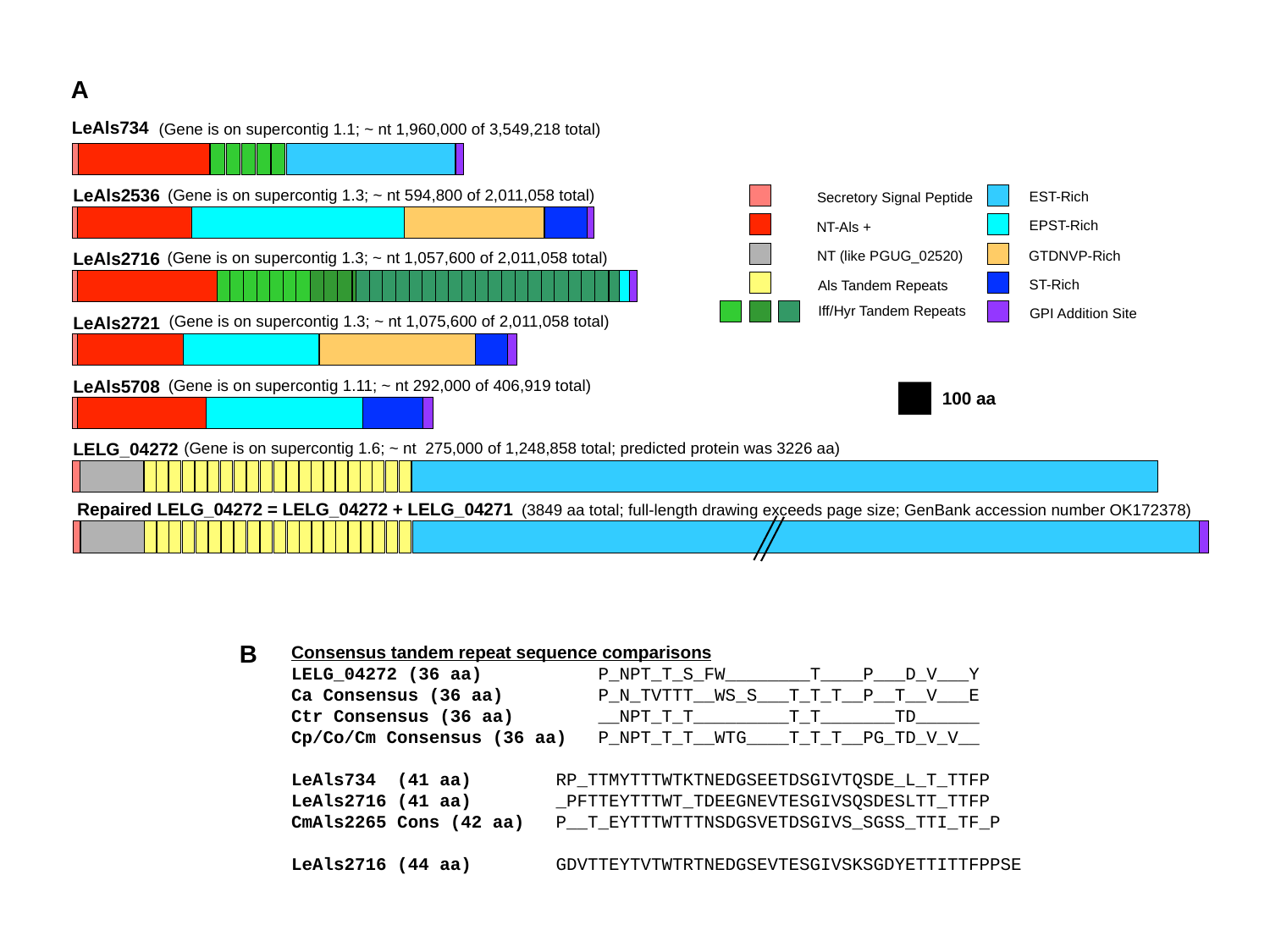

A
LeAls734
(Gene is on supercontig 1.1; ~ nt 1,960,000 of 3,549,218 total)
LeAls2536
(Gene is on supercontig 1.3; ~ nt 594,800 of 2,011,058 total)
EST-Rich
Secretory Signal Peptide
EPST-Rich
NT-Als +
GTDNVP-Rich
NT (like PGUG_02520)
ST-Rich
Als Tandem Repeats
Iff/Hyr Tandem Repeats
GPI Addition Site
LeAls2716
(Gene is on supercontig 1.3; ~ nt 1,057,600 of 2,011,058 total)
(Gene is on supercontig 1.3; ~ nt 1,075,600 of 2,011,058 total)
LeAls2721
(Gene is on supercontig 1.11; ~ nt 292,000 of 406,919 total)
LeAls5708
100 aa
LELG_04272
(Gene is on supercontig 1.6; ~ nt 275,000 of 1,248,858 total; predicted protein was 3226 aa)
Repaired LELG_04272 = LELG_04272 + LELG_04271
(3849 aa total; full-length drawing exceeds page size; GenBank accession number OK172378)
B
Consensus tandem repeat sequence comparisons
LELG_04272 (36 aa) P_NPT_T_S_FW________T____P___D_V___Y
Ca Consensus (36 aa) P_N_TVTTT__WS_S___T_T_T__P__T__V___E
Ctr Consensus (36 aa) __NPT_T_T_________T_T_______TD______
Cp/Co/Cm Consensus (36 aa) P_NPT_T_T__WTG____T_T_T__PG_TD_V_V__
LeAls734 (41 aa) RP_TTMYTTTWTKTNEDGSEETDSGIVTQSDE_L_T_TTFP
LeAls2716 (41 aa) _PFTTEYTTTWT_TDEEGNEVTESGIVSQSDESLTT_TTFP
CmAls2265 Cons (42 aa) P__T_EYTTTWTTTNSDGSVETDSGIVS_SGSS_TTI_TF_P
LeAls2716 (44 aa) GDVTTEYTVTWTRTNEDGSEVTESGIVSKSGDYETTITTFPPSE

## Slide 2
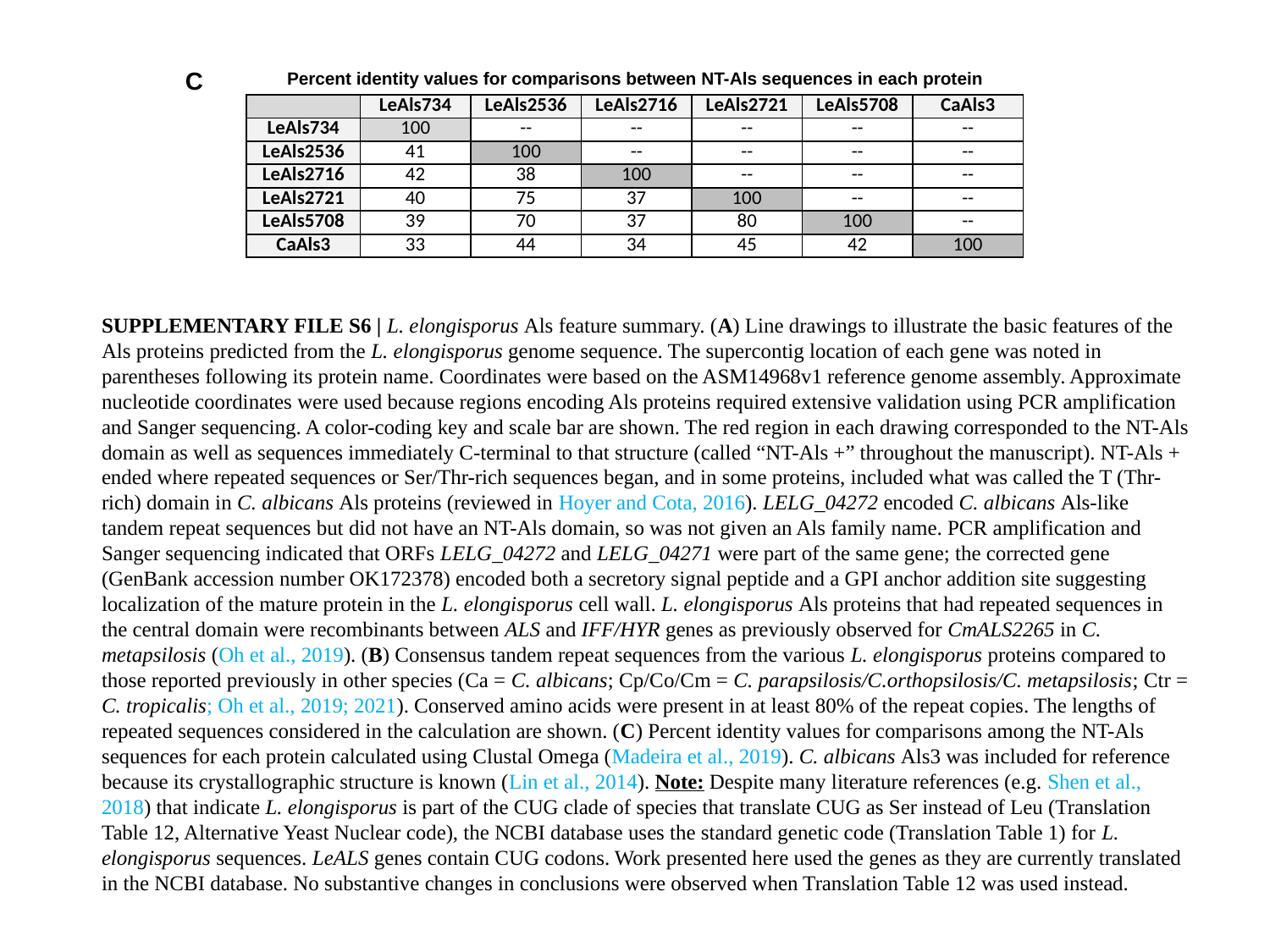

C
Percent identity values for comparisons between NT-Als sequences in each protein
| | LeAls734 | LeAls2536 | LeAls2716 | LeAls2721 | LeAls5708 | CaAls3 |
| --- | --- | --- | --- | --- | --- | --- |
| LeAls734 | 100 | -- | -- | -- | -- | -- |
| LeAls2536 | 41 | 100 | -- | -- | -- | -- |
| LeAls2716 | 42 | 38 | 100 | -- | -- | -- |
| LeAls2721 | 40 | 75 | 37 | 100 | -- | -- |
| LeAls5708 | 39 | 70 | 37 | 80 | 100 | -- |
| CaAls3 | 33 | 44 | 34 | 45 | 42 | 100 |
SUPPLEMENTARY FILE S6 | L. elongisporus Als feature summary. (A) Line drawings to illustrate the basic features of the Als proteins predicted from the L. elongisporus genome sequence. The supercontig location of each gene was noted in parentheses following its protein name. Coordinates were based on the ASM14968v1 reference genome assembly. Approximate nucleotide coordinates were used because regions encoding Als proteins required extensive validation using PCR amplification and Sanger sequencing. A color-coding key and scale bar are shown. The red region in each drawing corresponded to the NT-Als domain as well as sequences immediately C-terminal to that structure (called “NT-Als +” throughout the manuscript). NT-Als + ended where repeated sequences or Ser/Thr-rich sequences began, and in some proteins, included what was called the T (Thr-rich) domain in C. albicans Als proteins (reviewed in Hoyer and Cota, 2016). LELG_04272 encoded C. albicans Als-like tandem repeat sequences but did not have an NT-Als domain, so was not given an Als family name. PCR amplification and Sanger sequencing indicated that ORFs LELG_04272 and LELG_04271 were part of the same gene; the corrected gene (GenBank accession number OK172378) encoded both a secretory signal peptide and a GPI anchor addition site suggesting localization of the mature protein in the L. elongisporus cell wall. L. elongisporus Als proteins that had repeated sequences in the central domain were recombinants between ALS and IFF/HYR genes as previously observed for CmALS2265 in C. metapsilosis (Oh et al., 2019). (B) Consensus tandem repeat sequences from the various L. elongisporus proteins compared to those reported previously in other species (Ca = C. albicans; Cp/Co/Cm = C. parapsilosis/C.orthopsilosis/C. metapsilosis; Ctr = C. tropicalis; Oh et al., 2019; 2021). Conserved amino acids were present in at least 80% of the repeat copies. The lengths of repeated sequences considered in the calculation are shown. (C) Percent identity values for comparisons among the NT-Als sequences for each protein calculated using Clustal Omega (Madeira et al., 2019). C. albicans Als3 was included for reference because its crystallographic structure is known (Lin et al., 2014). Note: Despite many literature references (e.g. Shen et al., 2018) that indicate L. elongisporus is part of the CUG clade of species that translate CUG as Ser instead of Leu (Translation Table 12, Alternative Yeast Nuclear code), the NCBI database uses the standard genetic code (Translation Table 1) for L. elongisporus sequences. LeALS genes contain CUG codons. Work presented here used the genes as they are currently translated in the NCBI database. No substantive changes in conclusions were observed when Translation Table 12 was used instead.
