# Supplementary material for: Using Genomics to Shape the Definition of the Agglutinin-Like Sequence (ALS) Family in the Saccharomycetales
Source: Front Cell Infect Microbiol. 2021 Dec 14;11:794529. doi: 10.3389/fcimb.2021.794529 (PMC8712946; doi:10.3389/fcimb.2021.794529)
Supplement: Supplementary file 7 [file Presentation_2.pptx]

## Slide 1
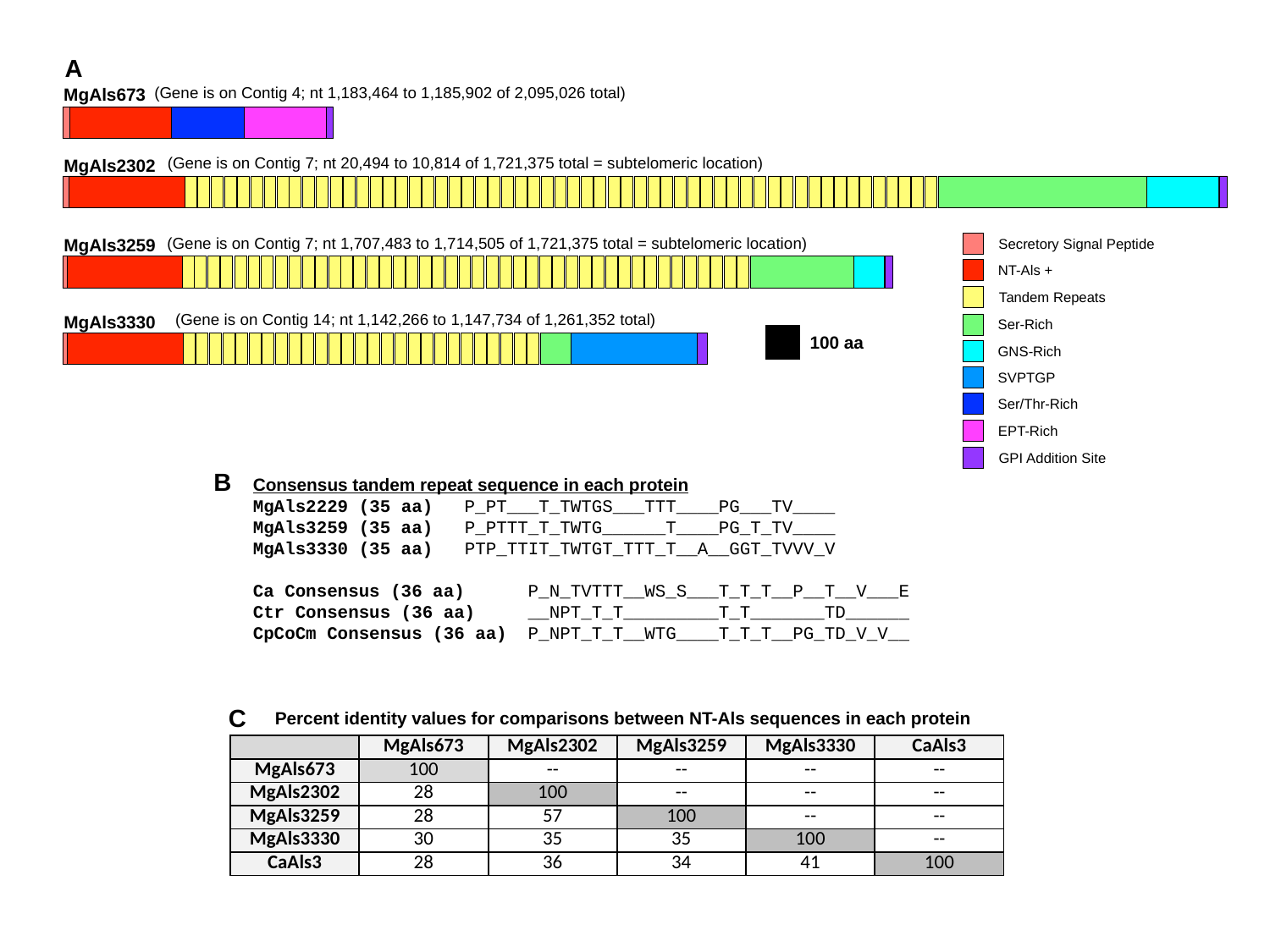

A
(Gene is on Contig 4; nt 1,183,464 to 1,185,902 of 2,095,026 total)
MgAls673
(Gene is on Contig 7; nt 20,494 to 10,814 of 1,721,375 total = subtelomeric location)
MgAls2302
MgAls3259
(Gene is on Contig 7; nt 1,707,483 to 1,714,505 of 1,721,375 total = subtelomeric location)
Secretory Signal Peptide
NT-Als +
Tandem Repeats
Ser-Rich
GNS-Rich
SVPTGP
Ser/Thr-Rich
EPT-Rich
GPI Addition Site
(Gene is on Contig 14; nt 1,142,266 to 1,147,734 of 1,261,352 total)
MgAls3330
100 aa
B
Consensus tandem repeat sequence in each protein
MgAls2229 (35 aa) P_PT___T_TWTGS___TTT____PG___TV____
MgAls3259 (35 aa) P_PTTT_T_TWTG______T____PG_T_TV____
MgAls3330 (35 aa) PTP_TTIT_TWTGT_TTT_T__A__GGT_TVVV_V
Ca Consensus (36 aa) P_N_TVTTT__WS_S___T_T_T__P__T__V___E
Ctr Consensus (36 aa) __NPT_T_T_________T_T_______TD______
CpCoCm Consensus (36 aa) P_NPT_T_T__WTG____T_T_T__PG_TD_V_V__
C
Percent identity values for comparisons between NT-Als sequences in each protein
| | MgAls673 | MgAls2302 | MgAls3259 | MgAls3330 | CaAls3 |
| --- | --- | --- | --- | --- | --- |
| MgAls673 | 100 | -- | -- | -- | -- |
| MgAls2302 | 28 | 100 | -- | -- | -- |
| MgAls3259 | 28 | 57 | 100 | -- | -- |
| MgAls3330 | 30 | 35 | 35 | 100 | -- |
| CaAls3 | 28 | 36 | 34 | 41 | 100 |

## Slide 2
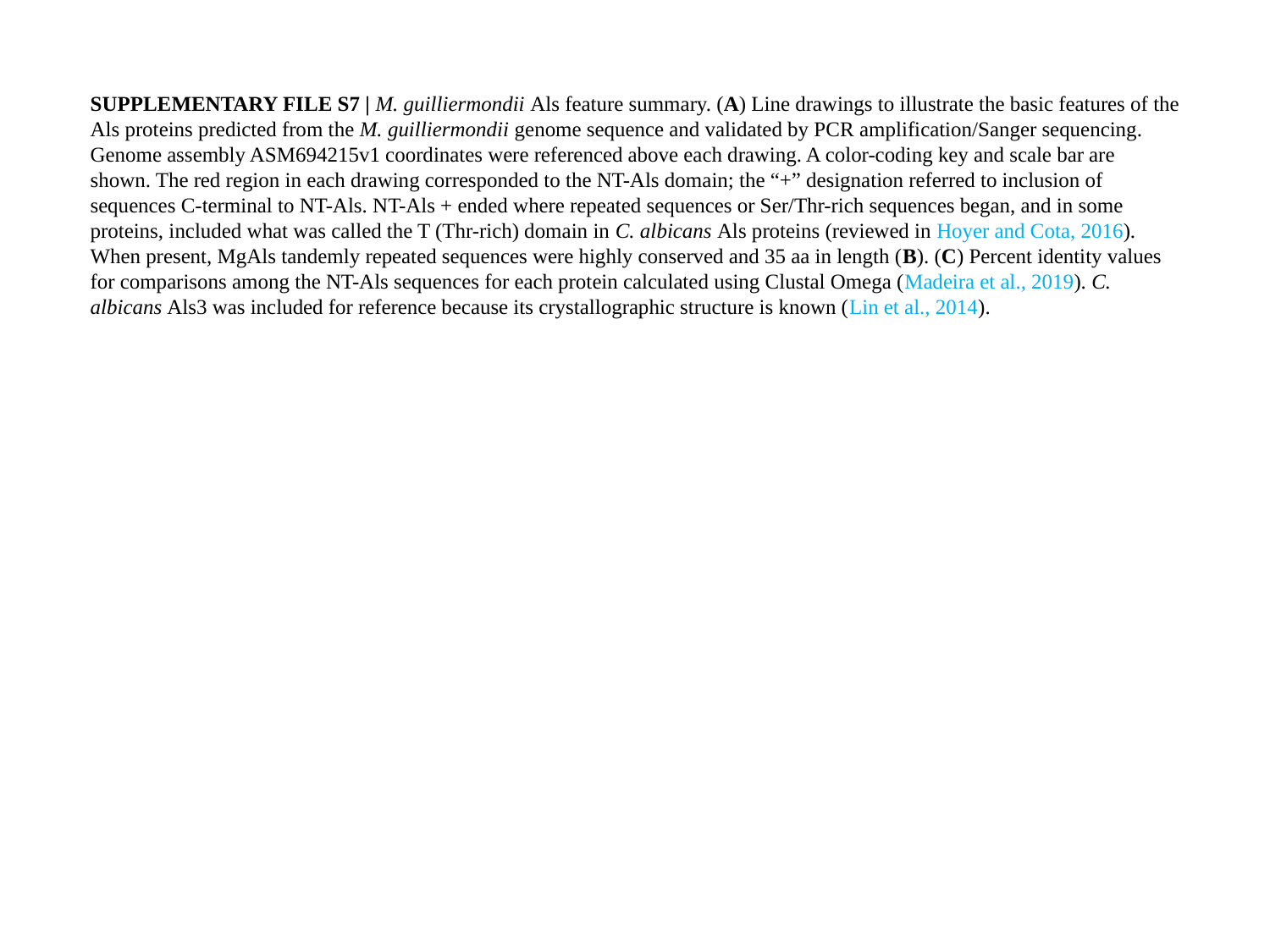

SUPPLEMENTARY FILE S7 | M. guilliermondii Als feature summary. (A) Line drawings to illustrate the basic features of the Als proteins predicted from the M. guilliermondii genome sequence and validated by PCR amplification/Sanger sequencing. Genome assembly ASM694215v1 coordinates were referenced above each drawing. A color-coding key and scale bar are shown. The red region in each drawing corresponded to the NT-Als domain; the “+” designation referred to inclusion of sequences C-terminal to NT-Als. NT-Als + ended where repeated sequences or Ser/Thr-rich sequences began, and in some proteins, included what was called the T (Thr-rich) domain in C. albicans Als proteins (reviewed in Hoyer and Cota, 2016). When present, MgAls tandemly repeated sequences were highly conserved and 35 aa in length (B). (C) Percent identity values for comparisons among the NT-Als sequences for each protein calculated using Clustal Omega (Madeira et al., 2019). C. albicans Als3 was included for reference because its crystallographic structure is known (Lin et al., 2014).
