# Supplementary material for: Using Genomics to Shape the Definition of the Agglutinin-Like Sequence (ALS) Family in the Saccharomycetales
Source: Front Cell Infect Microbiol. 2021 Dec 14;11:794529. doi: 10.3389/fcimb.2021.794529 (PMC8712946; doi:10.3389/fcimb.2021.794529)
Supplement: Supplementary file 8 [file Presentation_3.pptx]

## Slide 1
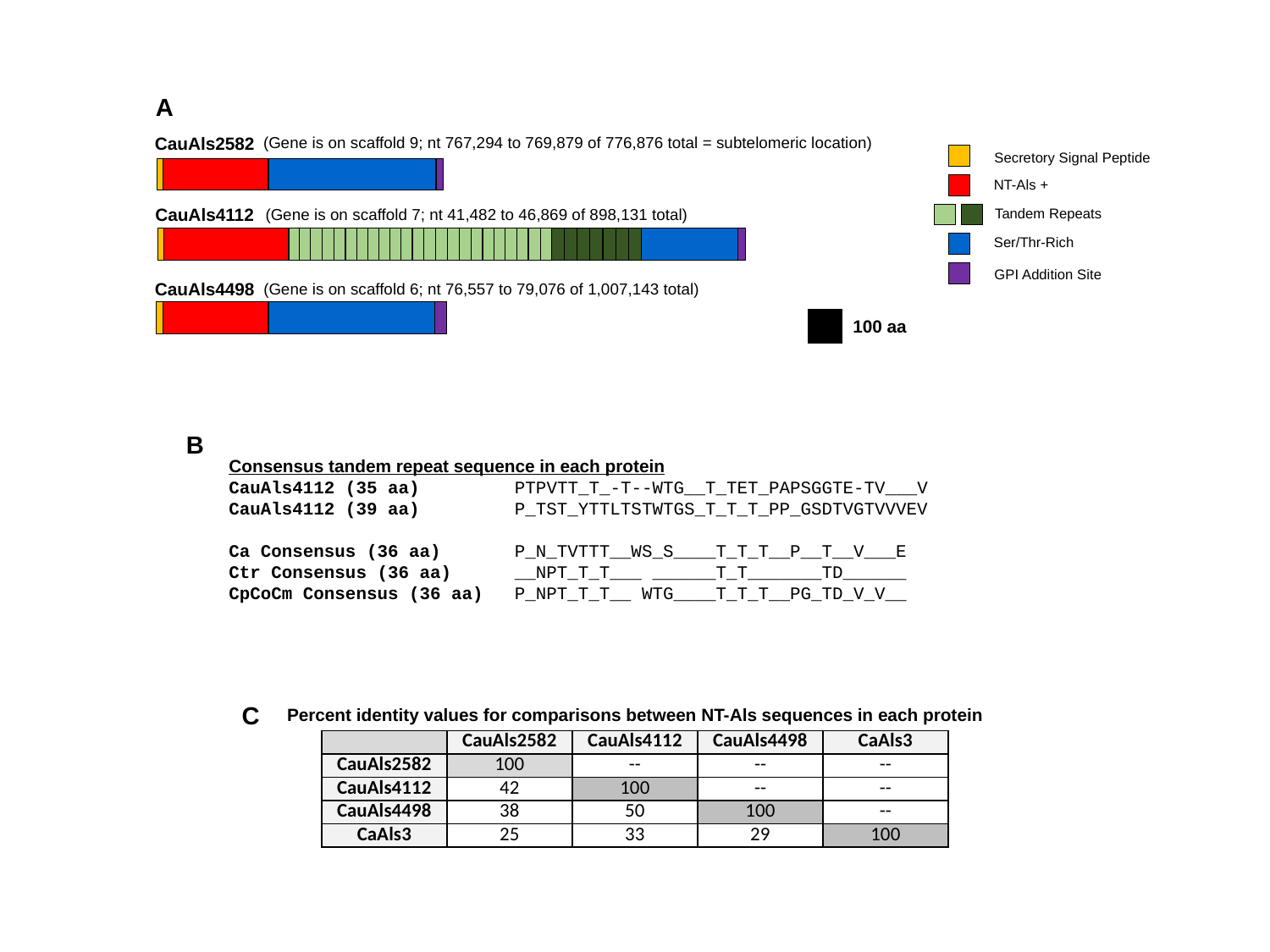

A
CauAls2582
(Gene is on scaffold 9; nt 767,294 to 769,879 of 776,876 total = subtelomeric location)
CauAls4112
(Gene is on scaffold 7; nt 41,482 to 46,869 of 898,131 total)
CauAls4498
(Gene is on scaffold 6; nt 76,557 to 79,076 of 1,007,143 total)
Secretory Signal Peptide
NT-Als +
Tandem Repeats
Ser/Thr-Rich
GPI Addition Site
100 aa
B
Consensus tandem repeat sequence in each protein
CauAls4112 (35 aa) PTPVTT_T_-T--WTG__T_TET_PAPSGGTE-TV___V
CauAls4112 (39 aa) P_TST_YTTLTSTWTGS_T_T_T_PP_GSDTVGTVVVEV
Ca Consensus (36 aa) P_N_TVTTT__WS_S____T_T_T__P__T__V___E
Ctr Consensus (36 aa) __NPT_T_T___ ______T_T_______TD______
CpCoCm Consensus (36 aa) P_NPT_T_T__ WTG____T_T_T__PG_TD_V_V__
C
Percent identity values for comparisons between NT-Als sequences in each protein
| | CauAls2582 | CauAls4112 | CauAls4498 | CaAls3 |
| --- | --- | --- | --- | --- |
| CauAls2582 | 100 | -- | -- | -- |
| CauAls4112 | 42 | 100 | -- | -- |
| CauAls4498 | 38 | 50 | 100 | -- |
| CaAls3 | 25 | 33 | 29 | 100 |

## Slide 2
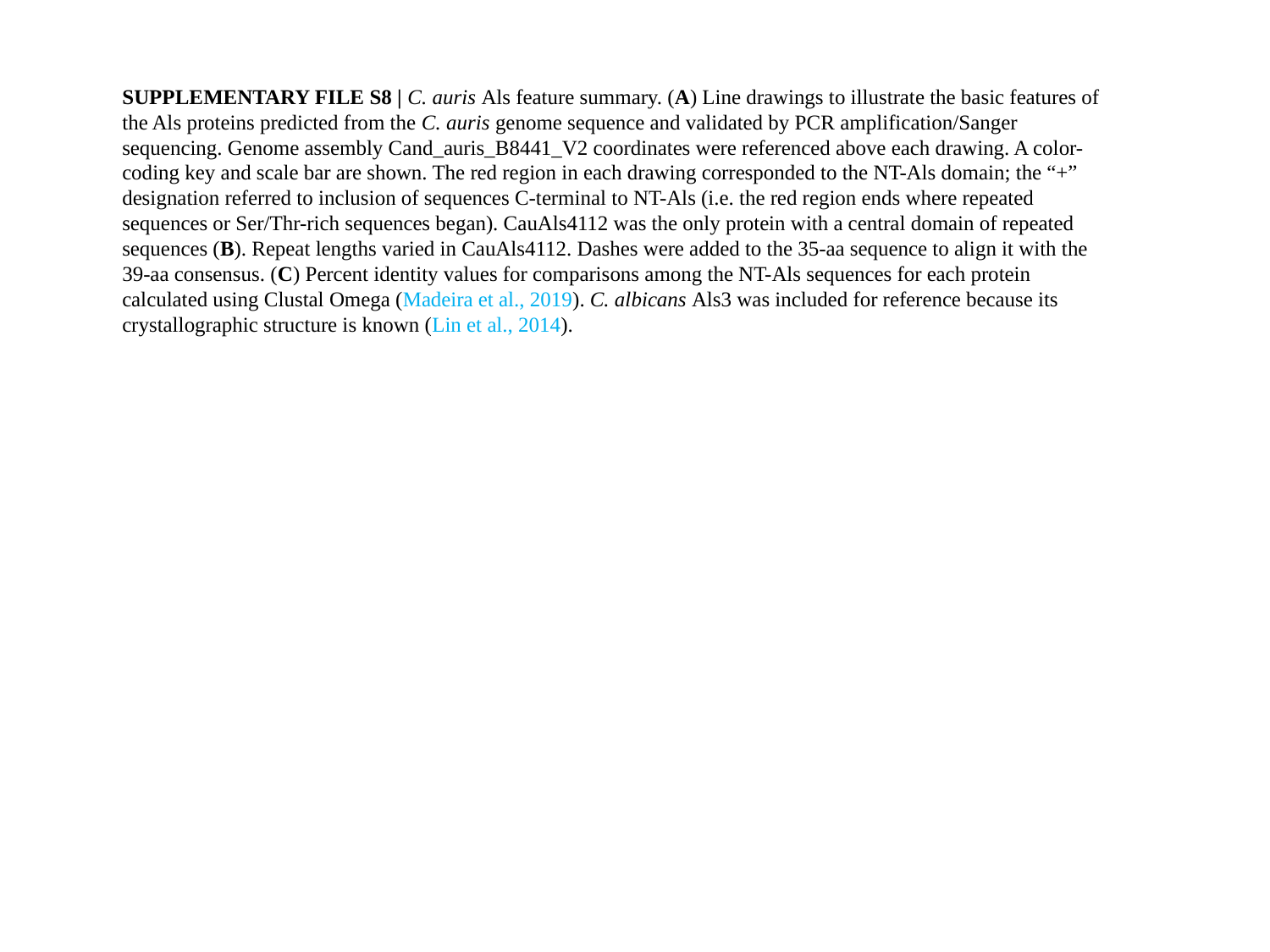

SUPPLEMENTARY FILE S8 | C. auris Als feature summary. (A) Line drawings to illustrate the basic features of the Als proteins predicted from the C. auris genome sequence and validated by PCR amplification/Sanger sequencing. Genome assembly Cand_auris_B8441_V2 coordinates were referenced above each drawing. A color-coding key and scale bar are shown. The red region in each drawing corresponded to the NT-Als domain; the “+” designation referred to inclusion of sequences C-terminal to NT-Als (i.e. the red region ends where repeated sequences or Ser/Thr-rich sequences began). CauAls4112 was the only protein with a central domain of repeated sequences (B). Repeat lengths varied in CauAls4112. Dashes were added to the 35-aa sequence to align it with the 39-aa consensus. (C) Percent identity values for comparisons among the NT-Als sequences for each protein calculated using Clustal Omega (Madeira et al., 2019). C. albicans Als3 was included for reference because its crystallographic structure is known (Lin et al., 2014).
