# Supplementary material for: Using Genomics to Shape the Definition of the Agglutinin-Like Sequence (ALS) Family in the Saccharomycetales
Source: Front Cell Infect Microbiol. 2021 Dec 14;11:794529. doi: 10.3389/fcimb.2021.794529 (PMC8712946; doi:10.3389/fcimb.2021.794529)
Supplement: Supplementary file 9 [file Presentation_4.pptx]

## Slide 1
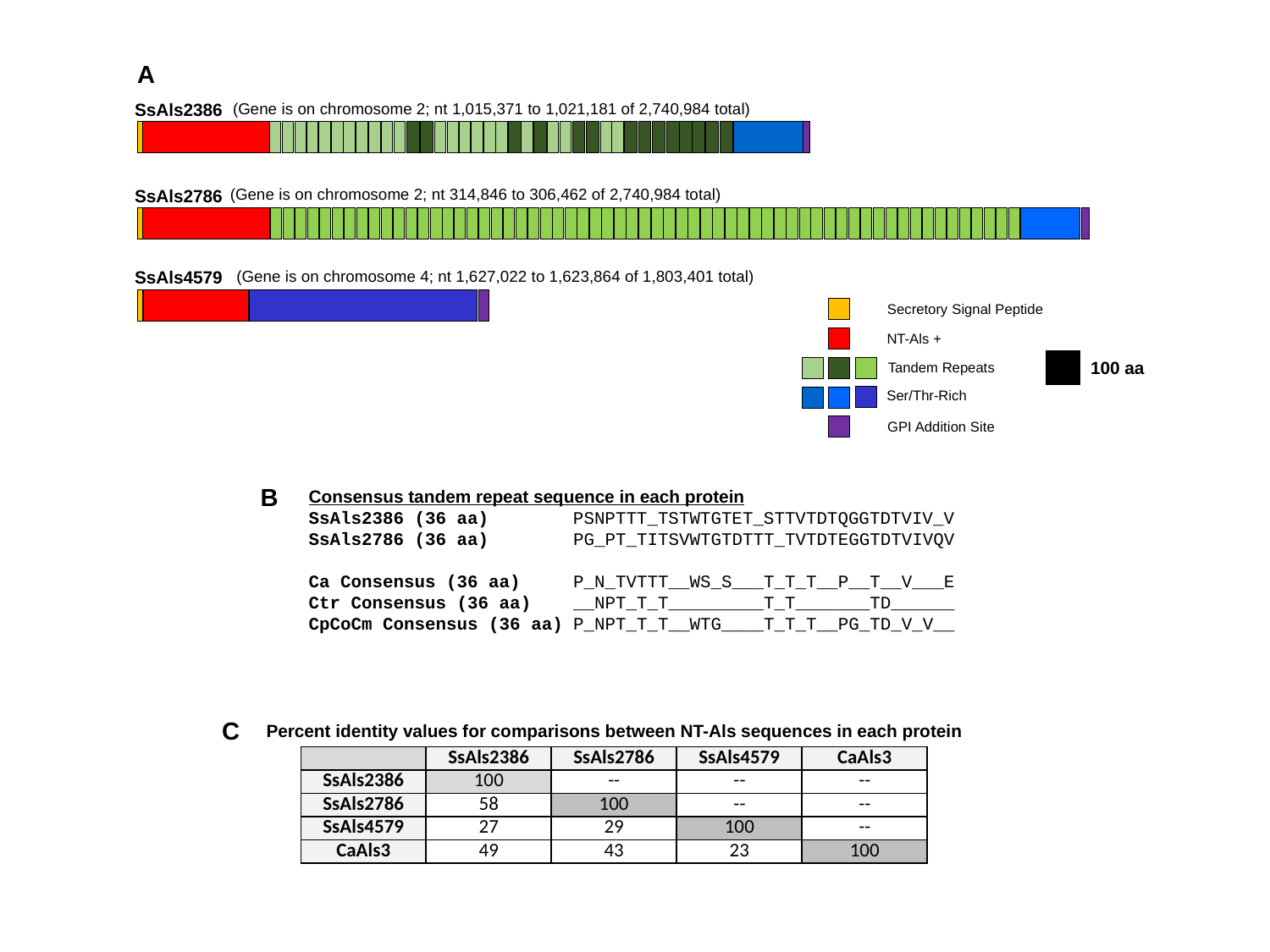

A
SsAls2386
(Gene is on chromosome 2; nt 1,015,371 to 1,021,181 of 2,740,984 total)
(Gene is on chromosome 2; nt 314,846 to 306,462 of 2,740,984 total)
SsAls2786
SsAls4579
(Gene is on chromosome 4; nt 1,627,022 to 1,623,864 of 1,803,401 total)
Secretory Signal Peptide
NT-Als +
Tandem Repeats
Ser/Thr-Rich
GPI Addition Site
100 aa
B
Consensus tandem repeat sequence in each protein
SsAls2386 (36 aa) PSNPTTT_TSTWTGTET_STTVTDTQGGTDTVIV_V
SsAls2786 (36 aa) PG_PT_TITSVWTGTDTTT_TVTDTEGGTDTVIVQV
Ca Consensus (36 aa) P_N_TVTTT__WS_S___T_T_T__P__T__V___E
Ctr Consensus (36 aa) __NPT_T_T_________T_T_______TD______
CpCoCm Consensus (36 aa) P_NPT_T_T__WTG____T_T_T__PG_TD_V_V__
C
Percent identity values for comparisons between NT-Als sequences in each protein
| | SsAls2386 | SsAls2786 | SsAls4579 | CaAls3 |
| --- | --- | --- | --- | --- |
| SsAls2386 | 100 | -- | -- | -- |
| SsAls2786 | 58 | 100 | -- | -- |
| SsAls4579 | 27 | 29 | 100 | -- |
| CaAls3 | 49 | 43 | 23 | 100 |

## Slide 2
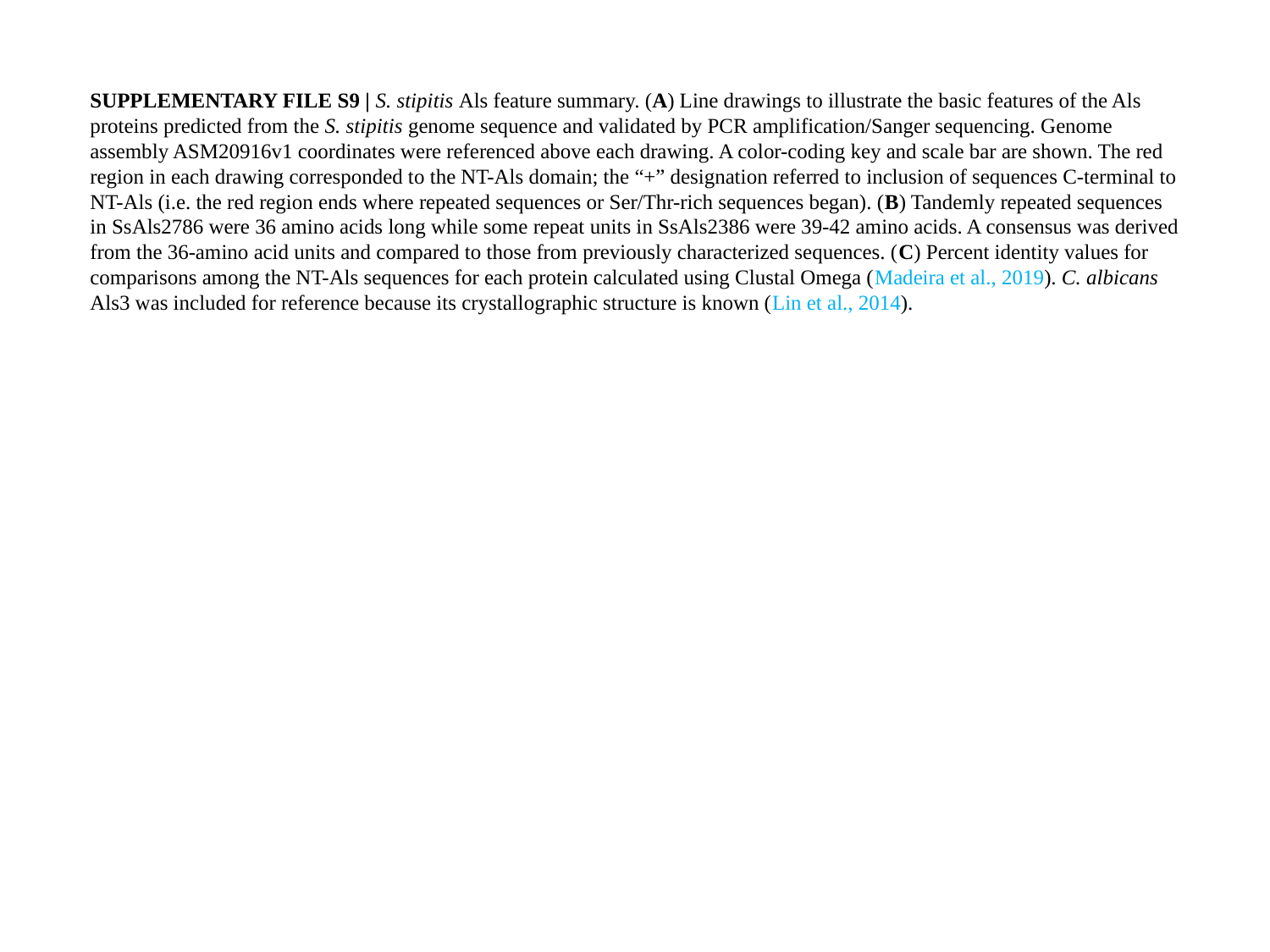

SUPPLEMENTARY FILE S9 | S. stipitis Als feature summary. (A) Line drawings to illustrate the basic features of the Als proteins predicted from the S. stipitis genome sequence and validated by PCR amplification/Sanger sequencing. Genome assembly ASM20916v1 coordinates were referenced above each drawing. A color-coding key and scale bar are shown. The red region in each drawing corresponded to the NT-Als domain; the “+” designation referred to inclusion of sequences C-terminal to NT-Als (i.e. the red region ends where repeated sequences or Ser/Thr-rich sequences began). (B) Tandemly repeated sequences in SsAls2786 were 36 amino acids long while some repeat units in SsAls2386 were 39-42 amino acids. A consensus was derived from the 36-amino acid units and compared to those from previously characterized sequences. (C) Percent identity values for comparisons among the NT-Als sequences for each protein calculated using Clustal Omega (Madeira et al., 2019). C. albicans Als3 was included for reference because its crystallographic structure is known (Lin et al., 2014).
