# Supplementary material for: Using Genomics to Shape the Definition of the Agglutinin-Like Sequence (ALS) Family in the Saccharomycetales
Source: Front Cell Infect Microbiol. 2021 Dec 14;11:794529. doi: 10.3389/fcimb.2021.794529 (PMC8712946; doi:10.3389/fcimb.2021.794529)
Supplement: Supplementary file 10 [file Presentation_5.pptx]

## Slide 1
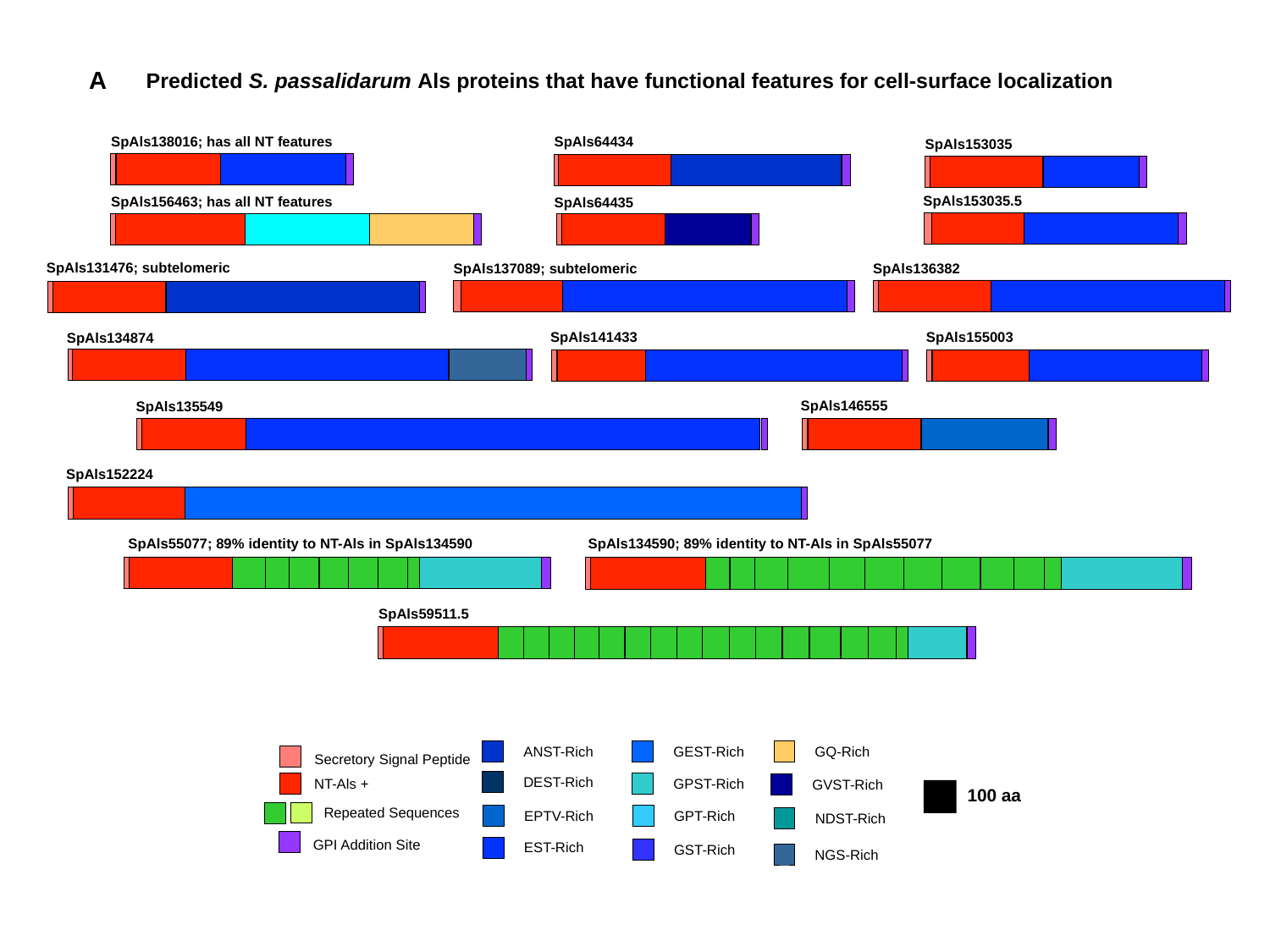

A
Predicted S. passalidarum Als proteins that have functional features for cell-surface localization
SpAls138016; has all NT features
SpAls156463; has all NT features
SpAls64434
SpAls64435
SpAls153035
SpAls153035.5
SpAls131476; subtelomeric
SpAls137089; subtelomeric
SpAls136382
SpAls141433
SpAls155003
SpAls134874
SpAls146555
SpAls135549
SpAls152224
SpAls134590; 89% identity to NT-Als in SpAls55077
SpAls55077; 89% identity to NT-Als in SpAls134590
SpAls59511.5
ANST-Rich
GEST-Rich
GQ-Rich
Secretory Signal Peptide
DEST-Rich
NT-Als +
GPST-Rich
GVST-Rich
Repeated Sequences
EPTV-Rich
GPT-Rich
NDST-Rich
GPI Addition Site
EST-Rich
GST-Rich
NGS-Rich
100 aa

## Slide 2
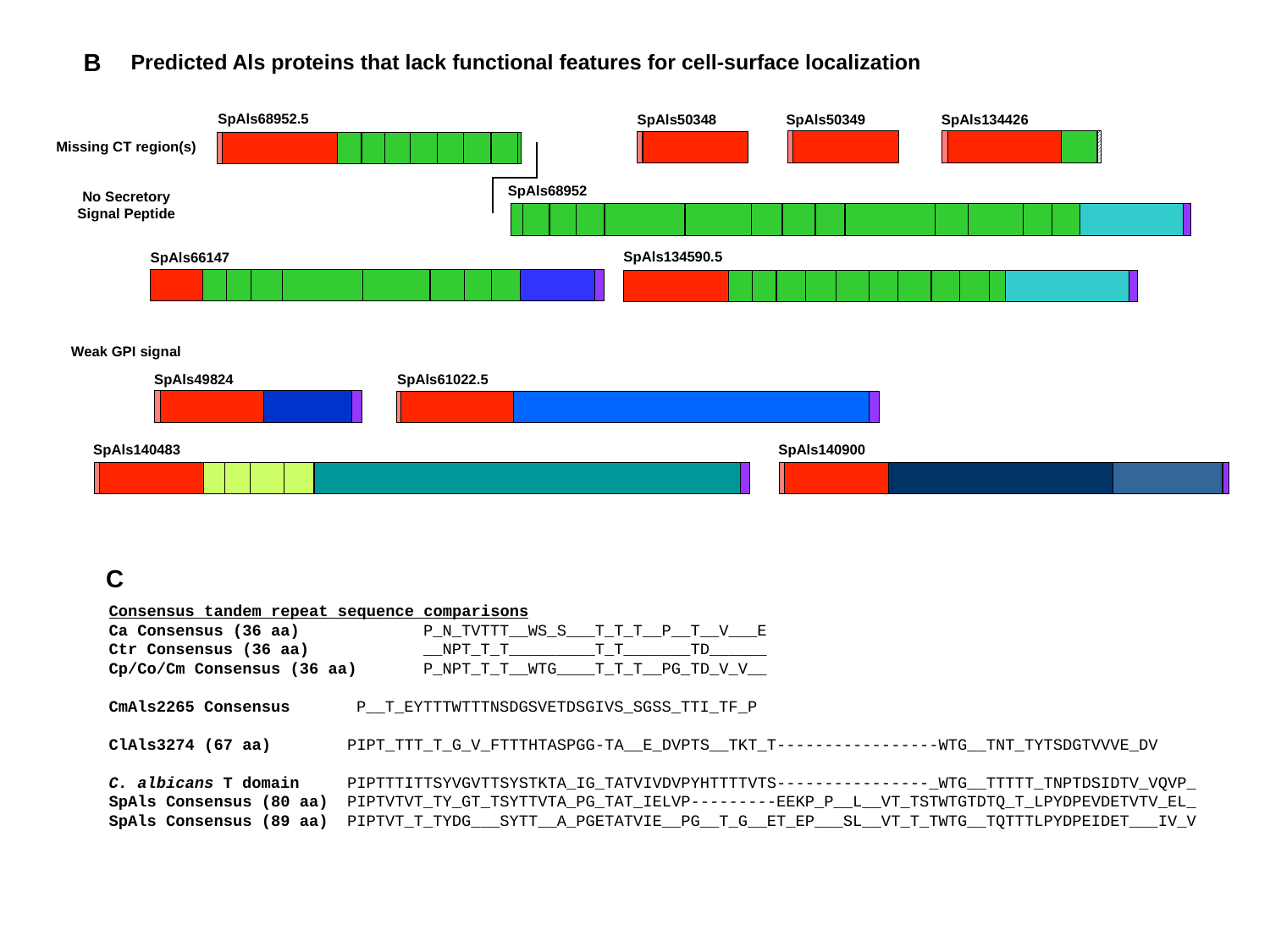

B
Predicted Als proteins that lack functional features for cell-surface localization
SpAls68952.5
SpAls50348
SpAls134426
SpAls50349
Missing CT region(s)
SpAls68952
No Secretory
Signal Peptide
SpAls134590.5
SpAls66147
Weak GPI signal
SpAls61022.5
SpAls49824
SpAls140900
SpAls140483
C
Consensus tandem repeat sequence comparisons
Ca Consensus (36 aa) P_N_TVTTT__WS_S___T_T_T__P__T__V___E
Ctr Consensus (36 aa) __NPT_T_T_________T_T_______TD______
Cp/Co/Cm Consensus (36 aa) P_NPT_T_T__WTG____T_T_T__PG_TD_V_V__
CmAls2265 Consensus P__T_EYTTTWTTTNSDGSVETDSGIVS_SGSS_TTI_TF_P
ClAls3274 (67 aa) PIPT_TTT_T_G_V_FTTTHTASPGG-TA__E_DVPTS__TKT_T-----------------WTG__TNT_TYTSDGTVVVE_DV
C. albicans T domain PIPTTTITTSYVGVTTSYSTKTA_IG_TATVIVDVPYHTTTTVTS----------------_WTG__TTTTT_TNPTDSIDTV_VQVP_
SpAls Consensus (80 aa) PIPTVTVT_TY_GT_TSYTTVTA_PG_TAT_IELVP---------EEKP_P__L__VT_TSTWTGTDTQ_T_LPYDPEVDETVTV_EL_
SpAls Consensus (89 aa) PIPTVT_T_TYDG___SYTT__A_PGETATVIE__PG__T_G__ET_EP___SL__VT_T_TWTG__TQTTTLPYDPEIDET___IV_V

## Slide 3
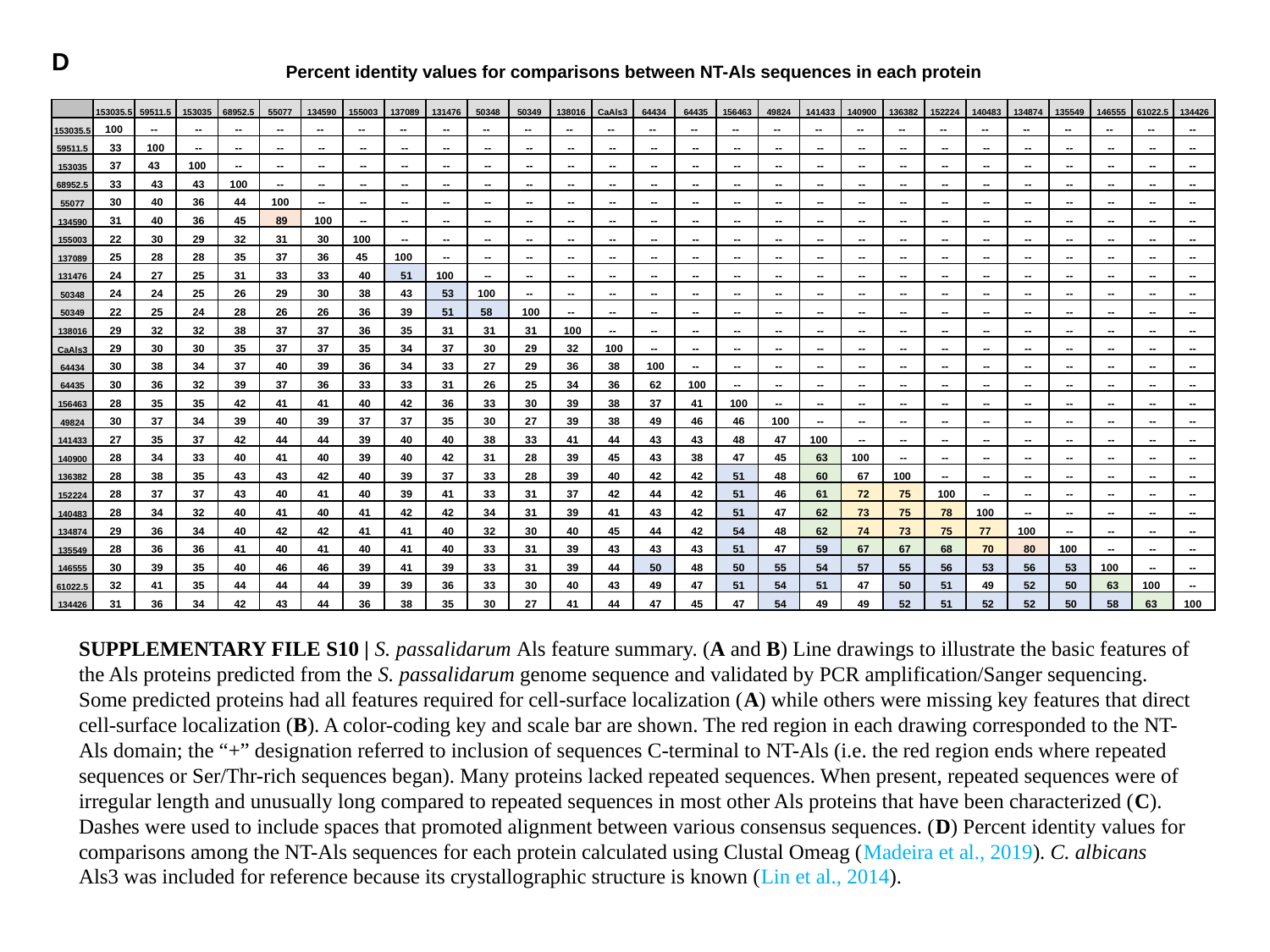

D
Percent identity values for comparisons between NT-Als sequences in each protein
| | 153035.5 | 59511.5 | 153035 | 68952.5 | 55077 | 134590 | 155003 | 137089 | 131476 | 50348 | 50349 | 138016 | CaAls3 | 64434 | 64435 | 156463 | 49824 | 141433 | 140900 | 136382 | 152224 | 140483 | 134874 | 135549 | 146555 | 61022.5 | 134426 |
| --- | --- | --- | --- | --- | --- | --- | --- | --- | --- | --- | --- | --- | --- | --- | --- | --- | --- | --- | --- | --- | --- | --- | --- | --- | --- | --- | --- |
| 153035.5 | 100 | -- | -- | -- | -- | -- | -- | -- | -- | -- | -- | -- | -- | -- | -- | -- | -- | -- | -- | -- | -- | -- | -- | -- | -- | -- | -- |
| 59511.5 | 33 | 100 | -- | -- | -- | -- | -- | -- | -- | -- | -- | -- | -- | -- | -- | -- | -- | -- | -- | -- | -- | -- | -- | -- | -- | -- | -- |
| 153035 | 37 | 43 | 100 | -- | -- | -- | -- | -- | -- | -- | -- | -- | -- | -- | -- | -- | -- | -- | -- | -- | -- | -- | -- | -- | -- | -- | -- |
| 68952.5 | 33 | 43 | 43 | 100 | -- | -- | -- | -- | -- | -- | -- | -- | -- | -- | -- | -- | -- | -- | -- | -- | -- | -- | -- | -- | -- | -- | -- |
| 55077 | 30 | 40 | 36 | 44 | 100 | -- | -- | -- | -- | -- | -- | -- | -- | -- | -- | -- | -- | -- | -- | -- | -- | -- | -- | -- | -- | -- | -- |
| 134590 | 31 | 40 | 36 | 45 | 89 | 100 | -- | -- | -- | -- | -- | -- | -- | -- | -- | -- | -- | -- | -- | -- | -- | -- | -- | -- | -- | -- | -- |
| 155003 | 22 | 30 | 29 | 32 | 31 | 30 | 100 | -- | -- | -- | -- | -- | -- | -- | -- | -- | -- | -- | -- | -- | -- | -- | -- | -- | -- | -- | -- |
| 137089 | 25 | 28 | 28 | 35 | 37 | 36 | 45 | 100 | -- | -- | -- | -- | -- | -- | -- | -- | -- | -- | -- | -- | -- | -- | -- | -- | -- | -- | -- |
| 131476 | 24 | 27 | 25 | 31 | 33 | 33 | 40 | 51 | 100 | -- | -- | -- | -- | -- | -- | -- | -- | -- | -- | -- | -- | -- | -- | -- | -- | -- | -- |
| 50348 | 24 | 24 | 25 | 26 | 29 | 30 | 38 | 43 | 53 | 100 | -- | -- | -- | -- | -- | -- | -- | -- | -- | -- | -- | -- | -- | -- | -- | -- | -- |
| 50349 | 22 | 25 | 24 | 28 | 26 | 26 | 36 | 39 | 51 | 58 | 100 | -- | -- | -- | -- | -- | -- | -- | -- | -- | -- | -- | -- | -- | -- | -- | -- |
| 138016 | 29 | 32 | 32 | 38 | 37 | 37 | 36 | 35 | 31 | 31 | 31 | 100 | -- | -- | -- | -- | -- | -- | -- | -- | -- | -- | -- | -- | -- | -- | -- |
| CaAls3 | 29 | 30 | 30 | 35 | 37 | 37 | 35 | 34 | 37 | 30 | 29 | 32 | 100 | -- | -- | -- | -- | -- | -- | -- | -- | -- | -- | -- | -- | -- | -- |
| 64434 | 30 | 38 | 34 | 37 | 40 | 39 | 36 | 34 | 33 | 27 | 29 | 36 | 38 | 100 | -- | -- | -- | -- | -- | -- | -- | -- | -- | -- | -- | -- | -- |
| 64435 | 30 | 36 | 32 | 39 | 37 | 36 | 33 | 33 | 31 | 26 | 25 | 34 | 36 | 62 | 100 | -- | -- | -- | -- | -- | -- | -- | -- | -- | -- | -- | -- |
| 156463 | 28 | 35 | 35 | 42 | 41 | 41 | 40 | 42 | 36 | 33 | 30 | 39 | 38 | 37 | 41 | 100 | -- | -- | -- | -- | -- | -- | -- | -- | -- | -- | -- |
| 49824 | 30 | 37 | 34 | 39 | 40 | 39 | 37 | 37 | 35 | 30 | 27 | 39 | 38 | 49 | 46 | 46 | 100 | -- | -- | -- | -- | -- | -- | -- | -- | -- | -- |
| 141433 | 27 | 35 | 37 | 42 | 44 | 44 | 39 | 40 | 40 | 38 | 33 | 41 | 44 | 43 | 43 | 48 | 47 | 100 | -- | -- | -- | -- | -- | -- | -- | -- | -- |
| 140900 | 28 | 34 | 33 | 40 | 41 | 40 | 39 | 40 | 42 | 31 | 28 | 39 | 45 | 43 | 38 | 47 | 45 | 63 | 100 | -- | -- | -- | -- | -- | -- | -- | -- |
| 136382 | 28 | 38 | 35 | 43 | 43 | 42 | 40 | 39 | 37 | 33 | 28 | 39 | 40 | 42 | 42 | 51 | 48 | 60 | 67 | 100 | -- | -- | -- | -- | -- | -- | -- |
| 152224 | 28 | 37 | 37 | 43 | 40 | 41 | 40 | 39 | 41 | 33 | 31 | 37 | 42 | 44 | 42 | 51 | 46 | 61 | 72 | 75 | 100 | -- | -- | -- | -- | -- | -- |
| 140483 | 28 | 34 | 32 | 40 | 41 | 40 | 41 | 42 | 42 | 34 | 31 | 39 | 41 | 43 | 42 | 51 | 47 | 62 | 73 | 75 | 78 | 100 | -- | -- | -- | -- | -- |
| 134874 | 29 | 36 | 34 | 40 | 42 | 42 | 41 | 41 | 40 | 32 | 30 | 40 | 45 | 44 | 42 | 54 | 48 | 62 | 74 | 73 | 75 | 77 | 100 | -- | -- | -- | -- |
| 135549 | 28 | 36 | 36 | 41 | 40 | 41 | 40 | 41 | 40 | 33 | 31 | 39 | 43 | 43 | 43 | 51 | 47 | 59 | 67 | 67 | 68 | 70 | 80 | 100 | -- | -- | -- |
| 146555 | 30 | 39 | 35 | 40 | 46 | 46 | 39 | 41 | 39 | 33 | 31 | 39 | 44 | 50 | 48 | 50 | 55 | 54 | 57 | 55 | 56 | 53 | 56 | 53 | 100 | -- | -- |
| 61022.5 | 32 | 41 | 35 | 44 | 44 | 44 | 39 | 39 | 36 | 33 | 30 | 40 | 43 | 49 | 47 | 51 | 54 | 51 | 47 | 50 | 51 | 49 | 52 | 50 | 63 | 100 | -- |
| 134426 | 31 | 36 | 34 | 42 | 43 | 44 | 36 | 38 | 35 | 30 | 27 | 41 | 44 | 47 | 45 | 47 | 54 | 49 | 49 | 52 | 51 | 52 | 52 | 50 | 58 | 63 | 100 |
SUPPLEMENTARY FILE S10 | S. passalidarum Als feature summary. (A and B) Line drawings to illustrate the basic features of the Als proteins predicted from the S. passalidarum genome sequence and validated by PCR amplification/Sanger sequencing. Some predicted proteins had all features required for cell-surface localization (A) while others were missing key features that direct cell-surface localization (B). A color-coding key and scale bar are shown. The red region in each drawing corresponded to the NT-Als domain; the “+” designation referred to inclusion of sequences C-terminal to NT-Als (i.e. the red region ends where repeated sequences or Ser/Thr-rich sequences began). Many proteins lacked repeated sequences. When present, repeated sequences were of irregular length and unusually long compared to repeated sequences in most other Als proteins that have been characterized (C). Dashes were used to include spaces that promoted alignment between various consensus sequences. (D) Percent identity values for comparisons among the NT-Als sequences for each protein calculated using Clustal Omeag (Madeira et al., 2019). C. albicans Als3 was included for reference because its crystallographic structure is known (Lin et al., 2014).
