# Supplementary material for: Using Genomics to Shape the Definition of the Agglutinin-Like Sequence (ALS) Family in the Saccharomycetales
Source: Front Cell Infect Microbiol. 2021 Dec 14;11:794529. doi: 10.3389/fcimb.2021.794529 (PMC8712946; doi:10.3389/fcimb.2021.794529)
Supplement: Supplementary file 11 [file Presentation_6.pptx]

## Slide 1
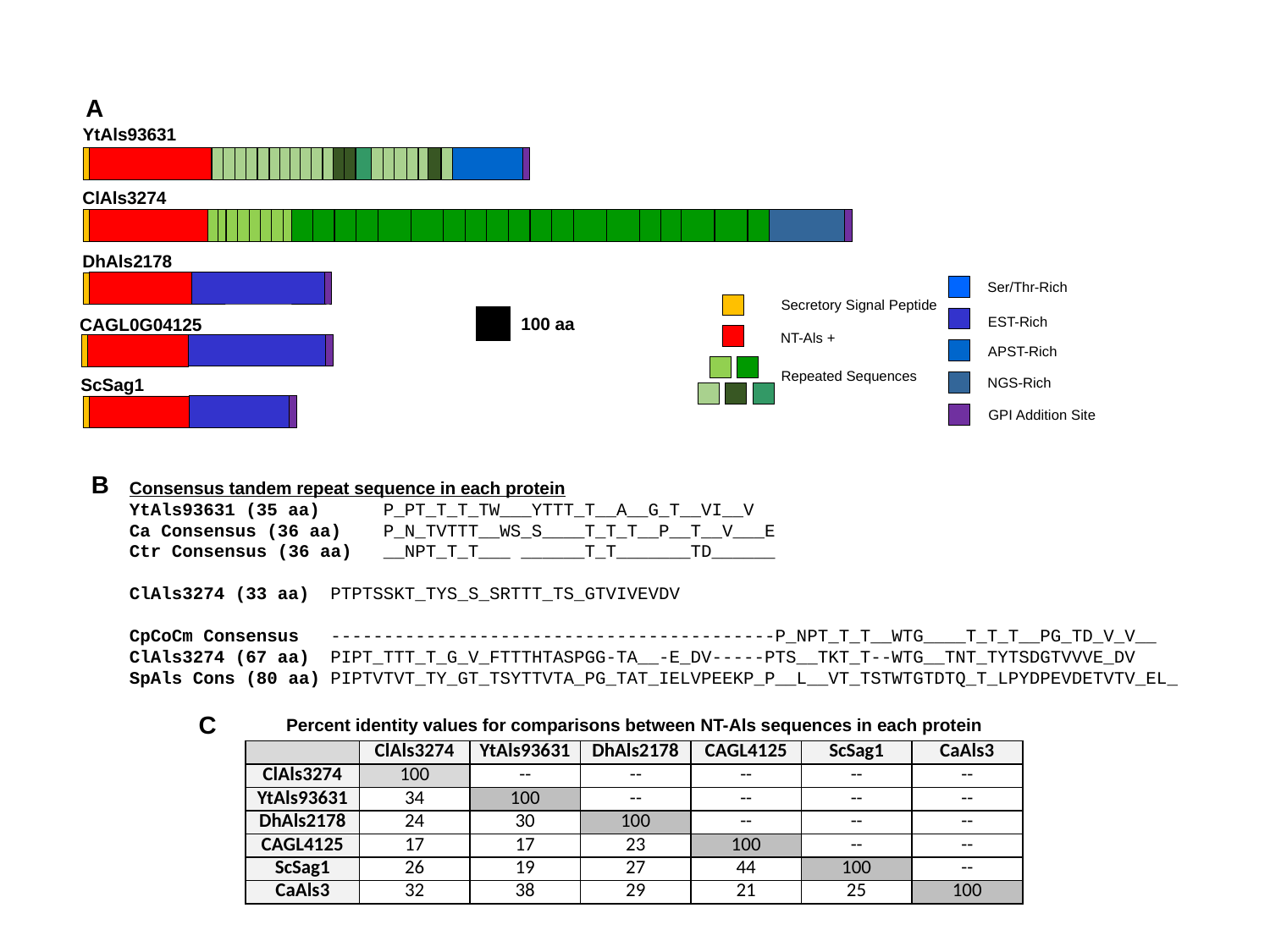

A
YtAls93631
ClAls3274
DhAls2178
Ser/Thr-Rich
Secretory Signal Peptide
EST-Rich
NT-Als +
APST-Rich
Repeated Sequences
NGS-Rich
GPI Addition Site
100 aa
CAGL0G04125
ScSag1
B
Consensus tandem repeat sequence in each protein
YtAls93631 (35 aa) P_PT_T_T_TW___YTTT_T__A__G_T__VI__V
Ca Consensus (36 aa) P_N_TVTTT__WS_S____T_T_T__P__T__V___E
Ctr Consensus (36 aa) __NPT_T_T___ ______T_T_______TD______
ClAls3274 (33 aa) PTPTSSKT_TYS_S_SRTTT_TS_GTVIVEVDV
CpCoCm Consensus ------------------------------------------P_NPT_T_T__WTG____T_T_T__PG_TD_V_V__
ClAls3274 (67 aa) PIPT_TTT_T_G_V_FTTTHTASPGG-TA__-E_DV-----PTS__TKT_T--WTG__TNT_TYTSDGTVVVE_DV
SpAls Cons (80 aa) PIPTVTVT_TY_GT_TSYTTVTA_PG_TAT_IELVPEEKP_P__L__VT_TSTWTGTDTQ_T_LPYDPEVDETVTV_EL_
C
Percent identity values for comparisons between NT-Als sequences in each protein
| | ClAls3274 | YtAls93631 | DhAls2178 | CAGL4125 | ScSag1 | CaAls3 |
| --- | --- | --- | --- | --- | --- | --- |
| ClAls3274 | 100 | -- | -- | -- | -- | -- |
| YtAls93631 | 34 | 100 | -- | -- | -- | -- |
| DhAls2178 | 24 | 30 | 100 | -- | -- | -- |
| CAGL4125 | 17 | 17 | 23 | 100 | -- | -- |
| ScSag1 | 26 | 19 | 27 | 44 | 100 | -- |
| CaAls3 | 32 | 38 | 29 | 21 | 25 | 100 |

## Slide 2
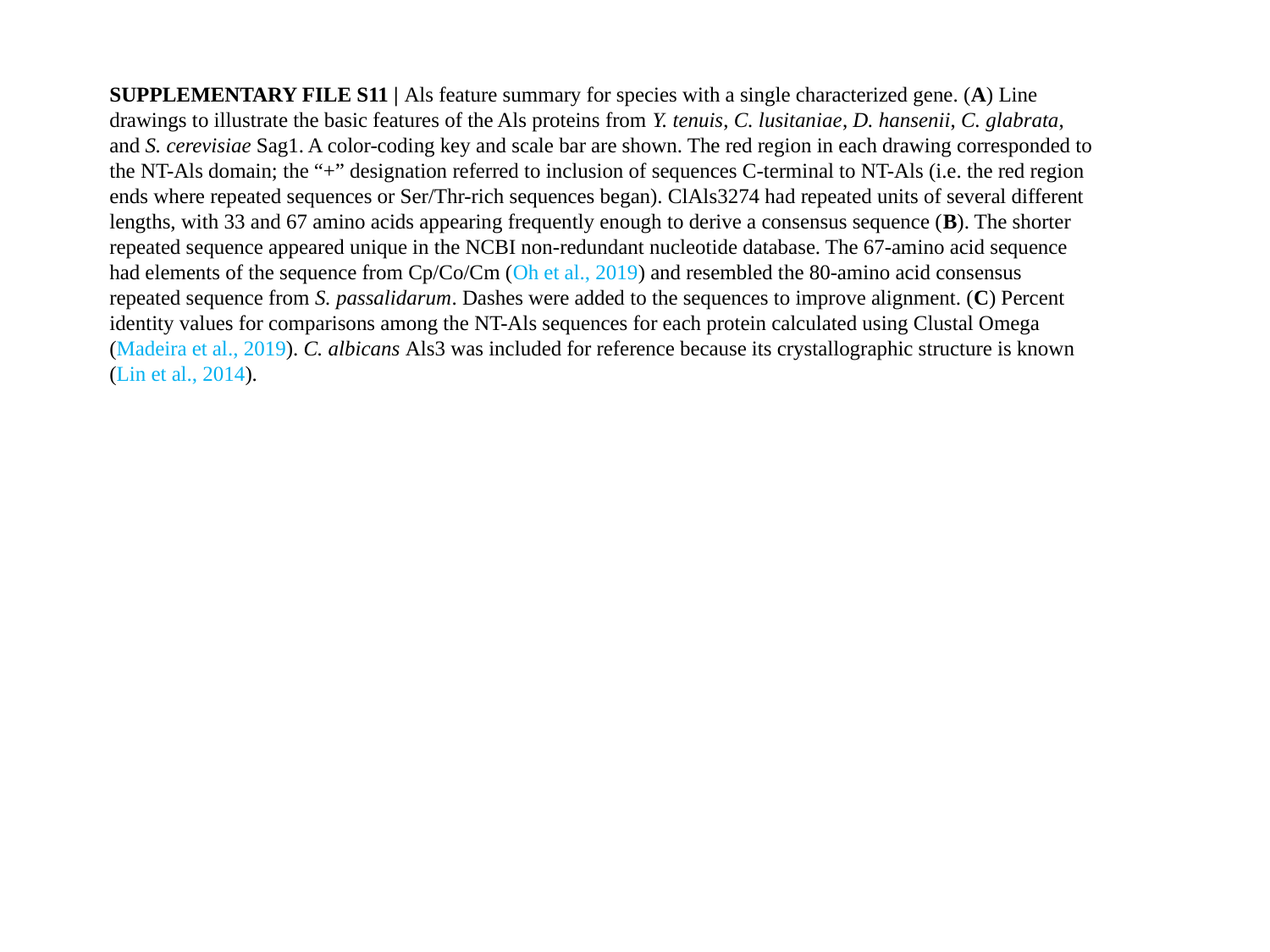

SUPPLEMENTARY FILE S11 | Als feature summary for species with a single characterized gene. (A) Line drawings to illustrate the basic features of the Als proteins from Y. tenuis, C. lusitaniae, D. hansenii, C. glabrata, and S. cerevisiae Sag1. A color-coding key and scale bar are shown. The red region in each drawing corresponded to the NT-Als domain; the “+” designation referred to inclusion of sequences C-terminal to NT-Als (i.e. the red region ends where repeated sequences or Ser/Thr-rich sequences began). ClAls3274 had repeated units of several different lengths, with 33 and 67 amino acids appearing frequently enough to derive a consensus sequence (B). The shorter repeated sequence appeared unique in the NCBI non-redundant nucleotide database. The 67-amino acid sequence had elements of the sequence from Cp/Co/Cm (Oh et al., 2019) and resembled the 80-amino acid consensus repeated sequence from S. passalidarum. Dashes were added to the sequences to improve alignment. (C) Percent identity values for comparisons among the NT-Als sequences for each protein calculated using Clustal Omega (Madeira et al., 2019). C. albicans Als3 was included for reference because its crystallographic structure is known (Lin et al., 2014).
