# Supplementary material for: Using Genomics to Shape the Definition of the Agglutinin-Like Sequence (ALS) Family in the Saccharomycetales
Source: Front Cell Infect Microbiol. 2021 Dec 14;11:794529. doi: 10.3389/fcimb.2021.794529 (PMC8712946; doi:10.3389/fcimb.2021.794529)
Supplement: Supplementary file 12 [file Presentation_7.pptx]

## Slide 1
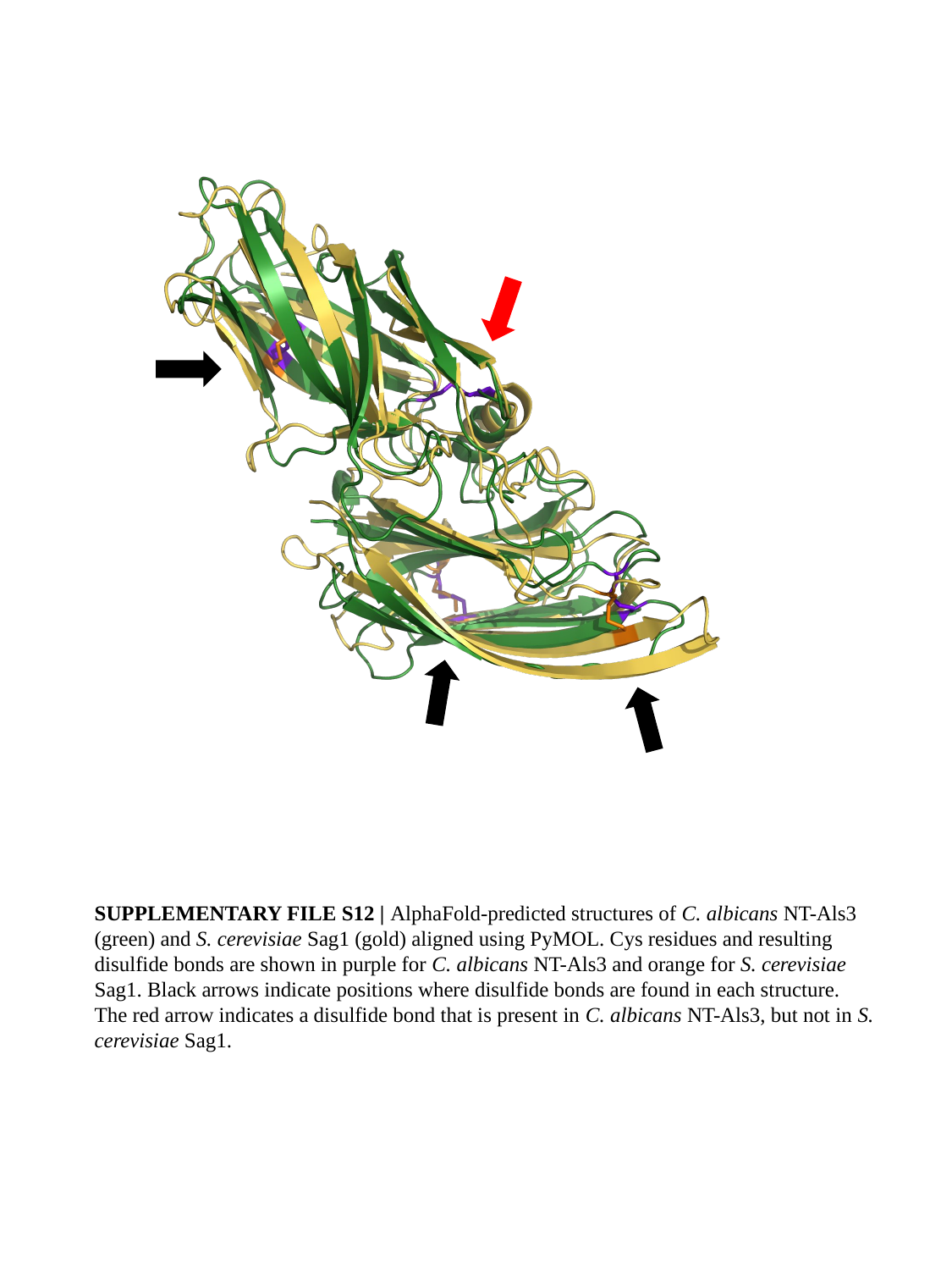

SUPPLEMENTARY FILE S12 | AlphaFold-predicted structures of C. albicans NT-Als3 (green) and S. cerevisiae Sag1 (gold) aligned using PyMOL. Cys residues and resulting disulfide bonds are shown in purple for C. albicans NT-Als3 and orange for S. cerevisiae Sag1. Black arrows indicate positions where disulfide bonds are found in each structure. The red arrow indicates a disulfide bond that is present in C. albicans NT-Als3, but not in S. cerevisiae Sag1.
