# Supplementary material for: Using Genomics to Shape the Definition of the Agglutinin-Like Sequence (ALS) Family in the Saccharomycetales
Source: Front Cell Infect Microbiol. 2021 Dec 14;11:794529. doi: 10.3389/fcimb.2021.794529 (PMC8712946; doi:10.3389/fcimb.2021.794529)
Supplement: Supplementary file 13 [file Table_1.docx]

**SUPPLEMENTARY TABLE S1 |** Oligonucleotide primers used for PCR amplification and DNA sequencing in this study.

| **Gene**  **(GenBank Accession)** | **Primer Name** | **Alias** | **Sequence (5’-3’)** | **Location*** |
| --- | --- | --- | --- | --- |
| *LeALS734*  (MN893368) | LeGene1-NT-F1 | Le1-1F | TGCTGATACTCACGTCTTGAT | -195 to -175 |
|  | LeGene1-TR-F1 | Le1-3F | CATATTGTGGTGCGCAAGTAT | 1136 to 1156 |
|  | LeGene1-NT-R1 | Le1-2R | GTTGTGAGAGTGACTGTTGATATT | 1317 to 1339 |
|  | LeGene1-CT-Sq-F1 | Le1-13F | CCCCGTAATGAAAATTTAAGTAGC | 1837 to 1860 |
|  | LeGene1-CT-Sq-R2 | Le1-12R | TTCCTCTGTCAAAAACTCGCTTG | 1913 to 1935 |
|  | LeGene1-CT-R1 | Le1-6R | CACGTCATCATCGGAC**^1^**TAAAAC | 3253 to 3276 |
|  | LeGene1-CT-R2 | Le1-9R | AGCAAGTACACTATCGGTCAAATG | +233 to +256 |
| *LeALS2536*  (MN893369) | LeGene2-NT-F1 | Le2-1F | GGAATGGTTGATGTGATGTTTCTT | -135 to -112 |
|  | LeGene2-TR-F1 | Le2-3F | ATACAGCCTTGTGTCCAATC | 1208 to 1227 |
|  | LeGene2-NT-R1 | Le2-2R | TCGCTGGTAGTAGGCTCTT | 1361 to 1379 |
|  | LeGene2-TR-R1 | Le2-4R | CAGTATCAGTGGGCGGAAC | 2911 to 2929 |
|  | LeGene2-TR-Sq-F1 | Le2-20F | GTTCCGCCCACTGATACTG | 2911 to 2929 |
|  | LeGene2-CT-Sq-R1 | Le2-6R | GCTGAAGGTGTAGCGGTATT | 4291 to 4310 |
|  | LeGene2-CT-R1 | Le2-7R | AATCTGTCAATATCACTTTCGTTG | +70 to +90 |
| *LeALS2716*  (MN893370) | LeGene3-NT-F1 | Le3-1F | TGCACGTGTTGGCTGAAA | -134 to -117 |
|  | LeGene3-TR-F1 | Le3-3F | GACGACTGTGACAGAACACTAT | 1104 to 1125 |
|  | LeGene3-NT-R1 | Le3-2R | CCTTGCCATCATCAGTAGTTGT | 1240 to 1261 |
|  | LeGene3-TR3-F1 | Le3-17F | CCCACAACCAGAAGAATCC | 2607 to 2625 |
|  | LeGene3-TR2-R1 | Le3-18R | GACAGTGAATTCATCAGTGGATTC | 2620 to 2643 |
|  | LeGene3-CT-R1 | Le3-6R | ATATTTTCATAGTGGGCAGATCC | +37 to +59 |
| *LeALS2721*  (MK332934) | LeGene4-NT-F1 | Le4-1F | CCTCCCACCTACGATGAATTG | -124 to -104 |
|  | LeGene4-TR-F1 | Le4-3F | ATCAACAACTGCCGTGACT | 1023 to 1044 |
|  | LeGene4-NT-R1 | Le4-2R | TGGAAGATGGTTCCTCACTAG | 1327 to 1348 |
|  | LeGene4-TR-Sq-F1 | Le4-10F | GAGAAATCTACTTCCAGTGAGGAA | 1441 to 1464 |
|  | LeGene4-TR-F2 | Le4-5F | GCGAAGAACCTACTACTAGTGAAC | 2177 to 2200 |
|  | LeGene4-TR-Sq-R1 | Le4-13R | CTGTCCCAGTTGGTGGAACATTC | 3636 to 3658 |
|  | LeGene4-TR-R1 | Le4-6R | TGGTTGAGCATTAGTGCTACTT | 3744 to 3765 |
|  | LeGene4-CT-R1 | Le4-8R | ACTGGAATAATGGGAAGATGTCAA | +92 to +115 |
| *LeALS5708*  (MK332935) | LeGene5-NT-F1 | Le5-1F | TGGCAGAAAAGAATCTCCAT | -91 to -72 |
|  | LeGene5-TR-F1 | Le5-3F | AGCTCGAGCAGTGAAGAAATAT | 1231 to 1252 |
|  | LeGene5-NT-R1 | Le5-2R | TGGAGTTGGCTCTTCACTAGA | 1309 to 1329 |
|  | LeGene5-CT-F1 | Le5-5F | CGAAGAATTAACTTCAAGTGAGGAAAC | 2556 to 2582 |
|  | LeGene5-TR-R1 | Le5-4R | GTGGTTGGCTCTTCACAAATAG | 2762 to 2783 |
|  | LeGene5-CT-R1 | Le5-6R | ACAGCAATATCTAGAAGGCAAA | +157 to +178 |
| *LELG_04272*  (OK172378) | LeGene6-Up-F1 | Le6-1F | TCTTGTTCTGGCTGGTAAACTC | -797 to -777 |
|  | LeGene6-Up-R1 | Le6-2R | TCGTGTATGAATTGGTCTGACC | 145 to 166 |
|  | LeGene6-CT-F1 | Le6-3F | CAACATTTTCCACATCGGCT | 9458 to 9477 |
|  | LeGene6 CT-Sq-F1 | Le6-5F | ACTGTCAACTCGTGCAGTAAC | 10668 to 10688 |
|  | LeGene6-CT-Sq-R1 | Le6-4R | CCAGTTGAGATGGAGATTCCAT | 10834 to 10855 |
|  | LeGene6-CT-R1 | Le6-6R | AGGATAGCGACGACACATTATG | +250 to +271 |
| *CauALS2582*  (OK216331) | Cau2582-NT-F1 | Cau1-1F | GTGGAATGCATCTATTGTCTGC | -139 to +118 |
|  | Cau2582-NT-Sq-F1 | Cau1-3F | TCATTCAAGCGATATGTCTGGG | 624 to 645 |
|  | Cau2582-NT-Sq-R1 | Cau1-4R | GAAAATGCGACATTGACGCC | 790 to 809 |
|  | Cau2582-TR-F1 | Cau1-5F | GTCAAGCGGAACTAGGATCG | 1155 to 1174 |
|  | Cau2582-CT-F1 | Cau1-7F | GGCGTTCCATCTATCTCAGTC | 2002 to 2022 |
|  | Cau2582-CT-Sq-R1 | Cau1-6R | CCTTCATTGTTCGTCTTGGC | 2151 to 2168 |
|  | Cau2582-CT-R1 | Cau1-2R | TTGAGCGTACATGGCACG | +139 to +156 |
| *CauALS4112*  (OK216332) | Cau4112-NT-F1 | Cau2-1F | CATAGCGGCTGCAAAAACC | -139 to -121 |
|  | Cau4112-NT-Sq-F1 | Cau2-3F | AACCAGCACTACCTCATGG | 592 to 610 |
|  | Cau4112-TR-F1 | Cau2-4F | CACACGGGCACTGACT | 1114 to 1129 |
|  | Cau4112-TR-R1 | Cau2-6R | TTGTTGCATGGTCAGTCCT | 2191 to 2209 |
|  | Cau4112-TR-F2 | Cau2-5F | GACTGACCATGCAACAACC | 2193 to 2211 |
|  | Cau4112-TR-R2 | Cau2-7R | CAGACTCGAATGGTTCAGTCA | 3248 to 3266 |
|  | Cau4112-TR-R3 | Cau2-8R | GCAGGTGGCTCAGTGAA | 3679 to 3695 |
|  | Cau4112-CT-F1 | Cau2-10F | GACTATTCCACCCTCTGGC | 4266 to 4284 |
|  | Cau4112-CT-Sq-R1 | Cau2-9R | AGGAGTTCTAGGACCAGTAGT | 4441 to 4461 |
|  | Cau4112-CT-R1 | Cau2-2R | TCACCTTGGTGTGAGACATAG | +100 to +120 |
| *CauALS4498*  (OK216333) | Cau4498-NT-F1 | Cau3-1F | TGACGAGCACTTGCTGATG | -108 to -90 |
|  | Cau4498-NT-Sq-F1 | Cau3-3F | TCGTTGGAACCATCACCAAG | 698 to 717 |
|  | Cau4498-NT-Sq-R1 | Cau3-4R | GTCCACATTAATGTATGGACGG | 828 to 849 |
|  | Cau4498-TR-F1 | Cau3-5F | CCATCGTCGTCACTGTTCC | 1073 to 1091 |
|  | Cau4498-CT-Sq-R1 | Cau3-6R | GGATCTAATCGACTGTGGCAC | 2092 to 2112 |
|  | Cau4498-CT-R1 | Cau3-2R | GTCTACAGTGTGTTAAACGAAACTG | +87 to +111 |
| *ClALS3274*  (MH753517) | Cl3274-NT-F1 | CL1-1F | CCGCTGTGTAAGCCCAAA | -121 to -104 |
|  | Cl3274-TR-F1 | CL1-3F | CAGTGTCAGTACGAGCAAGAC | 1110 to 1130 |
|  | Cl3274-NT-R1 | CL1-2R | CACATCCACCTCCACATAAACA | 1257 to 1278 |
|  | Cl3274-SR-F1 | CL1-5F | GCACCGTTGTGGTTGAAATAG | 6287 to 6307 |
|  | Cl3274-TR-R1 | CL1-4R | TCAGTAGACGACACACTAGACC | 6440 to 6461 |
|  | Cl3274-CT-F1 | CL1-7F | GCATCCTACGTCCACTGTATTC | 7563 to 7584 |
|  | Cl3274-SR-R1 | CL1-6R | ACCCACTAGACCCAGATCCT | 7722 to 7741 |
|  | Cl3274-CT-R1 | CL1-8R | AGTGAAATGAAAGGTCCTAGACG | +93 to +113 |
| *YtALS93631*  (MN893379) | Ctn-NT-F1 | Ctn1-1F | GATGATGAAGCGATGGTTTCTAG | -138 to -115 |
|  | Ctn-NT-Sq-F1 | Ctn1-8F | CCCACACTTTCACGGTTATTTC | 500 to 521 |
|  | Ctn-NT-Sq-F2 | Ctn1-9F | CACTTATTCCAGTGGAAGTTCG | 930 to 951 |
|  | Ctn-NT-R1 | Ctn1-2R | GACATTCACCAAGATGGTTTCAG | 1049 to 1072 |
|  | Ctn-TR-F1 | Ctn1-11F | GCTTCAATTGGTAGTACTGCTACT | 1138 to 1161 |
|  | Ctn-TR-Sq-F3 | Ctn1-18F | CTCACCTATACAGGGGAAACTG | 1966 to 1987 |
|  | Ctn-CT-F1 | Ctn1-5F | CGGGTCCTATACCACCACTATTA | 8100 to 8122 |
|  | Ctn-CT-R1 | Ctn1-4R | CGAACTGTTTCTGTATGGTGGTA | 8195 to 8217 |
|  | Ctn-CT-Sq-R1 | Ctn1-10R | CATACAGTGGTTCGGACACC | 8429 to 8448 |
|  | Ctn-CT-Sq-R2 | Ctn1-6R | GGCAGCATCGCCTTCATAA | 9012 to 9030 |
|  | Ctn-CT-Sq-R3 | Ctn1-7R | CCCATGAGTCCTAGCGTAAAG | +646 to +666 |
| *SpALS50348*  (MN893373) | Sp26-NT-F1 | Sp26-1F | CCAATTTCCAAGAAACTTTCCAACT | -136 to -112 |
|  | Sp26-NT-F2 | Sp26-3F | GCTTTGTGCACAGACAGAGA | 409 to 428 |
|  | Sp26-NT-R1 | Sp26-4R | CGAGCAATTAAACACTAAATACACGAG | +226 to +252 |
| *SpALS50349*  (MN893374) | Sp27-UP-F1 | Sp26-5F | GTCAACATGGTGATCAATGGAC | -898 to -877 |
|  | Sp27-UP-F2 | Sp26-7F | TGAGAATGGGCGAGTCATAAAT | -306 to -285 |
|  | Sp27-NT-F2 | Sp26-9F | GTCAAAGGTCAAATTCACATCCC | 364 to 386 |
|  | Sp27-NT-R1 | Sp26-10R | GGGAGTTTCTGATTACAATGGAGTA | +168 to +192 |
| *SpALS55077*  (MN893377) | Sp24-NT-F2 | Sp24-14F | GCATTTTCTTCTCCTGCCAC | 115 to 134 |
|  | Sp24-CT-F1 | Sp24-7F | CACAGGTAGTCAAAGTCACACA | 2862 to 2883 |
|  | Sp24-CT-R2 | Sp24-13R | TGCTACCTCTACCACCAACT | 3828 to 3847 |
| *SpALS59511.5*  (MK332922) | Sp11-NT-F1 | Sp11-1F | CTGCAGATGAGCTCCTGA | -128 to -111 |
|  | Sp11-TR-F1 | Sp11-3F | CACTGGTCTGGTCACTACAG | 1026 to 1045 |
|  | Sp11-NT-R1 | Sp11-2R | GTAGCAGTTTCACCAGGAGTAG | 1175 to 1196 |
|  | Sp11-CT-Sq-R1 | Sp11-7R | TTCCATTATAAGTAGTCGTCACAGT | 4954 to 4978 |
|  | Sp11-CT-R1 | Sp11-6R | CCTAGTCAAACGATTTCAGATAATG | +192 to +216 |
| *SpALS61022.5*  (MK332924) | Sp14-NT-F1 | Sp14-1F | TCAACGTTTAGTTCCCTACTGG | -129 to -108 |
|  | Sp14-NT-Sq-F1 | Sp14-9F | AGTGACGGTGCTAATATTTTGTC | 481 to 503 |
|  | Sp14-TR1-F1 | Sp14-3F | GACTGTGTCTGGTTCTGATACTG | 1650 to 1672 |
|  | Sp14-NT-R1 | Sp14-2R | GCCGGTGGCATCAGTATTA | 1857 to 1875 |
|  | Sp14-TR2-F1 | Sp14-5F | CCGGTGCAACTGGTACTG | 2822 to 2839 |
|  | Sp14-TR1-R1 | Sp14-4R | GTTTGATTGCCTTCGGTGTC | 3040 to 3059 |
|  | Sp14-CT-F1 | Sp14-7F | CTGGTATCAGTTGGTCAACTGAA | 3419 to 3441 |
|  | Sp14-TR2-R1 | Sp14-6R | GGAATCATTACCTTCACTTGTTTCG | 3519 to 3543 |
|  | Sp14-CT-R1 | Sp14-8R | CCATGCGGAATACCACTTAAATAATA | +113 to +138 |
| *SpALS66147*  (MK332932) | Sp25-NT-F1 | Sp25-1F | AAGGAGTCCATGAATAACTTGA | -796 to -775 |
|  | Sp25-NT-R1 | Sp25-2R | CGAGCCTGTCCAAGTATGA | 612 to 630 |
| *SpALS68952*  (MN893376) | Sp29-Gap-F1 | Sp29-5F | ACCTCGTACACAACCATCAC | -879 to -860 |
|  | Sp29-Gap-R1 | Sp29-6R | CGTGGAGCCATCATAGGT | 130 to 147 |
| *SpALS68952.5*  (MN893375) | Sp29-NT-F1 | Sp29-1F | CAACATGAAAGATGTTCTGGATATG | -111 to -87 |
|  | Sp29-NT-F2 | Sp29-12F | CATGTGTACTTAGTCCAGGAGATG | 302 to 325 |
|  | Sp29-TR-F1 | Sp29-3F | AGTTCCAATACCCACTGTTACT | 1104 to 1125 |
|  | Sp29-NT-R1 | Sp29-2R | AAGTAGTTTCACCGCCATATTT | 1351 to 1372 |
|  | Sp29-NT-Sq-F2 | Sp29-10F | GGAAGGACTGCCACGATTAT | 1840 to 1859 |
|  | Sp29-Gap-R2 | Sp29-9R | CAGGCTCTCCACTTTCACTAC | +317 to +337 |
| *SpALS134590*  (MN893372) | Sp19-TR2-F2 | Sp19-27F | GAGACTGGAGAACCAGCA | 2644 to 2661 |
|  | Sp19-TR3-F1 | Sp19-7F | GATCCTGAGATTGGCGAAACTAT | 3838 to 3860 |
|  | Sp19-TR2-R2 | Sp19-28R | ATAGTTTCGCCAATCTCAGGATC | 3838 to 3860 |
|  | Sp19-TR2-R1 | Sp19-6R | ATCTCCAGGTCCTGCACTAA | 4205 to 4224 |
|  | Sp19-TR3-R2 | Sp19-23F | GGACTCCAATTGTCCCAAATA | 4550 to 4570 |
|  | Sp19-CT-F2 | Sp19-11F | AAGTCCTATTAGATCTGGATCTGGA | 4968 to 4992 |
|  | Sp19-CT-R2 | Sp19-12R | GTTGGACATGGAAGGGAGTAAA | 5688 to 5707 |
| *SpALS134590.5*  (MN893378) | Sp20-NT-F1 | Sp20-1F | GAGTGGGTGTTTCTGAAACAG | -242 to -222 |
|  | Sp20-TR1-F1 | Sp20-3F | TAAATCCTTGGTCCGATGGTC | 830 to 850 |
|  | Sp20-NT-R1 | Sp20-2R | CGACGTCGTGGTACCATC | 1207 to 1224 |
|  | Sp20-TR2-F2 | Sp20-12F | CGTTACCTTACGATCCTGAGATTG | 1604 to 1627 |
|  | Sp20-TR2-F1 | Sp20-5F | ACTCTCTTGGTACTGCTACTGA | 2120 to 2141 |
|  | Sp20-TR2-R1 | Sp20-6R | CCTGGTCGTGCTCCTG | 3767 to 3782 |
|  | Sp20-CT-Sq-F1 | Sp20-14F | CCGTAGGAACACGTCCAGA | 4034 to 4052 |
|  | Sp20-CT-Sq-R2 | Sp20-10R | TATACCAGTAGGACTTCCACTTCC | 4237 to 4260 |
|  | Sp20-CT-Sq-R3 | Sp20-11R | AGGTCAAAGTAGCTATATCATGTC | +162 to +185 |
| *SpALS140900*  (MK332917) | Sp5-RP-F1 | Sp5-3F | ATTTGTTCGCCGTGCTGA | 1254 to 1271 |
|  | Sp5-CT-F1 | Sp5-5F | AATCCTCCGGCGCTAATA | 2858 to 2875 |
|  | Sp5-CT-R1 | Sp5-6R | AAGCTACTTATGGCGATATACA | +94 to +115 |
| *SpALS156463*  (MK332933) | Sp28-NT-F1 | Sp28-1F | TTGCAAAGAAGCCCTAGGATATT | -109 to -87 |
|  | Sp28-TR-F1 | Sp28-3F | TCTGCTACGGATGAAGTAACTG | 1213 to 1234 |
|  | Sp28-NT-R1 | Sp28-2R | CAGGACCAATGGGTCCAG | 1334 to 1352 |
|  | Sp28-CT-Sq-F1 | Sp28-7F | TCCTGGTCCTCAAGGTCAAG | 2415 to 2434 |
|  | Sp28-CT-Sq-R1 | Sp28-8R | GGCCACCTTGACCTTGAG | 2423 to 2440 |
|  | Sp28-TR-R1 | Sp28-4R | CATCAGTACCTCCTTGACCATC | 2670 to 2692 |
|  | Sp28-CT-R1 | Sp28-6R | CACAAAGTTTATCAGTTGCTTGAAC | +259 to +283 |
| *SsALS2386*  (MH753518) | Ss2386-NT-F1 | Ss1-1F | CCTTCGTAAGGAGTTGGTTTCT | -139 to -118 |
|  | Ss2386-TR-F1 | Ss1-3F | CCAACTACAACAATCACCCAAAC | 1087 to 1109 |
|  | Ss2386-NT-R1 | Ss1-2R | TATCAGTTCCGCCATGTGTATC | 1267 to 1288 |
|  | Ss2386-CT-F1 | Ss1-5F | GTACCGATACTGTGATTGTTGAAAT | 5078 to 5102 |
|  | Ss2386-TR-R1 | Ss1-4R | ACCGTGGACACTGTTGAAA | 5291 to 5309 |
|  | Ss2386-CT-R1 | Ss1-6R | GGAATCAGTAGCCTTGTTTATACC | +99 to +122 |
| *SsALS2786*  (MH753519) | Ss2786-NT-F1 | Ss3-1F | TGGCTAATAAGCAACCTCACA | -103 to -83 |
|  | Ss2786-TR-F1 | Ss3-3F | AGGCGAATCAACGAACACTAT | 1038 to 1058 |
|  | Ss2786-NT-R1 | Ss3-2R | GTGATAGTGGTGGTTGGGTTAG | 1196 to 1217 |
|  | Ss2786-CT-F1 | Ss3-5F | ATTGAGGTACCATCCGTTTCAG | 7771 to 7792 |
|  | Ss2786-TR-R1 | Ss3-4R | GACTTGTGTGAGCTTGTGAATC | 7948 to 7969 |
|  | Ss2786-CT-R1 | Ss3-6R | ACGCGTGTTTCAGTAGTCAG | +135 to +154 |
| *SsALS4579*  (MH753520) | Ss4579-NT-F1 | Ss5-1F | CTTGAGTGCCTCGTCTAGATTTG | -154 to -132 |
|  | Ss4579-TR-F1 | Ss5-3F | GTGGCCAGGATTTCGTAGTT | 893 to 912 |
|  | Ss4579-NT-R1 | Ss5-2R | GGGCATTCTTCAGTAGTTGAGG | 1292 to 1313 |
|  | Ss4579-CT-F1 | Ss5-5F | CAATCCCTTCCTCTAGCATTGA | 1871 to 1892 |
|  | Ss4579-TR-R1 | Ss5-4R | AGAGTAGTAGTGGTCTCACTGG | 2063 to 2084 |
|  | Ss4579-CT-R1 | Ss5-6R | TCGGACGGCATAGTGAATC | +91 to +109 |
| *MgALS673*  (MH753516) | Mg673-NT-F1 | Mg4-1F | CCTCCCAATTCAACATCAAATG | -91 to -70 |
|  | Mg673-CR-F1 | Mg4-3F | ACTCCGACTCCACTGGAAAT | 947 to 966 |
|  | Mg673-NT-R1 | Mg4-2R | TGGTGGTAGTTCTGTCTGTATC | 1078 to 1099 |
|  | Mg673-CT-F1 | Mg4-5F | TCTAGCGTTTCGAAGACATCAA | 1597 to 1618 |
|  | Mg673-CR-R1 | Mg4-4R | CAGCGACTTGTGACACTCTT | 1727 to 1744 |
|  | Mg673-CT-R1 | Mg4-6R | GATAAGGAATAGCGCCAGATGA | +106 to +127 |
| *MgALS2302*  *(*MH753513) | Mg2299-NT-F1 | Mg1-1F | GCCCTATACTGCGTCATGTT | -111 to -92 |
|  | Mg2299-TR-F1 | Mg1-3F | TGGTAATACCGGTGGAAATGG | 957 to 977 |
|  | Mg2299-NT-R1 | Mg1-2R | GTGAAGGTGGAAGTGGTTGTA | 1140 to 1160 |
|  | Mg2299-TR-Sq-F1 | Mg1-11F | GACTGGAACTGGCTCTACAATC | 1653 to 1674 |
|  | Mg2299-TR-Sq-F2 | Mg1-12F | GGAACTGCTACGGTGATTGT | 3589 to 3608 |
|  | Mg2299-TR-Sq-R1 | Mg1-13R | GAGTGTGGTGGTCGAAACTT | 4289 to 4308 |
|  | Mg2299-SR-F1 | Mg1-5F | TGGACTGGAACTGAAACTTCTAC | 7009 to 7031 |
|  | Mg2299-TR-R1 | Mg1-4R | GACTGGACTCAGAAACTGTCAA | 7228 to 7249 |
|  | Mg2299-SR-Sq-F1 | Mg1-9R | AGCTCTGCTTCATCTTCGG | 7519 to 7537 |
|  | Mg2299-SR-Sq-R1 | Mg1-10R | GGTGTACCACTTGAGGATACTG | 8465 to 8486 |
|  | Mg2299-CT-F1 | Mg1-7F | GGGAAATGGCGGAACAGATA | 8832 to 8851 |
|  | Mg2299-SR-R1 | Mg1-6R | TTTGACCGTAGCCTCCATTAC | 8963 to 8983 |
|  | Mg2299-CT-R1 | Mg1-8R | TTGGCGTCTAACGTCGTAAAG | +118 to +138 |
| *MgALS3259*  (MH753514) | Mg3259-NT-F1 | Mg2-1F | CCGCAGGCCTAGGATCA | -138 to -122 |
|  | Mg3259-TR-F1 | Mg2-3F | GATTTGACCACCAGGATCTC | 1069 to 1088 |
|  | Mg3259-NT-R1 | Mg2-2R | ATGGTGGTGGTGGTTGTAATAG | 1187 to 1208 |
|  | Mg3259-CT-F1 | Mg2-5F | CAGTTAAAGGTGGACCAGGTAG | 5402 to 5423 |
|  | Mg3259-TR-R1 | Mg2-4R | GGAGGTGAGGGTAGTTCTT | 5562 to 5580 |
|  | Mg3259-CT-Sq-R1 | Mg2-7R | GATGAAGGGTCCACGG | 6374 to 6389 |
|  | Mg3259-CT-Sq-R2 | Mg2-9R | CTAGGGTCTCCTCCGTTACTT | 6666 to 6686 |
|  | Mg3259-CT-R1 | Mg2-6R | CCCAATTGAGTAGCCCAAGATA | +72 to +93 |
| *MgALS3330*  (MH753515) | Mg3300-NT-F1 | Mg3-1F | TATGGCTCGTTTGGGATATGG | -152 to -132 |
|  | Mg3300-TR-F1 | Mg3-3F | CCAGAGTCACTACTTTGCCATAC | 1025 to 1047 |
|  | Mg3300-NT-R1 | Mg3-2R | GTCTTGGTGGTGGTAACAGTAT | 1113 to 1154 |
|  | Mg3300-TR-Sq-F1 | Mg3-11F | CAGGAGGAACCAACACAGTAG | 1799 to 1819 |
|  | Mg3300-TR-Sq-R1 | Mg3-12R | CAGTCACAGTGGTGGTGTTAG | 2297 to 2317 |
|  | Mg3300-CT-F1 | Mg3-5F | TACTACAACTATCACCGCTCCTCCT | 2628 to 2652 |
|  | Mg3300-TR-R1 | Mg3-4R | ACGAAACAGACGAGACAGAAAG | 2764 to 2785 |
|  | Mg3300-CT-Sq-F1 | Mg3-7F | CCTTCTGGACCTTCTTCTTC | 3097 to 3115 |
|  | Mg3300-CT-Sq-R1 | Mg3-8R | TACCACCATTCCCACCATTC | 3990 to 4009 |
|  | Mg3300-CT-R1 | Mg3-6R | TGCTCTCGGTGCATTTCCTAAT | +73 to +94 |
| *DhALS2178*  (MH753526) | Dh2178-NT-F1 | Dh1-1F | AACATGTGTTTCATAAGCCTCG | -120 to -99 |
|  | Dh2178-TR-F1 | Dh1-3F | CATAACAGAGACGGAAACTACAT | 1065 to 1087 |
|  | Dh2178-NT-R1 | Dh1-2R | ACAACTTCACTACTTGTCTTTGTTG | 1262 to 1286 |
|  | Dh2178-CT-F1 | Dh1-5F | CTTGTCAGGACTGTGTAAGTTC | 1661 to 1682 |
|  | Dh2178-TR-R1 | Dh1-4R | GCCAGTTTCCAGTGGTTC | 1837 to 1854 |
|  | Dh2178-CT-R1 | Dh1-6R | CACGACTTGTCTGTTCCTATATTT | +81 to +104 |

*Primer location was relative to the GenBank accession number listed in the first column. Negative signs indicated primers that were upstream of the coding region while plus signs denoted primers downstream of the coding region. Sequences upstream and downstream of the coding region were located on *Candida* Genome Database ([www.candidagenome.org](http://www.candidagenome.org)) or from larger contigs in GenBank.

Superscripts were added throughout the table to explain differences between initial primers and validated data resulting from Sanger sequencing.

^1^Primer sequence is shown; Sanger sequencing revealed TG following C in the coding region.
