# Supplementary material for: Using Genomics to Shape the Definition of the Agglutinin-Like Sequence (ALS) Family in the Saccharomycetales
Source: Front Cell Infect Microbiol. 2021 Dec 14;11:794529. doi: 10.3389/fcimb.2021.794529 (PMC8712946; doi:10.3389/fcimb.2021.794529)
Supplement: Supplementary file 14 [file Table_2.docx]

**Supplementary Table S2** | *ALS* genes and characteristics of their predicted proteins from 16 fungal species.

| **Gene** | **GenBank Accession** | **Size (nt)** | **Size (aa)** | **Sig Pep Size (aa)** | **NT-Als Size (aa)** | **NT-Als Cys (#)*** | **Inv. Lys**^†^ | **Relevant AFR? (Location)** ^‡^ | **Repeat Region** | **CT %Ser/Thr**^§^ | **GPI** ^¶^ | **Reference** |  |
| --- | --- | --- | --- | --- | --- | --- | --- | --- | --- | --- | --- | --- | --- |
| ***Candida albicans* SC5314** | | | | | | | | | | | | | |
| *CaALS1* | XM_712984 | 3783 | 1260 | 17 | 313 | 8 | Lys | E (325-330) | Tandem, 36 aa | 45 | -22 | Muzzey et al., 2013 |  |
| *CaALS2* | XM_707553; AH006927 | 7089 | 2362 | 17 | 312 | 8 | Lys | E (324-329) | Tandem, 36 aa | 36 | -22 | Muzzey et al., 2013; Hoyer et al., 1998 |  |
| *CaALS3* | AY223552 | 3468 | 1155 | 17 | 313 | 8 | Lys | E (325-330) | Tandem, 36 aa | 42 | -22 | Zhao et al., 2004 |  |
| *CaALS4* | XM_705333 | 6303 | 2100 | 17 | 313 | 8 | Lys | E (325-330) | Tandem, 36 aa | 37 | -22 | Muzzey et al., 2013 |  |
| *CaALS5* | AY227440 | 4152 | 1383 | 19 | 311 | 8 | Lys | E (325-330) | Tandem, 36 aa | 45 | -22 | Zhao et al., 2003 |  |
| *CaALS6* | AY225310 | 4101 | 1366 | 18 | 315 | 8 | Lys | E (328-333) | Tandem, 36 aa | 45 | -22 | Zhao et al., 2007 |  |
| *CaALS7* | XM_710972 | 4707 | 1568 | 18 | 314 | 8 | Lys | None | Tandem, 36 aa | 41 | -19 W | Muzzey et al., 2013 |  |
| *CaALS9-1* | AY269423 | 5565 | 1854 | 17 | 313 | 8 | Lys | E (325-330) | Tandem, 36 aa | 43 | -22 | Zhao et al., 2003 |  |
| *CaALS9-2* | AY269422 | 5502 | 1833 | 17 | 313 | 8 | Lys | E (325-330) | Tandem, 36 aa | 42 | -22 | Zhao et al., 2003 |  |
| ***Candida dubliniensis* CD36** | | | | | | | | | | | | | |
| *CdALS64210* | XM_002421088 | 3888 | 1295 | 17 | 313 | 8 | Lys | E (325-330) | Tandem, 36 aa | 43 | -22 | Jackson et al., 2009 |  |
| *CdALS64220* | XM_002421089 | 5742 | 1913 | 17 | 313 | 8 | Lys | E (324-330) | Tandem, 36 aa | 43 | -22 | Jackson et al., 2009 |  |
| *CdALS64610* | XM_002421123 | 5970 | 1989 | 17 | 316 | 8 | Lys | E (323-333) | Tandem, 36 aa | 35 | -22 | Jackson et al., 2009 |  |
| *CdALS64800* | XM_002421132 | 8025 | 2674 | 17 | 313 | 8 | Lys | E (324-330) | Tandem, 36 aa | 35 | -22 | Jackson et al., 2009 |  |
| *CdALS65010* | XM_002421149 | 4353 | 1450 | 17 | 313 | 8 | Lys | E (324-330) | Tandem, 36 aa | 35 | -22 | Jackson et al., 2009 |  |
| *CdALS86150* | XM_002419473 | 15456 | 5151 | 18 | 312 | 8 | Lys | None | Tandem, 36 aa | 46 | -19 | Jackson et al., 2009 |  |
| *CdALS86290* | XM_002419486 | 5043 | 1680 | 18 | 316 | 8 | Lys | E (328-324) | Tandem, 36 aa | 41 | -22 | Jackson et al., 2009 |  |
| ***Candida tropicalis* MYA-3404** | | | | | | | | | | | | | |
| *CtrALS941* | MH753531 | 8865 | 2954 | 19 | 313 | 8 | Lys | None | Tandem, most 36 aa | 33 | -23 | Oh et al., 2021 |  |
| *CtrALS1028* | MH753521 | 3192 | 1063 | 19 | 314 | 8 | Lys | E (327-333) | Tandem, 35-37 aa | 42 | -23 | Oh et al., 2021 |  |
| *CtrALS1030* | MH753522 | 3963 | 1320 | 19 | 312 | 5 | Lys | E (326-332) | Tandem, 36-37 aa | 31 | -22 | Oh et al., 2021 |  |
| *CtrALS1038* | MK128125 | 3204 | 1067 | 19 | 310 | 8 | Lys | E (324-329) | Tandem, 37 aa | 37 | -22 | Oh et al., 2021 |  |
| *CtrALS1041* | MK128127 | 4200 | 1399 | 19 | 313 | 8 | Lys | E (327-332) | Tandem, 37 aa | 42 | -22 | Oh et al., 2021 |  |
| *CtrALS2228* | MK128126 | 4872 | 1623 | 18 | 320 | 8 | Lys | E (328-338) | Tandem, 36 aa | 38 | -22 | Oh et al., 2021 |  |
| *CtrALS2229* | MH753523 | 5406 | 1801 | 19 | 315 | 8 | Lys | None | Tandem, 36 aa | 31 | -23 | Oh et al., 2021 |  |
| *CtrALS2293* | MK182724 | 2925 | 974 | 19 | 315 | 8 | Lys | E (326-334) | Tandem, 31 aa | 47 | -24 | Oh et al., 2021 |  |
| *CtrALS3786* | MK332912 | 6030 | 2009 | 19 | 315 | 8 | Lys | E (326-334) | Tandem, 35-36 aa | 51 | -23 W | Oh et al., 2021 |  |
| *CtrALS3791* | MK170233 | 3171 | 1056 | 19 | 315 | 8 | Lys | E (326-334) | Tandem, 36 aa | 46 | -22 | Oh et al., 2021 |  |
| *CtrALS3797* | MN224675 | 8850 | 2949 | 19 | 311 | 8 | Lys | W (326-330) | Tandem, 35-36 aa; short | 38 | -27 | Oh et al., 2021 |  |
| *CtrALS3871* | MH753524 | 4968 | 1655 | 19 | 313 | 10 | Lys | E (327-332) | Tandem, 36 aa | 38 | -23 | Oh et al., 2021 |  |
| *CtrALS3882-1* | MH753525 | 4833 | 1610 | 19 | 311 | 8 | Lys | E (325-330) | Tandem, 36 aa | 38 | -23 | Oh et al., 2021 |  |
| *CtrALS3882-2* | MN893367 | 4440 | 1479 | 19 | 313 | 8 | Lys | E (327-332) | Tandem, 36 aa | 37 | -23 | Oh et al., 2021 |  |
| ***Lodderomyces elongisporus* NRRL YB-4239** | | | | | | | | | | | | | |
| *LeALS734* | MN893368 | 3450 | 1149 | 21 | 319 | 6 | Lys | None | Tandem; 41 aa; Iff/Hyr | 36 | -22 | This work |  |
| *LeALS2536* | MN893369 | 4767 | 1588 | 17 | 331 | 8 | Lys | E (333-348) | Short, 2 types | 43 | -24 | This work |  |
| *LeALS2716* | MN893370 | 5403 | 1800 | 17 | 320 | 8 | Arg | None | Tandem, 41-47 aa; Iff/Hyr | 28 | -23 | This work |  |
| *LeALS2721* | MK332934 | 4056 | 1351 | 17 | 320 | 8 | Lys | None‡ | Short, 2 types | 39 | -25 | This work |  |
| *LeALS5708* | MK332935 | 3309 | 1102 | 17 | 320 | 8 | Lys | W (333-337) | Short, irregular | 41 | -32 | This work |  |
| ***Candida parapsilosis* CDC 317** | | | | | | | | | | | | | |
| *CpALS4770* | MH753532 | 3003 | 1000 | 22 | 321 | 8 | Lys | W (335-343) | Short, irregular | 37 | -23 | Oh et al., 2019 |  |
| *CpALS4780* | MH753533 | 3450 | 1149 | 22 | 322 | 8 | Lys | W (336-344) | Short, 2 types | 39 | -23 | Oh et al., 2019 |  |
| *CpALS4790* | BK010629 | 7179 | 2392 | 22 | 322 | 8 | Lys | E (334-344) | Tandem, 36 aa | 45 | -22 | Oh et al., 2019 |  |
| *CpALS4800* | BK010630 | 4152 | 1383 | 22 | 323 | 8 | Lys | W (337-345) | Tandem, 36 aa | 40 | -21 | Oh et al., 2019 |  |
| *CpALS660* | MH753534 | 3072 | 1023 | 22 | 322 | 8 | Lys | W (336-344) | Short, 2 types | 44 | -24 | Oh et al., 2019 |  |
| ***Candida orthopsilosis* Co 90-125** | | | | | | | | | | | | | |
| *CoALS4210* | MG799558 | 2457 | 818 | 22 | 322 | 8 | Lys | W (336-344) | Short, 2 types | 43 | -23 | Oh et al., 2019 |  |
| *CoALS4220* | MG799559 | 6078 | 2025 | 22 | 320 | 8 | Lys | W (334-341) | Tandem, 34-36 aa | 45 | -21 | Oh et al., 2019 |  |
| *CoALS800* | MG799557 | 2499 | 832 | 22 | 318 | 8 | Lys | W (336-340) | Short | 47 | -23 | Oh et al., 2019 |  |
| ***Candida metapsilosis* ATCC 96143** | | | | | | | | | | | | | |
| *CmALS4210-1* | MH753528 | 4722 | 1573 | 22 | 323 | 8 | Lys | W (337-345) | Short, 2 types | 44 | -24 | Oh et al., 2019 |  |
| *CmALS4220-1* | MH753512 | 6714 | 2237 | 22 | 322 | 8 | Lys | E (334-344) | Tandem, 36 aa | 43 | -23 | Oh et al., 2019 |  |
| *CmALS800* | MH753530 | 3234 | 1077 | 22 | 319 | 8 | Lys | W (336-341) | Short, 2 types | 33 | -23 | Oh et al., 2019 |  |
| *CmALS2265* | MH765692 | 6489 | 2162 | 22 | 321 | 8 | Lys | None | Tandem, 42 aa; Iff/Hyr | 41 | -23 | Oh et al., 2019 |  |
| ***Candida auris* B8441** | | | | | | | | | | | | | |
| *CauALS2582* | OK216331 | 2586 | 861 | 19 | 318 | 6 | None | E (333-337) | None | 40 | -21 W | This work |  |
| *CauALS4112* | OK216332 | 5388 | 1795 | 18 | 321 | 6 | Lys | E (333-338) | Tandem; 34-39 aa | 33 | -23 | This work |  |
| *CauALS4498* | OK216333 | 2520 | 839 | 19 | 320 | 11 | Lys | E (333-339) | None | 42 | -34 | This work |  |
| ***Clavispora (Candida) lusitaniae* ATCC 42720** | | | | | | | | | | | | | |
| *ClALS3274* | MH753517 | 8358 | 2785 | 18 | 317 | 4 | Lys | None | 67-102 aa | 41 | -23 | This work |  |
| ***Yamadazyma (Candida) tenuis* strain ATCC 10573** | | | | | | | | | | | | | |
| *YtALS93631* | MN893379 | 9090 | 3029 | 18 | 310 | 8 | Lys | E (323-328) | Tandem, 32-45 aa | 41 | -22 | This work |  |
| ***Spathaspora passalidarum* NRRL Y-27907** | | | | | | | | | | | | | |
| *SpALS49824* | MK332931 | 1893 | 630 | 19 | 312 | 8 | Lys | None | None | 35 | -32 W | This work |  |
| *SpALS50348* | MN893373 | 1008 | 335 | 17 | 312 | 14 | Lys | None | No CT domain | N/A | N/A | This work |  |
| *SpALS50349* | MN893374 | 1008 | 335 | 18 | 307 | 8 | Lys | None | No CT domain | N/A | N/A | This work |  |
| *SpALS55077* | MN893377 | 4134 | 1377 | 17 | 321 | 11 | Lys | W (334-338) | PIPT repeats; 72-101 aa | 30 | -27 | This work |  |
| *SpALS59511.5* | MK332922 | 5667 | 1888 | 17 | 349 | 8 | None | AW (361-366) | PIPT repeats; 75-89 aa | 25 | -27 | This work |  |
| *SpALS61022.5* | MK332924 | 4407 | 1468 | 17 | 344 | 8 | Lys | A (356-361) | Short, irregular | 36 | -31 W | This work |  |
| *SpALS64434* | MK332915 | 2691 | 896 | 16 | 341 | 8 | Arg | W (326-330) | None | 35 | -25 | This work |  |
| *SpALS64435* | MK332916 | 1836 | 611 | 16 | 315 | 7 | Lys | W (327-331) | None | 34 | -23 | This work |  |
| *SpALS66147* | MK332932 | 4116 | 1371 | N/A | N/A | N/A | Lys | N/A | PIPT repeats; 72-243 aa | N/A | -26 | This work |  |
| *SpALS68952* | MN893376 | 6189 | 2062 | N/A | N/A | N/A | Lys | N/A | PIPT repeats; 80-243 aa | N/A | -23 | This work |  |
| *SpALS68952.5* | MN893375 | 2757 | 918 | 17 | 350 | 8 | Lys | A (363-367) | PIPT repeats; 72-80 aa | N/A | N/A | This work |  |
| *SpALS131476* | MK332914 | 3378 | 1125 | 16 | 317 | 8 | Lys | None | Short, irregular | 29 | -21 | This work |  |
| *SpALS134426* | MK332928 | 1449 | 482 | 19 | 341 | 8 | Lys | E (330-334) | N/A | N/A | N/A | This work |  |
| *SpALS134590* | MN893372 | 5760 | 1919 | 17 | 336 | 10 | Lys | W (334-338) | PIPT repeats; 72-125 aa | 29 | -27 | This work |  |
| *SpALS134590.5* | MN893378 | 4671 | 1556 | N/A | N/A | N/A | Lys | N/A | PIPT repeats; 72-101 aa | 28 | -27 | This work |  |
| *SpALS134874* | MK332920 | 4245 | 1414 | 17 | 317 | 8 | Lys | None | Short, irregular | 38 | -20 | This work |  |
| *SpALS135549* | MK332921 | 5733 | 1910 | 17 | 317 | 8 | Lys | None | Short, irregular | 42 | -20 | This work |  |
| *SpALS136382* | MK332929 | 3261 | 1086 | 17 | 344 | 8 | Lys | A (356-361) | Short, irregular | 41 | -19 | This work |  |
| *SpALS137089* | MK332930 | 3654 | 1217 | 22 | 311 | 8 | Lys | W (329-333) | Short, irregular | 27 | -23 | This work |  |
| *SpALS138016* | MK332925 | 2199 | 732 | 15 | 315 | 9 | Lys | E (326-330) | None | 39 | -22 | This work |  |
| *SpALS140483* | MK332919 | 4995 | 1664 | 16 | 317 | 8 | Lys | None | 68-104 aa | 37 | -18 W | This work |  |
| *SpALS140900* | MK332917 | 4098 | 1365 | 17 | 317 | 8 | Lys | AW (367-374) | Short | 37 | -21 W | This work |  |
| *SpALS141433* | MK332918 | 3285 | 1094 | 17 | 278 | 7 | Lys | AW (367-377)‡ | None | 38 | -20 | This work |  |
| *SpALS146555* | MK332913 | 2319 | 772 | 17 | 343 | 8 | Lys | A (355-360) | Glu repeats | 29 | -24 | This work |  |
| *SpALS152224* | MK332923 | 6738 | 2245 | 17 | 344 | 8 | Lys | A (356-361) | None | 41 | -19 | This work |  |
| *SpALS153035* | MK332926 | 2025 | 674 | 17 | 342 | 8 | Lys | A (354-359) ‡ | None | 33 | -24 | This work |  |
| *SpALS153035.5* | MK332927 | 2394 | 797 | 24 | 283 | 8 | Lys | None ‡ | Glu repeats | 33 | -26 | This work |  |
| *SpALS155003* | MN893371 | 2568 | 855 | 15 | 297 | 8 | Lys | None | None | 27 | -19 | This work |  |
| *SpALS156463* | MK332933 | 3390 | 1130 | 17 | 319 | 8 | Lys | E (331-336) | Short, 2 types | 21 | -23 | This work |  |
| ***Scheffersomyces (Pichia) stipitis* CBS 6054** | | | | | | | | | | | | | |
| *SsALS2386* | MH753518 | 5811 | 1936 | 16 | 319 | 8 | Lys | E (330-335) | Tandem; 35-42 aa | 35 | -23 | This work |  |
| *SsALS2786* | MH753519 | 8385 | 2794 | 16 | 316 | 8 | Lys | E (327-332) | Tandem; 36 aa | 32 | -23 | This work |  |
| *SsALS4579* | MH753520 | 3159 | 1052 | 14 | 321 | 6 | Arg | None | None | 51 | -30 | This work |  |
| ***Meyerozyma (Candida) guilliermondii* ATCC 6260** | | | | | | | | | | | | | |
| *MgALS673* | MH753516 | 2439 | 812 | 22 | 311 | 6 | Arg | E (326-333) | None | 41 | -19 | This work |  |
| *MgALS2302* | MH753513 | 9681 | 3226 | 18 | 316 | 6 | Lys | W (329-334) | Tandem, 34-36 aa | 49 | -25 | This work |  |
| *MgALS3259* | MH753514 | 6963 | 2320 | 16 | 315 | 6 | Lys | W (326-331) | Tandem, 34-36 aa | 46 | -25 | This work |  |
| *MgALS3330* | MH753515 | 5469 | 1822 | 16 | 316 | 8 | None | None | Tandem, 35-36 aa; short | 38 | -22 | This work |  |
| ***Debaryomyces hansenii* strain CBS 767** | | | | | | | | | | | | | |
| *DhALS2178* | MH753526 | 2256 | 751 | 20 | 310 | 6 | Arg | None | None | 42 | -19 | This work |  |
| ***Candida glabrata* CBS 138** | | | | | | | | | | | | | |
| CAGL0G04125g | XM_446542.1 | 2292 | 763 | 18 | 323 | 7 | None | None | None | 61 | -23 | Xu et al., 2020 |  |
| ***Saccharomyces cerevisiae* S288C** | | | | | | | | | | | | | |
| *ScSAG1* | NM_001181661 | 1953 | 650 | 19 | 304 | 6 | Arg | W (319-323) | None | 40 | -24 | Goffeau et al., 1996 |  |

* Number of Cys in the mature NT-Als domain (i.e. secretory signal peptide removed; see **Supplementary File S5** for sequences).

† The invariant Lys in NT-Als is located at the end of the binding pocket (Lin et al., 2016). The positive charge sinks a negative charge from the C-terminal carboxyl group of a peptide ligand. Some NT-Als sequences have an Arg at this location; others do not have a positively charged amino acid in this position (marked “None”).

‡ Amyloid-forming potential was assessed using β-aggregation scores from Tango (tango.crg.es; Fernandez-Escamilla et al., 2004). The amyloid-forming region was demonstrated experimentally in *C. albicans* Als proteins (Ho et al., 2019). Amino acid numbering started at the N-terminal Met. E = expected amyloid-forming potential (typical scores were approximately 90); W = weak amyloid-forming potential (score of 10 to 35, an arbitrary designation); A = alternative potential AFR location (i.e. a strong β-aggregation score C-terminal to the expected AFR location); None = lack of a relevant sequence with amyloid-forming potential. In addition to some proteins possessing an AFR around amino acid 360, others had one near amino acids 280-290. These latter examples are marked with ‡.

§ Percent Ser/Thr in the C-terminal region was calculated using the ProtParam tool (<https://web.expasy.org/protparam>; Gasteiger et al., 2005). Sequences immediately following the repeated region were used. In cases where no repeated region existed, the sequence following NT-Als was used. For LeAls2716, only 53 aa were considered since the protein ended just after the repeat region.

¶ GPI anchor addition was predicted using big-PI Fungal Predictor (<https://mendel.imp.ac.at/gpi/fungi_server.html>; Eisenhaber et al., 2004). The amino acid with the greatest potential for GPI addition is identified. W = lack of a definitive GPI addition site; the best scoring amino acid was noted.
